# Smoking exposures and mortality risk in individuals with idiopathic pulmonary fibrosis: a UK Biobank cohort study

**DOI:** 10.3389/fpubh.2026.1884390

**Published:** 2026-09-16

**Authors:** Qiyun Tu, Xinyue Zhang, Yuxin Zou, Yuqing Lv, Lifeng Yan, Chenchen Hou, Lina Wang, Wenhui Sun, Haiyang Zhang, Manyi Pan, Tianyu Zhou, Baopeng Liu, Xiahui Ge, Huaqi Guo, Weining Xiong

**Affiliations:** 1 Department of Respiratory and Critical Care Medicine, Shanghai Ninth People’s Hospital, Shanghai Jiao Tong University School of Medicine, Shanghai, China; 2 Department of Epidemiology, School of Public Health, Cheeloo College of Medicine, Shandong University, Jinan, Shandong, China; 3 School of Medicine, Shanghai University, Shanghai, China; 4 Institute of Respiratory Diseases, Shanghai Jiao Tong University School of Medicine, Shanghai, China

**Keywords:** additive interaction, idiopathic pulmonary fibrosis, mortality, secondhand smoke, smoking exposure, UK Biobank

## Abstract

**Background:**

Smoking and secondhand smoke (SHS) exposures are established modifiable risk factors for premature mortality, but their joint associations with recorded idiopathic pulmonary fibrosis (IPF) remain uncertain. We examined active and passive smoking exposures in relation to all-cause and cause-specific mortality and evaluated departures from additivity with recorded IPF history.

**Methods:**

This prospective UK Biobank cohort included 270,644 participants. Exposures comprised baseline smoking status, cumulative pack-years, maternal smoking around birth, adult SHS, and a composite passive SHS indicator. Multivariable Cox models, additive and multiplicative interaction analyses, and restricted cubic spline models were used after adjustment for demographic, socioeconomic, lifestyle, and genetic covariates.

**Results:**

During a median follow-up of 13.8 years, 20,959 deaths occurred. Ever-recorded IPF history was associated with all-cause mortality (HR 4.255, 95% CI 3.937–4.597), and active and passive smoking measures were also associated with mortality. In the additive interaction analyses, a positive departure from additivity was observed only between maternal smoking around birth and recorded IPF in relation to respiratory mortality (RERI 16.969, 95% CI 2.935–31.002; AP 0.248, 95% CI 0.082–0.414; SI 1.337, 95% CI 1.068–1.673). Corresponding positive RERI estimates were observed in analyses restricted to White participants, among participants with complete covariate data, and after excluding deaths occurring within the first 2 years of follow-up. The confidence intervals for the other RERI estimates in the primary analysis included zero. Dose–response associations were nonlinear among participants without recorded IPF and approximately linear among those with recorded IPF, although estimates in the latter group were less precise.

**Conclusion:**

Active and passive smoking measures and ever-recorded IPF history were associated with mortality in this cohort. Evidence for positive additive interaction was confined to maternal smoking around birth and respiratory mortality and should be interpreted as hypothesis-generating. The observational findings do not establish causal or biological synergy and require replication in independent cohorts.

## 1 Introduction

Smoking remains a leading preventable cause of morbidity and mortality worldwide and continues to represent a major public health challenge (1). In addition to active smoking, secondhand smoke (SHS) exposure across different life stages contributes substantially to the burden of cardiovascular disease, cancer, and chronic respiratory disease (2). Because smoking and SHS exposure are common and preventable, understanding their long-term health effects is important for population-level prevention and tobacco control strategies (3).

Idiopathic pulmonary fibrosis (IPF) is a progressive fibrosing interstitial lung disease associated with impaired lung function, reduced quality of life, and high mortality (4–12). Cigarette smoking has consistently been identified as an important environmental risk factor for the development and progression of IPF. Individuals with IPF may be more vulnerable to the harmful effects of smoking exposure because of underlying respiratory impairment (13–15). However, evidence regarding the combined effects of smoking exposure and IPF on long-term mortality remains limited (16–18).

Growing evidence shows that smoking exposure during different stages of life may influence respiratory health across the life course. Maternal smoking and adult SHS exposure have been associated with impaired lung development, reduced lung function, and increased risk of chronic respiratory disease (19–21). Previous studies have also reported associations between early-life smoking exposure and incident IPF (22, 23). Nevertheless, few large prospective studies have evaluated the joint associations of active smoking and SHS exposure with all-cause and cause-specific mortality among individuals with IPF.

Evaluating the joint associations of smoking exposure and recorded IPF can clarify whether their observed mortality associations depart from additivity and whether cumulative smoking shows different dose–response patterns across IPF strata (24–27). Therefore, using data from the UK Biobank, we aimed to examine associations of active and passive smoking measures with all-cause and cause-specific mortality, quantify additive and multiplicative interaction with recorded IPF history, and assess dose–response associations between cumulative pack-years and mortality (28).

## 2 Methods

### 2.1 Study design and participants

This population-based prospective cohort study used data from the UK Biobank, which recruited more than 500,000 participants between 2006 and 2010 from 22 assessment centers across the United Kingdom. At baseline, participants completed touchscreen questionnaires and computer-assisted interviews. The UK Biobank study was approved by the North West Multi-centre Research Ethics Committee (REC reference: 21/NW/0157), and all participants provided written informed consent. Further details of the cohort have been described previously (28).

In the present study, 502,301 participants were initially included. We excluded participants who were lost to follow-up or withdrew consent (*n* = 1,297), those without genetic data (*n* = 15,134), and those without linked ICD-10 data (*n* = 53,287). Participants with self-reported or ICD-10-diagnosed secondary pulmonary fibrosis at baseline were also excluded (*n* = 30,043). In addition, participants with missing smoking exposure data (*n* = 109,525) or missing covariate data (*n* = 22,371) were excluded. After these exclusions, 270,644 participants were included in the final analyses (Figure 1).

**Figure 1:**
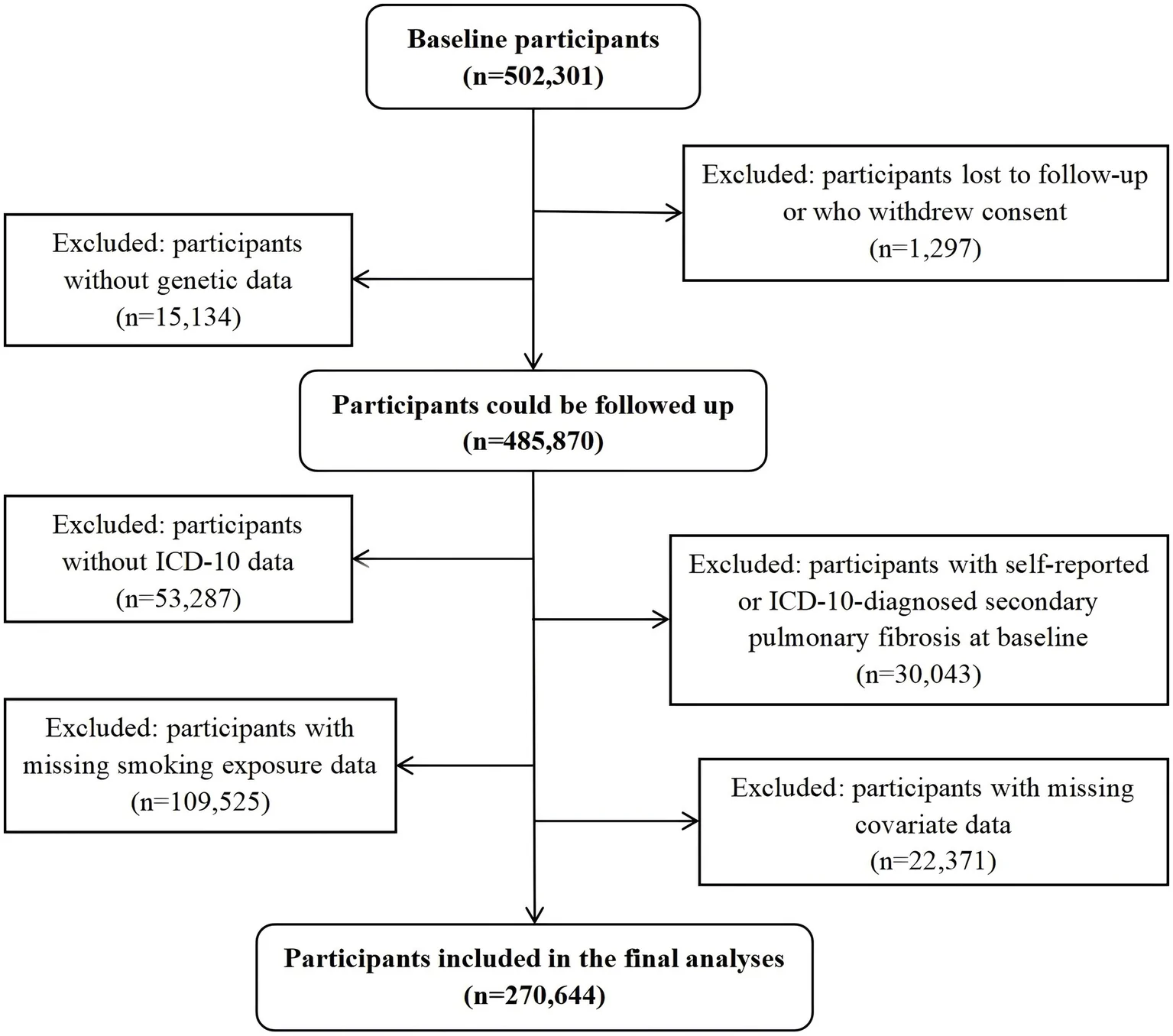
Study screening and analytical sample selection flow diagram. ICD-10, International Classification of Diseases, 10th Revision; IPF, idiopathic pulmonary fibrosis.

### 2.2 Ascertainment of IPF

Recorded IPF was ascertained from linked hospital inpatient and death-registry records using ICD-10 code J84.1, together with questionnaire evidence of physician-diagnosed IPF from the UK Biobank online follow-up assessment (Fields 22135, 22155, and 22175). For registry-derived cases, the earliest qualifying J84.1 record was used as the diagnosis date; for questionnaire-derived cases, diagnosis timing was estimated from the reported age at diagnosis. When both sources were available, the earliest diagnosis date was used. Participants with prespecified secondary causes of pulmonary fibrosis were excluded as detailed in Supplementary Table S3 (22, 29–33).

### 2.3 Assessment of smoking and secondhand smoke exposure

Smoking and SHS exposures were assessed at multiple stages of life. Maternal smoking around birth was assessed using UK Biobank Field ID 1787 (21, 22), which asked participants whether their mother smoked regularly around the time they were born. This measure was based on participant recall at the baseline assessment. Adult smoking status was self-reported at baseline and categorized as never, former, or current smoking. Cumulative active smoking intensity was quantified continuously using pack-years derived from the smoking history reported at baseline. For the stratified, joint, and interaction analyses, smoking status was dichotomized as current versus non-current smoking, with never and former smokers combined in the non-current category. Adult SHS exposure was defined as exposure for more than 0 h per week at home or outside the home, based on UK Biobank Fields 1269 and 1279. Field 1259 was interpreted as baseline household smoking. A composite passive SHS indicator classified participants as exposed if any of maternal smoking around birth, adult SHS exposure, or baseline household smoking was present. All smoking exposure variables were defined using information collected at baseline and were not updated during follow-up (21, 34).

### 2.4 Outcome ascertainment

The dates and causes of death were obtained through linkage with the death registries of the National Health Service (NHS) Information Centre for participants from England and Wales and the NHS Central Register Scotland for participants from Scotland (28). Follow-up for mortality analyses was defined from the date of recruitment to the date of death or the censoring date, whichever occurred first. The primary outcome was all-cause mortality, defined as death from any cause. Cause-specific mortality outcomes included cardiovascular, cancer, and respiratory mortality based on ICD-10 codes (Supplementary Table S2).

### 2.5 Covariates

Multivariable regression models adjusted for demographic, socioeconomic, and lifestyle covariates to reduce potential confounding. Covariates included age at recruitment, sex, ethnicity, Townsend deprivation index quartiles, household income, educational attainment, body mass index (BMI), physical activity, alcohol consumption, and diet quality. We additionally adjusted for six candidate single nucleotide polymorphisms (SNPs) previously associated with idiopathic pulmonary fibrosis (rs59424629, rs35705950, rs184498750, rs60514164, rs35647788, and rs3893252; Supplementary Table S4) as covariates to account for potential genetic confounding; genetic associations were not evaluated in this study (31, 35). Operational definitions and classification of all covariates are provided in the online supplement (Supplementary Table S5). Participants with missing values for covariates with less than 5% missingness were excluded. For covariates with more than 5% missing values, an “unknown” category was created to retain participants in the analyses.

### 2.6 Statistical analysis

Baseline characteristics were compared using the chi-square (*χ*^2^) test for categorical variables and the Wilcoxon rank-sum test for continuous variables. To assess potential selection related to missing data, we separately compared baseline characteristics between participants included in the analytical cohort and those excluded because of missing exposure or covariate data. Continuous variables were compared using Welch’s *t*-tests and categorical variables using Pearson’s chi-square tests. Standardized mean differences were calculated, with an absolute value of 0.10 or greater considered indicative of potential imbalance. Variable-specific missing proportions were compared separately using two-sample tests of proportions. Cox proportional hazards models were used to estimate hazard ratios (HRs) and 95% confidence intervals (CIs) for the associations of smoking exposures and overall IPF status with all-cause and cause-specific mortality. Each smoking exposure was examined in a separate Cox model to estimate associations and avoid over-adjustment for overlapping exposures.

For joint analyses, participants were classified into four groups according to the presence or absence of the smoking-related exposure and recorded IPF. Additive interaction was evaluated using the relative excess risk due to interaction (RERI), attributable proportion due to interaction (AP), and synergy index (SI). The corresponding 95% CIs were calculated using the delta method and the full model-based variance–covariance matrix. Evidence of a positive departure from additivity was considered present when the 95% CI for RERI excluded zero in the positive direction; AP and SI were reported as complementary measures. Multiplicative interaction was assessed by including a product term between each smoking-related exposure and recorded IPF in the Cox model. Cause-specific interaction analyses were considered secondary and exploratory (24–27). For the joint and interaction analyses, adult smoking status was modeled as current versus non-current smoking, with never and former smokers combined in the non-current category. Passive smoking exposures were modeled as exposed versus unexposed (25). Estimates of RERI, AP, and SI are reported in the tables.

Restricted cubic spline (RCS) Cox models were used to examine dose–response associations between cumulative smoking (pack-years) and mortality, stratified by overall IPF status. Pack-years were evaluated separately from the categorical joint analyses because they represent cumulative active smoking exposure on a continuous scale. Non-linearity was tested using likelihood ratio tests comparing models with linear versus spline terms.

Several sensitivity analyses were conducted. First, analyses were repeated among participants reporting White ethnicity. Second, complete-case analyses were performed among participants without missing covariate data. Third, deaths occurring within the first 2 years of follow-up were excluded. Fourth, ever-recorded IPF history was separated into prevalent IPF before baseline and incident IPF after baseline. Fifth, IPF was modeled as a time-varying exposure: person-time before the earliest recorded IPF diagnosis date, derived as described above, was classified as unexposed, and person-time from that date onward was classified as exposed. All analyses were conducted in R version 4.5.3. Statistical significance was defined as a two-sided *p* value <0.05.

## 3 Results

### 3.1 Baseline characteristics

Among 270,644 participants, 1,331 (0.5%) had a recorded history of IPF. Baseline demographic, socioeconomic, and smoking exposure characteristics are summarized in Table 1. Compared with the non-IPF group, participants with IPF had shorter follow-up periods (median: 4,697 vs. 5,030 days; *p* < 0.001), were older at baseline (median: 64.0 vs. 58.0 years; *p* < 0.001), and were more likely to be male (64.8% vs. 45.0%, *p* < 0.001). Regarding socioeconomic and lifestyle factors, the IPF group had higher proportions of participants in the lowest household income bracket (<£18,000; 30.9% vs. 17.5%), the most deprived Townsend deprivation quartile (Q4; 26.2% vs. 21.4%), and with obesity (35.7% vs. 24.2%) compared with the non-IPF group (all *p* < 0.001). For smoking exposures, former or current active smoking was more common in participants with IPF (64.4% vs. 40.4%, *p* < 0.001). Maternal smoking exposure (32.8% vs. 29.2%, *p* < 0.01) and adult SHS exposure (28.6% vs. 22.1%, *p* < 0.001) were also more frequent in the IPF group. To assess potential selection related to missing data, we compared the 270,644 participants included in the analytical cohort with the 131,896 participants excluded because of missing exposure or covariate data. Differences between the two groups were relatively small for age, BMI, sex, and IPF status, whereas more apparent differences were observed in smoking status, ethnicity, alcohol consumption, and socioeconomic characteristics, including household income, education, and Townsend deprivation (Supplementary Table S6).

**Table 1:** Baseline characteristics of participants with and without IPF.

Characteristics	Overall (*n* = 270,644)	Non-IPF (*n* = 269,313)	IPF (*n* = 1,331)	*p*-value
Age, median (IQR)	58.0 (50.0–63.0)	58.0 (50.0–63.0)	64.0 (61.0–67.0)	*p* < 0.001
Follow-up time (days), median (IQR)	5,030 (4,766–5,289)	5,030 (4,767–5,291)	4,697 (3,725.5–5,116.5)	*p* < 0.001
Sex, *n* (%)				*p* < 0.001
Female	148,606 (54.9%)	148,137 (55.0%)	469 (35.2%)	
Male	122,038 (45.1%)	121,176 (45.0%)	862 (64.8%)	
BMI category, *n* (%)				*p* < 0.001
Normal weight	88,171 (32.6%)	87,906 (32.6%)	265 (19.9%)	
Obese	65,616 (24.2%)	65,141 (24.2%)	475 (35.7%)	
Overweight	115,722 (42.8%)	115,135 (42.8%)	587 (44.1%)	
Underweight	1,135 (0.4%)	1,131 (0.4%)	4 (0.3%)	
Household income, *n* (%)				*p* < 0.001
18,000–30,999	60,071 (22.2%)	59,751 (22.2%)	320 (24.0%)	
31,000–51,999	64,555 (23.9%)	64,311 (23.9%)	244 (18.3%)	
52,000–100,000	52,067 (19.2%)	51,959 (19.3%)	108 (8.1%)	
Greater than 100,000	13,702 (5.1%)	13,675 (5.1%)	27 (2.0%)	
Less than 18,000	47,580 (17.6%)	47,169 (17.5%)	411 (30.9%)	
Unknown	32,669 (12.1%)	32,448 (12.0%)	221 (16.6%)	
Education level, *n* (%)				*p* < 0.001
Any school degree	103,092 (38.1%)	102,703 (38.1%)	389 (29.2%)	
College or University degree	97,178 (35.9%)	96,894 (36.0%)	284 (21.3%)	
Unknown	40,872 (15.1%)	40,424 (15.0%)	448 (33.7%)	
Vocational qualification or other	29,502 (10.9%)	29,292 (10.9%)	210 (15.8%)	
Ethnicity, *n* (%)				*p* < 0.05
Non-white	11,741 (4.3%)	11,701 (4.3%)	40 (3.0%)	
White	258,903 (95.7%)	257,612 (95.7%)	1,291 (97.0%)	
Townsend deprivation, *n* (%)				*p* < 0.001
Q1	73,426 (27.1%)	73,114 (27.1%)	312 (23.4%)	
Q2	71,454 (26.4%)	71,117 (26.4%)	337 (25.3%)	
Q3	67,912 (25.1%)	67,579 (25.1%)	333 (25.0%)	
Q4	57,852 (21.4%)	57,503 (21.4%)	349 (26.2%)	
Physical activity, *n* (%)				*p* < 0.001
Insufficient	44,803 (16.6%)	44,538 (16.5%)	265 (19.9%)	
Sufficient	213,473 (78.9%)	212,503 (78.9%)	970 (72.9%)	
Unknown	12,368 (4.6%)	12,272 (4.6%)	96 (7.2%)	
Alcohol consumption, *n* (%)				*p* < 0.001
Excessive drinking	62,745 (23.2%)	62,419 (23.2%)	326 (24.5%)	
Moderate drinking	120,052 (44.4%)	119,551 (44.4%)	501 (37.6%)	
Non-drinker	87,847 (32.5%)	87,343 (32.4%)	504 (37.9%)	
Diet quality, *n* (%)				*p* < 0.001
Healthy	149,596 (55.3%)	148,925 (55.3%)	671 (50.4%)	
Unhealthy	121,048 (44.7%)	120,388 (44.7%)	660 (49.6%)	
Smoking status, *n* (%)				*p* < 0.001
Current	7,684 (2.8%)	7,629 (2.8%)	55 (4.1%)	
Former	101,930 (37.7%)	101,128 (37.6%)	802 (60.3%)	
Never	161,030 (59.5%)	160,556 (59.6%)	474 (35.6%)	
Maternal smoking exposure, *n* (%)				*p* < 0.01
No	191,448 (70.7%)	190,553 (70.8%)	895 (67.2%)	
Yes	79,196 (29.3%)	78,760 (29.2%)	436 (32.8%)	
Adult SHS exposure, *n* (%)				*p* < 0.001
No	210,708 (77.9%)	209,758 (77.9%)	950 (71.4%)	
Yes	59,936 (22.1%)	59,555 (22.1%)	381 (28.6%)	

Categorical variables are presented as *n* (%) and continuous variables as median (interquartile range). Groups were compared using Pearson’s chi-square test for categorical variables and the Wilcoxon rank-sum test for continuous variables. Percentages may not total 100% because of rounding.

BMI, body mass index; IPF, idiopathic pulmonary fibrosis; IQR, interquartile range; SHS, secondhand smoke.

### 3.2 Independent associations of smoking exposures and overall IPF status with mortality

During follow-up, 20,959 deaths occurred. Multivariable-adjusted Cox proportional hazards models showed that a history of IPF was associated with a substantially higher risk of all-cause mortality. In the fully adjusted model (Model 3), participants with IPF had more than a four-fold higher risk of all-cause mortality compared to those without IPF (HR: 4.255, 95% CI: 3.937–4.597). This increased risk was consistent across cause-specific mortality outcomes, including cardiovascular, cancer, and respiratory mortality (Table 2).

**Table 2:** Independent associations of smoking-related exposures and IPF with all-cause mortality during follow-up.

Exposure	Person-years (PYs)	Case/N	Incidence rate (95% CI)	HR (95% CI)		
				Model 1	Model 2	Model 3
IPF						
No	3629780.01	20,277/269313	5.586 (5.509–5.663)	1.000 (ref)	1.000 (ref)	1.000 (ref)
Yes	15583.11	682/1331	43.765 (40.481–47.050)	8.395 (7.778–9.061)***	4.274 (3.957–4.616)***	4.255 (3.937–4.597)***
Smoking status						
Never	2182639.77	10,008/161030	4.585 (4.495–4.675)	1.000 (ref)	1.000 (ref)	1.000 (ref)
Former	1359574.98	10,270/101930	7.554 (7.408–7.700)	1.658 (1.613–1.704)***	1.259 (1.224–1.296)***	1.259 (1.224–1.296)***
Current	103148.37	681/7684	6.602 (6.106–7.098)	1.444 (1.336–1.561)***	1.464 (1.353–1.583)***	1.463 (1.353–1.582)***
Adult SHS exposure						
Never	2839213.94	15,702/210708	5.530 (5.444–5.617)	1.000 (ref)	1.000 (ref)	1.000 (ref)
Ever	806149.18	5257/59936	6.521 (6.345–6.697)	1.178 (1.142–1.215)***	1.126 (1.091–1.162)***	1.126 (1.091–1.162)***
Maternal smoking exposure						
Never	2577889.42	14,800/191448	5.741 (5.649–5.834)	1.000 (ref)	1.000 (ref)	1.000 (ref)
Ever	1067473.70	6159/79196	5.770 (5.626–5.914)	1.003 (0.974–1.034)	1.047 (1.015–1.079)**	1.046 (1.015–1.078)**
Passive SHS exposure						
Never	1930660.54	10,725/143325	5.555 (5.450–5.660)	1.000 (ref)	1.000 (ref)	1.000 (ref)
Ever	1714702.58	10,234/127319	5.968 (5.853–6.084)	1.073 (1.044–1.103)***	1.097 (1.067–1.128)***	1.096 (1.067–1.127)***
Pack-years						
0	2187982.75	10,080/161443	4.607 (4.517–4.697)	1.000 (ref)	1.000 (ref)	1.000 (ref)
1–10	269806.52	1255/19929	4.651 (4.394–4.909)	1.011 (0.953–1.072)	0.972 (0.916–1.031)	0.972 (0.916–1.031)
11–20	259054.69	1654/19304	6.385 (6.077–6.692)	1.391 (1.321–1.466)***	1.134 (1.076–1.196)***	1.134 (1.076–1.196)***
21–30	164546.36	1331/12346	8.089 (7.654–8.523)	1.765 (1.667–1.869)***	1.271 (1.199–1.346)***	1.270 (1.199–1.346)***
>30	198646.20	3152/15535	15.867 (15.313–16.421)	3.516 (3.378–3.659)***	1.926 (1.846–2.009)***	1.925 (1.845–2.009)***
Unknown	565326.60	3487/42087	6.168 (5.963–6.373)	1.344 (1.293–1.397)***	1.146 (1.102–1.191)***	1.146 (1.102–1.191)***

Cases/N indicates the number of deaths/total number of participants. Incidence rates are presented per 1,000 person-years, and “ref” indicates the reference category. Hazard ratios (HRs) and 95% CIs were estimated using Cox proportional hazards regression. Model 1 was unadjusted. Model 2 was adjusted for sex, age at recruitment, body mass index (BMI), household income, educational attainment, ethnicity, Townsend deprivation index quartiles, physical activity (PA), alcohol consumption, and diet. Model 3 was additionally adjusted for candidate SNPs. Each smoking-related exposure was analyzed in a separate Cox model. Pack-years were categorized as 0, 1–10, 11–20, 21–30, and >30; “Unknown” indicates missing pack-years information. Passive SHS exposure was defined as a composite measure of secondhand smoke exposure. Statistical significance is indicated as *p < 0.05, **p < 0.01, and ***p < 0.001.

BMI, body mass index; CI, confidence interval; HR, hazard ratio; IPF, idiopathic pulmonary fibrosis; PA, physical activity; PYs, person-years; SHS, secondhand smoke; SNP, single nucleotide polymorphism.

After multivariable adjustment, smoking exposures at different life stages remained associated with mortality. Compared with never smokers, current smokers at baseline had the highest risk of all-cause mortality (HR: 1.463, 95% CI: 1.353–1.582), followed by former smokers (HR: 1.259, 95% CI: 1.224–1.296). For environmental tobacco smoke exposures, the fully adjusted HRs for all-cause mortality were 1.126 (95% CI, 1.091–1.162) for adult SHS exposure, 1.046 (95% CI, 1.015–1.078) for maternal smoking around birth, and 1.096 (95% CI, 1.067–1.127) for the composite passive SHS indicator. Their associations with cardiovascular, cancer, and respiratory mortality are reported in Supplementary Table S7.

### 3.3 Joint and interactive associations of smoking exposures and overall IPF status

In joint models (Figure 2), the highest risks of all-cause mortality were generally observed among participants with both a smoking exposure and a recorded history of IPF, compared with the unexposed, non-IPF reference group. Participants with smoking exposure but without IPF had modestly elevated hazard ratios, whereas participants with IPF had substantially higher mortality regardless of smoking exposure. Similar joint hazard-ratio patterns were observed for cardiovascular, cancer, and respiratory mortality (Supplementary Table S9).

**Figure 2:**
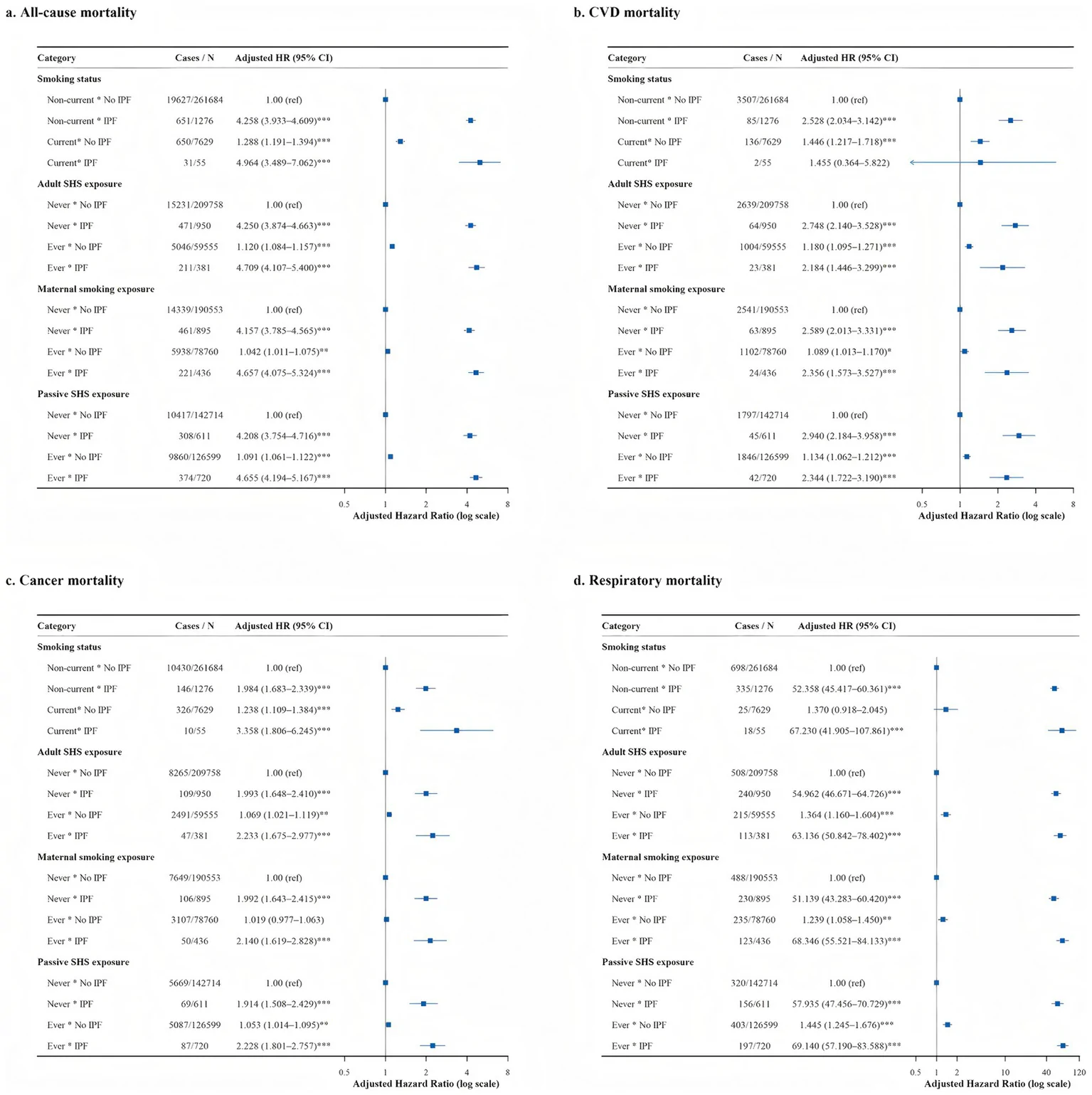
Joint associations of smoking-related exposures and recorded IPF with all-cause and cause-specific mortality during follow-up. **(A)** All-cause mortality, **(B)** Cardiovascular disease (CVD) mortality, **(C)** Cancer mortality, and **(D)** Respiratory mortality. Hazard ratios (HRs) and 95% confidence intervals (CIs) were estimated using multivariable-adjusted Cox proportional hazards models, with the unexposed, non-IPF group as the reference (“ref”). Models were adjusted for age at recruitment, sex, ethnicity, body mass index (BMI), Townsend deprivation index quartiles, household income, educational attainment, physical activity, alcohol consumption, diet quality, and six candidate IPF-associated SNPs. BMI, body mass index; CI, confidence interval; CVD, cardiovascular disease; HR, hazard ratio; IPF, idiopathic pulmonary fibrosis; PA, physical activity; SNP, single nucleotide polymorphism.

Additive interactions were evaluated using RERI, AP, and SI. For all-cause mortality, the 95% CIs for all RERI estimates included zero. The 95% CIs for all RERI estimates for cardiovascular and cancer mortality also included zero. For respiratory mortality, only maternal smoking exposure showed evidence of a positive departure from additivity with recorded IPF (RERI 16.969, 95% CI 2.935–31.002; AP 0.248, 95% CI 0.082–0.414; SI 1.337, 95% CI 1.068–1.673). The RERI estimates for smoking status, adult SHS exposure, and passive SHS exposure in relation to respiratory mortality had 95% CIs that included zero (Table 3). No statistically significant multiplicative interactions were observed in the primary analyses (Supplementary Table S10).

**Table 3:** Additive interactions between smoking-related exposures and IPF in relation to all-cause and cause-specific mortality during follow-up.

Additive interaction	Index for additive interaction	All-cause mortality	CVD mortality	Cancer mortality	Respiratory mortality
Smoking status	RERI	0.417 (−1.364–2.198)	−1.519 (−3.622–0.584)	1.135 (−0.975–3.246)	14.502 (−17.248–46.252)
	AP	0.084 (−0.246–0.414)	−1.044 (−3.907–1.818)	0.338 (−0.085–0.761)	0.216 (−0.158–0.590)
	SI	1.118 (0.712–1.755)	0.230 (0.003–19.623)	1.929 (0.762–4.879)	1.280 (0.789–2.078)
Adult SHS exposure	RERI	0.339 (−0.405–1.083)	−0.743 (−1.866–0.380)	0.171 (−0.571–0.912)	7.810 (−6.020–21.640)
	AP	0.072 (−0.078–0.222)	−0.340 (−0.970–0.290)	0.076 (−0.237–0.390)	0.124 (−0.075–0.322)
	SI	1.100 (0.895–1.352)	0.614 (0.267–1.413)	1.161 (0.619–2.175)	1.144 (0.908–1.440)
Maternal smoking exposure	RERI	0.458 (−0.265–1.181)	−0.322 (−1.465–0.820)	0.129 (−0.576–0.834)	**16.969 (2.935–31.002)**
	AP	0.098 (−0.046–0.243)	−0.137 (−0.668–0.394)	0.060 (−0.255–0.376)	0.248 (0.082–0.414)
	SI	1.143 (0.930–1.405)	0.808 (0.365–1.788)	1.128 (0.593–2.145)	1.337 (1.068–1.673)
Passive SHS exposure	RERI	0.356 (−0.311–1.023)	−0.730 (−1.849–0.388)	0.261 (−0.391–0.912)	10.761 (−2.792–24.314)
	AP	0.077 (−0.061–0.214)	−0.312 (−0.853–0.230)	0.117 (−0.158–0.392)	0.156 (−0.024–0.335)
	SI	1.108 (0.914–1.343)	0.648 (0.331–1.269)	1.270 (0.694–2.324)	1.188 (0.957–1.473)

Additive interactions between smoking-related exposures and IPF in relation to all-cause and cause-specific mortality during follow-up were evaluated using RERI, AP, and SI with 95% confidence intervals (CIs) from Model 3. Model 3 was adjusted for sex, age at recruitment, body mass index (BMI), household income, educational attainment, ethnicity, Townsend deprivation index quartiles, physical activity (PA), alcohol consumption, diet, and candidate SNPs. For smoking status, the exposed category was current smoking and the reference category was non-current smoking, which included never and former smokers. For passive smoking exposures, exposed participants were compared with unexposed participants. Boldface indicates RERI estimates for which the 95% confidence interval does not include zero.

AP, attributable proportion; BMI, body mass index; CI, confidence interval; CVD, cardiovascular disease; IPF, idiopathic pulmonary fibrosis; PA, physical activity; RERI, relative excess risk due to interaction; SHS, secondhand smoke; SI, synergy index; SNP, single nucleotide polymorphism.

### 3.4 Dose–response continuous associations stratified by overall IPF status

Restricted cubic spline models were used to examine the dose–response associations between cumulative active smoking (pack-years) and mortality, adjusted for covariates and stratified by recorded IPF status (Figure 3). Among participants without IPF, cumulative pack-years were associated with statistically significant nonlinear increases in all-cause, cardiovascular, cancer, and respiratory mortality (all p for nonlinearity < 0.001). No formal change-point or threshold analysis was performed; therefore, the spline curves were not used to define a discrete pack-year cutoff. Among participants with IPF, mortality risk increased approximately linearly with cumulative pack-years, and tests for nonlinearity were not statistically significant across outcomes. Estimates in this group were less precise because of the smaller sample size.

**Figure 3:**
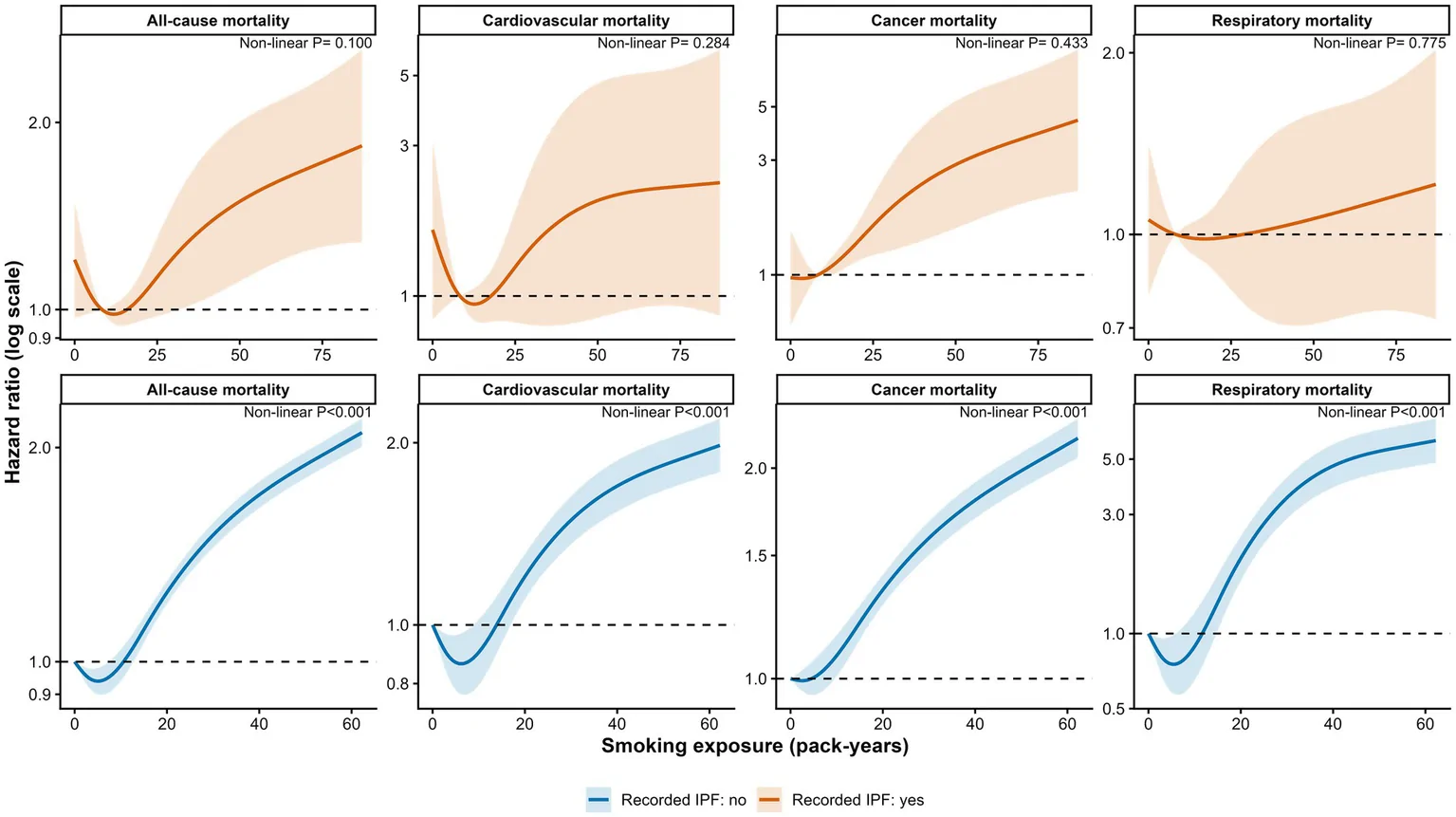
Continuous dose–response associations between cumulative active smoking exposure (pack-years) and mortality, stratified by IPF status. Restricted cubic spline (RCS) Cox models were used to estimate continuous dose–response associations of cumulative active smoking (pack-years) with mortality. Solid lines represent point estimates of hazard ratios (HRs), shaded areas represent 95% confidence intervals (CIs), and horizontal dashed lines indicate the reference (*HR* = 1.0). Non-linear *p*-values were obtained using likelihood ratio tests comparing spline and linear models. Top panels (orange) indicate participants with a recorded history of IPF; bottom panels (blue) indicate participants without IPF. Models were adjusted for age at recruitment, sex, ethnicity, BMI, Townsend deprivation index quartiles, household income, educational attainment, physical activity, alcohol consumption, diet quality, and candidate IPF genetic risk variants. Abbreviations: CI, confidence interval; CVD, cardiovascular disease; *HR*, hazard ratio; IPF, idiopathic pulmonary fibrosis; RCS, restricted cubic spline; SNP, single nucleotide polymorphism.

### 3.5 Sensitivity analyses

Several sensitivity analyses were conducted to assess the robustness of the findings. The positive additive interaction between maternal smoking exposure and recorded IPF in relation to respiratory mortality was also observed among White participants (RERI 17.438, 95% CI 3.482–31.393; Supplementary Table S13), among participants with complete covariate data (RERI 30.999, 95% CI 2.809–59.190; Supplementary Table S17), and after excluding deaths occurring within the first 2 years of follow-up (RERI 16.113, 95% CI 1.811–30.415; Supplementary Table S21). Most other additive interaction estimates had 95% CIs that included zero. In the complete-case analysis, an inverse departure from additivity was observed between adult SHS exposure and recorded IPF for cardiovascular mortality (RERI −1.888, 95% CI −3.504 to −0.272); however, this finding was not reproduced in the primary analysis or the other sensitivity analyses and was therefore interpreted cautiously. IPF history was stratified into baseline prevalent and post-baseline incident cases; both subgroups showed independently and jointly elevated risks of all-cause and cause-specific mortality, with slightly higher hazard estimates in the baseline prevalent cohort (Supplementary Table S23). When recorded IPF was modeled as a time-varying exposure, the joint association patterns were directionally similar, although the additive interaction estimates were less precise. The positive additive interaction between maternal smoking around birth and time-varying IPF for respiratory mortality remained evident, whereas the other additive interaction estimates were imprecise and included the null (Supplementary Tables S24–S26).

## 4 Discussion

In this large prospective cohort, ever-recorded IPF history and several active and passive smoking measures were associated with all-cause and cause-specific mortality. Participants with both recorded IPF and smoking exposure generally had the highest hazard ratios, although the association with recorded IPF was substantially larger than the associations with the smoking measures. The additive interaction analyses did not indicate widespread positive departures from additivity. Evidence of a positive departure from additivity was confined to maternal smoking around birth and recorded IPF in relation to respiratory mortality. Dose–response analyses showed nonlinear associations between pack-years and mortality among participants without recorded IPF and approximately linear, although less precise, associations among those with recorded IPF. The study therefore adds information on life-course smoking exposure, joint mortality patterns with recorded IPF, and cumulative smoking that is not captured by smoking status alone.

The association between IPF and mortality is well established, with prior studies showing poor prognosis despite antifibrotic treatment (4–10, 36). Our results are consistent with these findings and extend prior evidence by showing that life-course smoking exposures, particularly current smoking and cumulative pack-years, were associated with increased all-cause, cardiovascular, cancer, and respiratory mortality after adjustment for measured socioeconomic factors and the candidate genetic variants included in the models. The approximately linear dose–response pattern among participants with recorded IPF is compatible with an incremental association with cumulative smoking, although estimates were less precise because of the small IPF subgroup (37, 38). Furthermore, prognosis in IPF is also shaped by comorbidity burden. In patients with mild-to-moderate IPF, Sonaglioni et al. found that the Charlson Comorbidity Index was associated with all-cause mortality and that a score of 6 or higher identified patients at increased risk (39). Other studies have shown that survival varies according to the overall number and type of comorbidities. Cardiovascular disease, pulmonary hypertension, diabetes mellitus, chronic kidney disease, gastroesophageal reflux disease, and lung cancer are common in IPF, although their prognostic associations differ across conditions and studies (15, 40, 41). Because smoking is also related to several of these diseases, its association with mortality should be interpreted as one component of a broader clinical profile. Our analyses did not examine interactions between smoking and individual comorbidities. Clinical care should therefore combine smoking cessation support with identification and appropriate management of relevant comorbidities.

We also examined passive smoking exposures at different stages of life. Adult SHS exposure and the composite passive SHS indicator were associated with higher all-cause and cause-specific mortality. Maternal smoking exposure was associated with elevated mortality risk across several outcomes, although the magnitude and precision of the associations differed by cause of death. The magnitudes of these associations were generally modest and broadly comparable across the passive smoking measures, without clear evidence of heterogeneity according to exposure timing. However, direct comparisons should be interpreted cautiously because the exposure measures differed in timing and ascertainment, and no formal comparisons were performed. Previous life-course evidence has shown that early-life exposures can influence long-term health, including respiratory outcomes (23, 42–44). Maternal smoking exposure likely reflects prenatal or early-life exposure, which has been associated with impaired lung development and reduced lung function (34, 45). In our analyses, passive smoking measures were associated with mortality across several outcomes, and maternal smoking around birth was the only exposure that showed a positive additive interaction with recorded IPF for respiratory mortality.

Only maternal smoking around birth showed a positive additive interaction with recorded IPF for respiratory mortality. The finding was also present in the White-participant, complete-case, two-year exclusion, and time-varying IPF analyses, although these analyses used overlapping samples and the estimates varied in precision. The other respiratory mortality RERI estimates were positive but had confidence intervals that included zero, and no multiplicative interactions were detected. This finding raises the possibility that early-life tobacco-smoke exposure may be relevant to later respiratory vulnerability. However, maternal smoking was reported retrospectively, and the large RERI partly reflects the strong association between recorded IPF and respiratory mortality. It should therefore be viewed as an epidemiologic signal requiring external confirmation, rather than evidence of a causal biological interaction (24–27).

This study has several methodological strengths. We used the UK Biobank, a large prospective population-based cohort with standardized baseline assessments, long-term follow-up, and detailed multi-stage active and passive smoking measures (28). Linkage to hospital episode statistics and national death registries enabled standardized capture of recorded IPF diagnoses and cause-specific mortality, and the exclusion of secondary pulmonary fibrosis improved diagnostic specificity (9, 28, 46). We also conducted a detailed assessment of life-course smoking exposures, capturing cumulative active smoking intensity and multiple passive SHS measures across mortality endpoints. Multivariable restricted cubic spline models were used to evaluate continuous, non-linear dose–response associations stratified by IPF status. We also assessed pack-years continuously and repeated the main analyses using alternative sample restrictions and definitions of IPF timing. The joint hazard-ratio patterns were generally similar across these analyses. The maternal-smoking interaction for respiratory mortality was also observed in the White-participant, complete-case, two-year exclusion, and time-varying IPF analyses, although precision differed across analyses.

This study has several limitations. First, maternal smoking around birth and cumulative smoking pack-years were based on retrospective self-reports collected at baseline and may therefore be subject to recall error and exposure misclassification. Second, recorded IPF was ascertained from linked hospital and death-registry records and questionnaire-reported physician diagnoses. This multi-source approach broadened case capture beyond reliance on administrative records alone. However, participant-level high-resolution computed tomography, pulmonary function data, and multidisciplinary diagnostic adjudication were unavailable. Questionnaire-based diagnoses may additionally be affected by recall error, incomplete participation in the online follow-up assessment, and imprecision in the reported age at diagnosis. Residual diagnostic and temporal misclassification may therefore have affected the hazard ratios and interaction estimates, and the direction of bias is uncertain. Third, smoking behaviors and environmental tobacco smoke exposures were measured only at baseline and were not updated during the median follow-up of 13.8 years. The analyses therefore do not account for subsequent smoking cessation, relapse, or changes in household and environmental secondhand smoke exposure. Although IPF status was evaluated as a time-varying exposure in a sensitivity analysis, smoking exposures were not updated over time. Finally, the UK Biobank cohort is predominantly White and healthier than the general population, which may limit generalizability. Exclusions due to missing exposure or covariate data may have introduced selection bias because participants included in the analytical cohort differed from those excluded, particularly in smoking status and socioeconomic characteristics. The White-participant analysis assessed internal consistency but did not address healthy-volunteer bias or constitute external validation. In addition, multiple interaction estimates were examined across several exposures, mortality outcomes, and sensitivity analyses without formal adjustment for multiple comparisons. Isolated statistically non-null estimates may therefore have arisen by chance. Although the maternal-smoking–respiratory-mortality interaction was observed in several sensitivity analyses, these analyses involved overlapping participant samples and should not be regarded as independent replications. Confirmation in independent population-based cohorts and clinically adjudicated, ethnically diverse interstitial lung disease registries is warranted.

From a public health and epidemiologic perspective, these findings reinforce established efforts to prevent active smoking and exposure to secondhand smoke. The joint analyses also describe the particularly high mortality risk among participants with recorded IPF, although they do not support a general synergistic effect between IPF and smoking-related exposures. The maternal-smoking finding raises a hypothesis about early-life susceptibility to respiratory mortality that requires testing in other populations.

## 5 Conclusion

In conclusion, recorded IPF and several active and passive smoking measures were associated with higher mortality in this UK Biobank cohort, with the highest risks generally observed among participants with both factors. A positive additive interaction was found only between maternal smoking around birth and recorded IPF for respiratory mortality and was reproduced in several internal sensitivity analyses. Pack-years were approximately linearly associated with mortality among participants with recorded IPF, although these estimates were less precise. The maternal-smoking interaction warrants external confirmation, while the broader findings support continued attention to active and passive tobacco-smoke exposure across the life course.

## Data Availability

The data analyzed in this study are subject to the following licenses/restrictions: the data are available from the UK Biobank upon reasonable request and subject to UK Biobank access policies. This research was conducted under UK Biobank application No. 91536. Public sharing of individual-level data is restricted by UK Biobank policy. Only Qiyun Tu, Xinyue Zhang, Yuxin Zou, Huaqi Guo, and Baopeng Liu had direct access to the individual-level data used in this study. Requests to access these datasets should be directed to UK Biobank at https://www.ukbiobank.ac.uk/.
