# Supplementary material for: Smoking exposures and mortality risk in individuals with idiopathic pulmonary fibrosis: a UK Biobank cohort study
Source: Front Public Health. 2026 Sep 16;14:1884390. doi: 10.3389/fpubh.2026.1884390 (PMC13624122; doi:10.3389/fpubh.2026.1884390)
Supplement: Supplementary file 1 [file Table_1.DOCX]

Supplementary Material

**Supplementary Table S1. UK Biobank questionnaire fields used in the ascertainment of recorded IPF**

| **Medical condition** | **Self-reported UK Biobank field code** |
| --- | --- |
| Idiopathic pulmonary fibrosis (IPF) | 22155, 22135, 22175 |

Abbreviations: IPF, idiopathic pulmonary fibrosis.

**Supplementary Table S2. ICD-10 definitions for cause-specific mortality categories**

| **Diseases** | **ICD-10 codes** |
| --- | --- |
| Cardiovascular disease (CVD) mortality | I00–I09, I11, I13, I20–I51, I60–I69 |
| Cancer mortality | C00–C97 |
| Respiratory mortality | J00–J99 |

Abbreviations: CVD, cardiovascular disease. ICD-10 codes were used to define cardiovascular disease, cancer, and respiratory mortality as shown in the table.

**Supplementary Table S3. Medical conditions used as exclusion criteria to identify IPF cases**

| **Medical condition** | **UK Biobank field code** | **UK Biobank ICD-10 code** |
| --- | --- | --- |
| Rheumatoid arthritis and rheumatoid lung | 131849,131851 | J99.0, M05.10, M05.17, M05.18, M05.19, M06.00, M06.01, M06.02, M06.03, M06.04, M06.05, M06.06, M06.07, M06.08, M06.09, M06.10, M06.11, M06.16, M06.19, M06.21, M06.22, M06.25, M06.26, M06.27, M06.30, M06.32, M06.33, M06.34, M06.36, M06.37, M06.38, M06.39, M06.40, M06.41, M06.43, M06.44, M06.45, M06.46, M06.47, M06.49, M06.80, M06.81, M06.82, M06.84, M06.85, M06.86, M06.87, M06.88, M06.89, M06.90, M06.91, M06.92, M06.93, M06.94, M06.95, M06.96, M06.97, M06.98, M06.99 |
| Polyarteritis nodosa | 131891 | M30.0, M30.1, M30.2, M30.3, M30.8 |
| Other necrotizing vasculitis | 131893 | M31.0, M31.1, M31.2, M31.3, M31.4, M31.5, M31.6, M31.7, M31.8, M31.9 |
| Systemic lupus erythematosus | 131895 | M32.0, M32.1, M32.8, M32.9, M32.90 |
| Dermatomyositis polymyositis | 131897 | M33.0, M33.1, M33.2, M33.9 |
| Systemic sclerosis | 131831, 131899 | L94.0, L94.1, L94.2, L94.3, L94.4, L94.5, L94.8, L94.9 |
| Sjogren syndrome | 131901 | M35.0 |
| Other connective tissue diseases | 131901 | M35.1, M35.2, M35.3, M35.4, M35.5, M35.7, M35.8, M35.9, M35.99 |
| Ankylosing spondylitis | 131913 | M45, M45.X0, M45.X1, M45.X2, M45.X3, M45.X4, M45.X5, M45.X6, M45.X7, M45.X8, M45.X9 |
| Sarcoidosis | 130687 | D86.0, D86.1, D86.2, D86.3, D86.8, D86.9 |
| Neurofibromatosis |  | Q85.0 |
| Tuberous sclerosis |  | Q85.1 |
| Coal worker’s disease | 131501 | J60 |
| Asbestos | 131503 | J61,J92.0 |
| Pneumoconiosis | 131505, 131507, 131509, 131511, 131513 | J62.8, J63.4, J63.5, J63.8, J64, J66.0, J66.8 |
| Hypersensitivity pneumonitis due to organic dust | 131515 | J67.0, J67.2, J67.4, J67.6, J67.7, J67.8, J67.9 |
| Chronic respiratory conditions due to fumes or vapors | 131517 | J68.0, J68.2, J68.4, J68.8, J68.9 |
| Respiratory conditions due to other specified external agents | 131521 | J70.0, J70.1, J70.2, J70.3, J70.4, J70.8, J70.9 |
| Pulmonary eosinophilia | 131527 | J82 |
| Lung involvement in other diseases classified elsewhere | 131551 | J99.1, J99.8 |
| Extrinsic allergic alveolitis | 22156, 22136 |  |
| Lipidosis | 130809 | E75.0, E75.2, E75.3, E75.4, E75.5, E75.6 |
| Amyloidosis | 130825 | E85.0, E85.1, E85.2, E85.3, E85.4, E85.8, E85.9 |
| Other metabolic disorders | 130831 | E88.0, E88.1, E88.2, E88.3, E88.8, E88.9 |

Abbreviations: IPF, idiopathic pulmonary fibrosis. UK Biobank field codes and ICD-10 codes were used to identify exclusion conditions, as shown in the table.

**Supplementary Table S4. Summary of 6 SNPs used in the study of Oldham et al., 2023**

| **Chromosome** | **rsID** | **Position** | **Locus** | **Effect allele** | **EAF** | **HR (95% CI)** |
| --- | --- | --- | --- | --- | --- | --- |
| 15 | rs59424629 | 40720542 | IVD | T | 0.573 | 1.12 (1.01–1.23) |
| 11 | rs35705950 | 1241221 | MUC5B | T | 0.321 | 0.78 (0.64–0.94) |
| 2 | rs184498750 | 84291167 | SUCLG1 | T | 0.012 | 2.05 (1.79–4.18) |
| 15 | rs60514164 | 76081200 | UBE2Q2 | T | 0.077 | 1.42 (1.04–1.93) |
| 15 | rs35647788 | 101914234 | PCSK6 | T | 0.008 | 3.12 (1.37–7.11) |
| 19 | rs3893252 | 1412985 | DAZAP1 | T | 0.015 | 2.09 (1.05–4.15) |

Abbreviations: SNP, single nucleotide polymorphism; EAF, effect allele frequency; HR, hazard ratio; CI, confidence interval.

**Supplementary Table S5. Data field and definition of the included covariates**

| **Covariates** | **Data field** | **Question and description** |
| --- | --- | --- |
| Age | 21022 | Age at first recruitment |
| Sex | 31 | Self-reported sex during the initial Assessment Centre visit |
| Ethnicity | 21000 | Self-reported ethnic background during the initial Assessment Centre visit |
| Socioeconomic status | 22189 | Townsend deprivation index, which could reflect socioeconomic status, was calculated immediately prior to participant joining UK Biobank. Based on the preceding national census output areas. Each participant is assigned a score corresponding to the output area in which their postcode is located. A higher score means greater socioeconomic deprivation. Q1 indicates the least deprived quartile and Q4 the most deprived quartile of the Townsend deprivation index. |
| BMI | 21001 | BMI value here is constructed from height and weight measured during the initial Assessment Centre visit |
| Household income before tax per year (£) | 738 | Touchscreen question "What is the average total income before tax received by your HOUSEHOLD per year?" |
| Study center region | 54 | The UK Biobank assessment center at which participant consented |
| Education group | 6138 | Touchscreen question "Which of the following qualifications do you have?" |
| Smoking status | 20116 | Smoking status was derived from UK Biobank field 20116, which is a summary for many questions about smoking |
| Alcohol consumption | 20117, 1558, 1568, 1578, 1588, 1598, 1608, 5364, 4407, 4418, 4429, 4440, 4451, 4462 | UK Biobank Touchscreen questionnaire at baseline; US Dietary guidelines for Americans 2015-2020 of up to 1 drink/day for women and up to 2 drinks/day for men. To calculate drink-equivalents as per guidelines, multiply the volume in ounces by the alcohol content in percent and divide by 0.6 ounces of alcohol per drink-equivalent; then convert to grams: 1 drink-equivalent described as containing 14g of pure alcohol. 125ml wine=0.85 drink-equivalents, 4% ABV pint beer = 1.28 drink-equivalents, 25ml spirits=0.57 drink-equivalents, 50ml fortified wine= 0.56 drink-equivalents, Moderate consumption: women: >0 and ≤14g/day men: >0 and ≤28g/day |
| Healthy diet | 1309, 1319, 1289, 1299, 1329, 1339, 1349, 1369, 1379, 1389, 1438, 1448, 1458, 1468 | UK Biobank Food Frequency Questionnaire at baseline; At least 4 of the following 7 food groups: 1. Fruits: ≥ 3 servings/day 2. Vegetables: ≥ 3 servings/day 3. Fish: ≥ 2 servings/week 4. Processed meats: ≤ 1 serving/week 5. Unprocessed red meats: ≤ 1.5 servings/week 6. Whole grains: ≥ 3 servings/day 7. Refined grains: ≤ 1.5 servings/day |
| Regular physical activity | 884, 894, 904, 914 | UK Biobank Touchscreen questionnaire at baseline; ≥150 minutes moderate activity per week OR ≥ 75 minutes vigorous activity per week OR equivalent combination OR moderate physical activity at least 5 days a week or vigorous activity once a week |
| Adult SHS exposure | 1269,1279 | UK Biobank touchscreen question "At home, about how many hours per WEEK are you exposed to other people's tobacco smoke?"  UK Biobank touchscreen question "Outside of your home, about how many hours per WEEK are you exposed to other people's tobacco smoke?" |
| Passive SHS exposure | 1259,1269,1279,1787 | A composite passive SHS exposure variable (passive SHS exposure) was created to reflect overall passive SHS exposure status. Participants were considered exposed if they had any of the following: adult SHS exposure, household SHS exposure, or maternal smoking exposure |
| Maternal smoking exposure | 1787 | UK Biobank touchscreen question "Did your mother smoke regularly around the time when you were born?" |

Abbreviations: BMI, body mass index; ABV, alcohol by volume; SHS, secondhand smoke. Covariates were derived from the baseline UK Biobank assessment using the corresponding field IDs shown in the table.

**Supplementary Table S6. Baseline characteristics of participants included in the analytical cohort and those excluded because of missing exposure or covariate data**

| **Characteristics** | **Final included** | **Excluded due to missing data** | **P value** | **SMD** |
| --- | --- | --- | --- | --- |
|  | **(n = 270,644)** | **(n = 131,896)** |  |  |
| **Age, mean (SD)** | 56.58 (8.07) | 57.09 (8.05) | <0.001 | 0.064 |
| **BMI, mean (SD)** | 27.42 (4.75) | 27.63 (4.91) | <0.001 | 0.045 |
| Missing, n (%) | 0 (0.0%) | 1617 (1.2%) | <0.001 | 0.158 |
| **Sex, n (%)** |  |  | <0.001 | 0.043 |
| Female | 148606 (54.9%) | 69603 (52.8%) |  |  |
| Male | 122038 (45.1%) | 62293 (47.2%) |  |  |
| **Ethnicity, n (%)** |  |  | <0.001 | 0.112 |
| Non-white | 11741 (4.3%) | 8982 (6.8%) |  |  |
| White | 258903 (95.7%) | 121025 (91.8%) |  |  |
| Missing | 0 (0.0%) | 1889 (1.4%) | <0.001 | 0.17 |
| **Household income, n (%)** |  |  | <0.001 | 0.345 |
| 18000-30999 | 60071 (22.2%) | 28875 (21.9%) |  |  |
| 31000-51999 | 64555 (23.9%) | 24922 (18.9%) |  |  |
| 52000-100000 | 52067 (19.2%) | 15788 (12.0%) |  |  |
| Greater than 100000 | 13702 (5.1%) | 3309 (2.5%) |  |  |
| Less than 18000 | 47580 (17.6%) | 32201 (24.4%) |  |  |
| Unknown | 32669 (12.1%) | 24918 (18.9%) |  |  |
| Missing | 0 (0.0%) | 1883 (1.4%) | <0.001 | 0.17 |
| **Education level, n (%)** |  |  | <0.001 | 0.209 |
| Any school degree | 103092 (38.1%) | 49672 (37.7%) |  |  |
| College or University degree | 97178 (35.9%) | 32973 (25.0%) |  |  |
| Vocational qualification or other | 29502 (10.9%) | 16067 (12.2%) |  |  |
| None of the above | 0 (0.0%) | 406 (0.3%) |  |  |
| Missing | 40872 (15.1%) | 32778 (24.9%) | <0.001 | 0.246 |
| **Townsend deprivation, n (%)** |  |  | <0.001 | 0.26 |
| Q1 | 73426 (27.1%) | 27183 (20.6%) |  |  |
| Q2 | 71454 (26.4%) | 29465 (22.3%) |  |  |
| Q3 | 67912 (25.1%) | 32627 (24.7%) |  |  |
| Q4 | 57852 (21.4%) | 42134 (31.9%) |  |  |
| Missing | 0 (0.0%) | 487 (0.4%) | <0.001 | 0.086 |
| **Physical activity, n (%)** |  |  | <0.001 | 0.036 |
| Insufficient | 44803 (16.6%) | 21958 (16.6%) |  |  |
| Sufficient | 213473 (78.9%) | 95298 (72.3%) |  |  |
| Missing | 12368 (4.6%) | 14640 (11.1%) | <0.001 | 0.245 |
| **Alcohol consumption, n (%)** |  |  | <0.001 | 0.133 |
| Excessive drinking | 62745 (23.2%) | 35569 (27.0%) |  |  |
| Moderate drinking | 120052 (44.4%) | 48801 (37.0%) |  |  |
| Non-drinker | 87847 (32.5%) | 40555 (30.7%) |  |  |
| Missing | 0 (0.0%) | 6971 (5.3%) | <0.001 | 0.334 |
| **Diet quality, n (%)** |  |  | <0.001 | 0.093 |
| Healthy | 149596 (55.3%) | 60003 (45.5%) |  |  |
| Unhealthy | 121048 (44.7%) | 58443 (44.3%) |  |  |
| Missing | 0 (0.0%) | 13450 (10.2%) | <0.001 | 0.477 |
| **IPF status, n (%)** |  |  | <0.001 | 0.032 |
| Non-IPF | 269313 (99.5%) | 130920 (99.3%) |  |  |
| IPF | 1331 (0.5%) | 976 (0.7%) |  |  |
| **Smoking status, n (%)** |  |  | <0.001 | 0.725 |
| Current | 7684 (2.8%) | 35212 (26.7%) |  |  |
| Former | 101930 (37.7%) | 38730 (29.4%) |  |  |
| Never | 161030 (59.5%) | 55893 (42.4%) |  |  |
| Missing | 0 (0.0%) | 2061 (1.6%) | <0.001 | 0.178 |
| **Maternal smoking exposure, n (%)** |  |  | <0.001 | 0.036 |
| No | 191448 (70.7%) | 52337 (39.7%) |  |  |
| Yes | 79196 (29.3%) | 23405 (17.7%) |  |  |
| Missing | 0 (0.0%) | 56154 (42.6%) | <0.001 | 1.218 |
| **Adult SHS exposure, n (%)** |  |  | <0.001 | 0.062 |
| No | 210708 (77.9%) | 51134 (38.8%) |  |  |
| Yes | 59936 (22.1%) | 16829 (12.8%) |  |  |
| Missing | 0 (0.0%) | 63933 (48.5%) | <0.001 | 1.372 |

Values are presented as mean (standard deviation) for continuous variables and number (percentage) for categorical variables. Percentages were calculated using the total number of participants in each group. Missing indicates that no valid value was available for the corresponding variable after data cleaning. Participants were classified as excluded because of missing data if at least one required exposure or covariate was missing; therefore, they could still have observed values for other characteristics. Unknown denotes an explicitly coded response category, such as “do not know” or “prefer not to answer,” that was retained as a categorical level and was not treated as missing. For the main characteristic rows, P values and standardized mean differences were calculated after excluding Missing values, while retaining Unknown as a categorical level. Continuous variables were compared using Welch’s t test, and categorical variables were compared using Pearson’s chi-square test. For the Missing rows, P values were calculated using two-sample tests of proportions, and standardized mean differences compare the proportions of missing values between groups. An absolute standardized mean difference of 0.10 or greater was considered indicative of potential imbalance. BMI, body mass index; IPF, idiopathic pulmonary fibrosis; SHS, secondhand smoke; SMD, standardized mean difference.

**Supplementary Table S7. Independent associations of smoking-related exposures and IPF with cause-specific mortality during follow-up**

| **Exposure** | **Person-years**  **(PYs)** | **Cases/N** | **Incidence Rate**  **(95% CI)** | **HR (95% CI)** | | |
| --- | --- | --- | --- | --- | --- | --- |
|  |  |  |  | **Model 1** | **Model 2** | **Model 3** |
| **CVD mortality** |  |  |  |  |  |  |
| **IPF** |  |  |  |  |  |  |
| No | 3629780.01 | 3643/269313 | 1.004 (0.971–1.036) | 1.000 (ref) | 1.000 (ref) | 1.000 (ref) |
| Yes | 15583.11 | 87/1331 | 5.583 (4.410–6.756) | 6.015 (4.863–7.441)*** | 2.499 (2.018–3.094)*** | 2.457 (1.982–3.046)*** |
| **Smoking status** |  |  |  |  |  |  |
| Never | 2182639.77 | 1679/161030 | 0.769 (0.732–0.806) | 1.000 (ref) | 1.000 (ref) | 1.000 (ref) |
| Former | 1359574.98 | 1913/101930 | 1.407 (1.344–1.470) | 1.842 (1.725–1.967)*** | 1.249 (1.167–1.337)*** | 1.250 (1.167–1.337)*** |
| Current | 103148.37 | 138/7684 | 1.338 (1.115–1.561) | 1.745 (1.467–2.075)*** | 1.606 (1.348–1.913)*** | 1.605 (1.347–1.912)*** |
| **Adult SHS exposure** |  |  |  |  |  |  |
| Never | 2839213.94 | 2703/210708 | 0.952 (0.916–0.988) | 1.000 (ref) | 1.000 (ref) | 1.000 (ref) |
| Ever | 806149.18 | 1027/59936 | 1.274 (1.196–1.352) | 1.336 (1.243–1.435)*** | 1.173 (1.090–1.262)*** | 1.172 (1.089–1.262)*** |
| **Maternal smoking exposure** |  |  |  |  |  |  |
| Never | 2577889.42 | 2604/191448 | 1.010 (0.971–1.049) | 1.000 (ref) | 1.000 (ref) | 1.000 (ref) |
| Ever | 1067473.70 | 1126/79196 | 1.055 (0.993–1.116) | 1.042 (0.972–1.117) | 1.085 (1.010–1.165)* | 1.084 (1.010–1.164)* |
| **Passive SHS exposure** |  |  |  |  |  |  |
| Never | 1930660.54 | 1842/143325 | 0.954 (0.911–0.998) | 1.000 (ref) | 1.000 (ref) | 1.000 (ref) |
| Ever | 1714702.58 | 1888/127319 | 1.101 (1.051–1.151) | 1.152 (1.080–1.228)*** | 1.129 (1.057–1.205)*** | 1.128 (1.056–1.205)*** |
| **Pack years** |  |  |  |  |  |  |
| 0 | 2187982.75 | 1689/161443 | 0.772 (0.735–0.809) | 1.000 (ref) | 1.000 (ref) | 1.000 (ref) |
| 1–10 | 269806.52 | 181/19929 | 0.671 (0.573–0.769) | 0.870 (0.747–1.015) | 0.835 (0.716–0.974)* | 0.836 (0.717–0.975)* |
| 11–20 | 259054.69 | 281/19304 | 1.085 (0.958–1.212) | 1.412 (1.244–1.602)*** | 1.053 (0.927–1.196) | 1.053 (0.927–1.196) |
| 21–30 | 164546.36 | 277/12346 | 1.683 (1.485–1.882) | 2.194 (1.932–2.491)*** | 1.372 (1.206–1.560)*** | 1.371 (1.205–1.560)*** |
| >30 | 198646.20 | 627/15535 | 3.156 (2.909–3.403) | 4.183 (3.817–4.585)*** | 1.791 (1.625–1.973)*** | 1.791 (1.625–1.974)*** |
| Unknown | 565326.60 | 675/42087 | 1.194 (1.104–1.284) | 1.554 (1.421–1.699)*** | 1.224 (1.118–1.340)*** | 1.225 (1.119–1.341)*** |
| **Cancer mortality** |  |  |  |  |  |  |
| **IPF** |  |  |  |  |  |  |
| No | 3629780.01 | 10756/269313 | 2.963 (2.907–3.019) | 1.000 (ref) | 1.000 (ref) | 1.000 (ref) |
| Yes | 15583.11 | 156/1331 | 10.011 (8.440–11.582) | 3.523 (3.008–4.127)*** | 2.038 (1.739–2.389)*** | 2.026 (1.727–2.375)*** |
| **Smoking status** |  |  |  |  |  |  |
| Never | 2182639.77 | 5341/161030 | 2.447 (2.381–2.513) | 1.000 (ref) | 1.000 (ref) | 1.000 (ref) |
| Former | 1359574.98 | 5235/101930 | 3.850 (3.746–3.955) | 1.580 (1.521–1.641)*** | 1.280 (1.230–1.332)*** | 1.280 (1.230–1.331)*** |
| Current | 103148.37 | 336/7684 | 3.257 (2.909–3.606) | 1.334 (1.195–1.489)*** | 1.418 (1.269–1.585)*** | 1.418 (1.269–1.585)*** |
| **Adult SHS exposure** |  |  |  |  |  |  |
| Never | 2839213.94 | 8374/210708 | 2.949 (2.886–3.013) | 1.000 (ref) | 1.000 (ref) | 1.000 (ref) |
| Ever | 806149.18 | 2538/59936 | 3.148 (3.026–3.271) | 1.067 (1.021–1.116)** | 1.071 (1.024–1.121)** | 1.072 (1.024–1.121)** |
| **Maternal smoking exposure** |  |  |  |  |  |  |
| Never | 2577889.42 | 7755/191448 | 3.008 (2.941–3.075) | 1.000 (ref) | 1.000 (ref) | 1.000 (ref) |
| Ever | 1067473.70 | 3157/79196 | 2.957 (2.854–3.061) | 0.982 (0.942–1.024) | 1.021 (0.979–1.065) | 1.020 (0.979–1.064) |
| **Passive SHS exposure** |  |  |  |  |  |  |
| Never | 1930660.54 | 5738/143325 | 2.972 (2.895–3.049) | 1.000 (ref) | 1.000 (ref) | 1.000 (ref) |
| Ever | 1714702.58 | 5174/127319 | 3.017 (2.935–3.100) | 1.015 (0.977–1.054) | 1.056 (1.017–1.098)** | 1.056 (1.017–1.098)** |
| **Pack years** |  |  |  |  |  |  |
| 0 | 2187982.75 | 5374/161443 | 2.456 (2.390–2.522) | 1.000 (ref) | 1.000 (ref) | 1.000 (ref) |
| 1–10 | 269806.52 | 739/19929 | 2.739 (2.542–2.936) | 1.116 (1.033–1.205)** | 1.071 (0.992–1.157) | 1.071 (0.992–1.157) |
| 11–20 | 259054.69 | 889/19304 | 3.432 (3.206–3.657) | 1.401 (1.305–1.504)*** | 1.203 (1.120–1.293)*** | 1.203 (1.119–1.292)*** |
| 21–30 | 164546.36 | 672/12346 | 4.084 (3.775–4.393) | 1.669 (1.540–1.808)*** | 1.311 (1.208–1.422)*** | 1.311 (1.208–1.422)*** |
| >30 | 198646.20 | 1530/15535 | 7.702 (7.316–8.088) | 3.178 (3.002–3.364)*** | 2.044 (1.924–2.171)*** | 2.043 (1.923–2.170)*** |
| Unknown | 565326.60 | 1708/42087 | 3.021 (2.878–3.165) | 1.233 (1.168–1.302)*** | 1.095 (1.036–1.157)** | 1.095 (1.036–1.157)** |
| **Respiratory mortality** |  |  |  |  |  |  |
| **IPF** |  |  |  |  |  |  |
| No | 3629780.01 | 723/269313 | 0.199 (0.185–0.214) | 1.000 (ref) | 1.000 (ref) | 1.000 (ref) |
| Yes | 15583.11 | 353/1331 | 22.653 (20.290–25.016) | 124.596 (109.667–141.557)*** | 53.480 (46.737–61.196)*** | 52.482 (45.658–60.326)*** |
| **Smoking status** |  |  |  |  |  |  |
| Never | 2182639.77 | 353/161030 | 0.162 (0.145–0.179) | 1.000 (ref) | 1.000 (ref) | 1.000 (ref) |
| Former | 1359574.98 | 680/101930 | 0.500 (0.463–0.538) | 3.116 (2.740–3.544)*** | 2.083 (1.824–2.379)*** | 2.078 (1.819–2.374)*** |
| Current | 103148.37 | 43/7684 | 0.417 (0.292–0.541) | 2.588 (1.885–3.551)*** | 2.459 (1.786–3.384)*** | 2.440 (1.772–3.358)*** |
| **Adult SHS exposure** |  |  |  |  |  |  |
| Never | 2839213.94 | 748/210708 | 0.263 (0.245–0.282) | 1.000 (ref) | 1.000 (ref) | 1.000 (ref) |
| Ever | 806149.18 | 328/59936 | 0.407 (0.363–0.451) | 1.543 (1.355–1.757)*** | 1.360 (1.191–1.553)*** | 1.361 (1.192–1.554)*** |
| **Maternal smoking exposure** |  |  |  |  |  |  |
| Never | 2577889.42 | 718/191448 | 0.279 (0.258–0.299) | 1.000 (ref) | 1.000 (ref) | 1.000 (ref) |
| Ever | 1067473.70 | 358/79196 | 0.335 (0.301–0.370) | 1.202 (1.059–1.364)** | 1.275 (1.121–1.450)*** | 1.268 (1.114–1.442)*** |
| **Passive SHS exposure** |  |  |  |  |  |  |
| Never | 1930660.54 | 476/143325 | 0.247 (0.224–0.269) | 1.000 (ref) | 1.000 (ref) | 1.000 (ref) |
| Ever | 1714702.58 | 600/127319 | 0.350 (0.322–0.378) | 1.417 (1.257–1.598)*** | 1.417 (1.253–1.602)*** | 1.412 (1.249–1.596)*** |
| **Pack years** |  |  |  |  |  |  |
| 0 | 2187982.75 | 362/161443 | 0.165 (0.148–0.182) | 1.000 (ref) | 1.000 (ref) | 1.000 (ref) |
| 1–10 | 269806.52 | 48/19929 | 0.178 (0.128–0.228) | 1.077 (0.797–1.455) | 1.044 (0.772–1.413) | 1.042 (0.770–1.409) |
| 11–20 | 259054.69 | 86/19304 | 0.332 (0.262–0.402) | 2.016 (1.594–2.551)*** | 1.536 (1.211–1.948)*** | 1.538 (1.213–1.951)*** |
| 21–30 | 164546.36 | 92/12346 | 0.559 (0.445–0.673) | 3.402 (2.706–4.277)*** | 2.173 (1.722–2.742)*** | 2.158 (1.711–2.724)*** |
| >30 | 198646.20 | 324/15535 | 1.631 (1.453–1.809) | 10.113 (8.705–11.749)*** | 4.346 (3.696–5.110)*** | 4.320 (3.674–5.080)*** |
| Unknown | 565326.60 | 164/42087 | 0.290 (0.246–0.334) | 1.762 (1.465–2.119)*** | 1.425 (1.183–1.717)*** | 1.424 (1.182–1.716)*** |

Abbreviations: PYs, person-years; Cases/N, number of deaths/total number of participants; CI, confidence interval; HR, hazard ratio; CVD, cardiovascular disease; BMI, body mass index; PA, physical activity; SHS, secondhand smoke; SNP, single nucleotide polymorphism. Incidence rates are presented as events per 1,000 person-years, and “ref” indicates the reference category.

Hazard ratios (HRs) and 95% CIs were estimated using Cox proportional hazards regression. Model 1 was unadjusted. Model 2 was adjusted for sex, age at recruitment, body mass index (BMI), household income, educational attainment, ethnicity, Townsend deprivation index quartiles, physical activity (PA), alcohol consumption, and diet. Model 3 was additionally adjusted for candidate SNPs. Pack-years were categorized as 0, 1–10, 11–20, 21–30, and >30; “Unknown” indicates missing pack-years information. Passive SHS exposure was defined as a composite measure of secondhand smoke exposure. Statistical significance is indicated as *P < 0.05, **P < 0.01, and ***P < 0.001.

**Supplementary Table S8. Stratified analysis of associations between smoking-related exposures and all-cause mortality during follow-up according to IPF status**

| **Exposure** | **Non IPF** | | | | **IPF** | | | |
| --- | --- | --- | --- | --- | --- | --- | --- | --- |
|  | **Cases/N** | **HR (95% CI)** | | | **Cases/N** | **HR (95% CI)** | | |
|  |  | **model 1** | **model 2** | **model 3** |  | **model 1** | **model 2** | **model 3** |
| Smoking status |  |  |  |  |  |  |  |  |
| Non-current | 19627/261684 | 1.000 (ref) | 1.000 (ref) | 1.000 (ref) | 651/1276 | 1.000 (ref) | 1.000 (ref) | 1.000 (ref) |
| Current | 650/7629 | 1.140 (1.055–1.233)*** | 1.288 (1.191–1.394)*** | 1.288 (1.191–1.394)*** | 31/55 | 1.060 (0.739–1.520) | 1.105 (0.767–1.590) | 1.093 (0.758–1.576) |
| Adult SHS exposure |  |  |  |  |  |  |  |  |
| Never | 15231/209758 | 1.000 (ref) | 1.000 (ref) | 1.000 (ref) | 471/950 | 1.000 (ref) | 1.000 (ref) | 1.000 (ref) |
| Ever | 5046/59555 | 1.167 (1.131–1.205)*** | 1.118 (1.082–1.155)*** | 1.118 (1.082–1.155)*** | 211/381 | 1.188 (1.010–1.398)* | 1.200 (1.016–1.418)* | 1.213 (1.026–1.435)* |
| Maternal smoking exposure |  |  |  |  |  |  |  |  |
| Never | 14339/190553 | 1.000 (ref) | 1.000 (ref) | 1.000 (ref) | 461/895 | 1.000 (ref) | 1.000 (ref) | 1.000 (ref) |
| Ever | 5938/78760 | 0.999 (0.969–1.030) | 1.041 (1.010–1.074)** | 1.041 (1.010–1.074)* | 221/436 | 0.996 (0.848–1.169) | 1.089 (0.924–1.283) | 1.068 (0.905–1.260) |
| Passive SHS exposure |  |  |  |  |  |  |  |  |
| Never | 10417/142714 | 1.000 (ref) | 1.000 (ref) | 1.000 (ref) | 308/611 | 1.000 (ref) | 1.000 (ref) | 1.000 (ref) |
| Ever | 9860/126599 | 1.066 (1.037–1.095)*** | 1.090 (1.059–1.121)*** | 1.090 (1.059–1.121)*** | 374/720 | 1.071 (0.921–1.245) | 1.138 (0.974–1.330) | 1.135 (0.971–1.327) |

Abbreviations: HR, hazard ratio; CI, confidence interval; SHS, secondhand smoke; “ref” indicates the reference category.

Hazard ratios (HRs) and 95% confidence intervals (CIs) were estimated using Cox proportional hazards regression. Model 1 was unadjusted. Model 2 was adjusted for sex, age at recruitment, body mass index (BMI), household income, educational attainment, ethnicity, Townsend deprivation index quartiles, physical activity (PA), alcohol consumption, and diet. Model 3 was additionally adjusted for candidate SNPs. For smoking status, the exposed category was current smoking and the reference category was non-current smoking, which included never and former smokers. For passive exposures, exposed participants were compared with unexposed participants. Statistical significance is indicated as *P < 0.05, **P < 0.01, and ***P < 0.001.

**Supplementary Table S9. Joint associations of smoking-related exposures and IPF with all-cause and cause-specific mortality during follow-up**

| **Joint variables** | **Cases/N** | **HR (95% CI)** | | |
| --- | --- | --- | --- | --- |
|  |  | **Model 1** | **Model 2** | **Model 3** |
| **All-cause mortality** |  |  |  |  |
| Smoking status |  |  |  |  |
| Non-current * No IPF | 19627/261684 | 1.000 (ref) | 1.000 (ref) | 1.000 (ref) |
| Non-current * IPF | 651/1276 | 8.404 (7.772–9.086)*** | 4.277 (3.953–4.628)*** | 4.258 (3.933–4.609)*** |
| Current * No IPF | 650/7629 | 1.140 (1.055–1.233)*** | 1.288 (1.191–1.394)*** | 1.288 (1.191–1.394)*** |
| Current * IPF | 31/55 | 8.977 (6.312–12.769)*** | 4.981 (3.501–7.087)*** | 4.964 (3.489–7.062)*** |
| Adult SHS exposure |  |  |  |  |
| Never * No IPF | 15231/209758 | 1.000 (ref) | 1.000 (ref) | 1.000 (ref) |
| Never * IPF | 471/950 | 8.296 (7.569–9.093)*** | 4.271 (3.894–4.683)*** | 4.250 (3.874–4.663)*** |
| Ever * No IPF | 5046/59555 | 1.167 (1.131–1.205)*** | 1.120 (1.084–1.157)*** | 1.120 (1.084–1.157)*** |
| Ever * IPF | 211/381 | 9.781 (8.539–11.205)*** | 4.728 (4.124–5.420)*** | 4.709 (4.107–5.400)*** |
| Maternal smoking exposure |  |  |  |  |
| Never * No IPF | 14339/190553 | 1.000 (ref) | 1.000 (ref) | 1.000 (ref) |
| Never * IPF | 461/895 | 8.413 (7.668–9.231)*** | 4.174 (3.802–4.583)*** | 4.157 (3.785–4.565)*** |
| Ever * No IPF | 5938/78760 | 0.999 (0.969–1.030) | 1.043 (1.011–1.075)** | 1.042 (1.011–1.075)** |
| Ever * IPF | 221/436 | 8.349 (7.310–9.536)*** | 4.682 (4.098–5.350)*** | 4.657 (4.075–5.324)*** |
| Passive SHS exposure |  |  |  |  |
| Never * No IPF | 10417/142714 | 1.000 (ref) | 1.000 (ref) | 1.000 (ref) |
| Never * IPF | 308/611 | 8.369 (7.472–9.373)*** | 4.226 (3.771–4.735)*** | 4.208 (3.754–4.716)*** |
| Ever * No IPF | 9860/126599 | 1.066 (1.037–1.095)*** | 1.091 (1.061–1.123)*** | 1.091 (1.061–1.122)*** |
| Ever * IPF | 374/720 | 8.903 (8.030–9.871)*** | 4.676 (4.215–5.188)*** | 4.655 (4.194–5.167)*** |
| **CVD mortality** |  |  |  |  |
| Smoking status |  |  |  |  |
| Non-current * No IPF | 3507/261684 | 1.000 (ref) | 1.000 (ref) | 1.000 (ref) |
| Non-current * IPF | 85/1276 | 6.197 (4.997–7.686)*** | 2.571 (2.071–3.193)*** | 2.528 (2.034–3.142)*** |
| Current * No IPF | 136/7629 | 1.335 (1.125–1.584)*** | 1.447 (1.218–1.718)*** | 1.446 (1.217–1.718)*** |
| Current * IPF | 2/55 | 3.265 (0.816–13.062) | 1.468 (0.367–5.875) | 1.455 (0.364–5.822) |
| Adult SHS exposure |  |  |  |  |
| Never * No IPF | 2639/209758 | 1.000 (ref) | 1.000 (ref) | 1.000 (ref) |
| Never * IPF | 64/950 | 6.565 (5.123–8.413)*** | 2.795 (2.179–3.585)*** | 2.748 (2.140–3.528)*** |
| Ever * No IPF | 1004/59555 | 1.339 (1.245–1.440)*** | 1.180 (1.096–1.271)*** | 1.180 (1.095–1.271)*** |
| Ever * IPF | 23/381 | 6.207 (4.117–9.357)*** | 2.221 (1.471–3.353)*** | 2.184 (1.446–3.299)*** |
| Maternal smoking exposure |  |  |  |  |
| Never * No IPF | 2541/190553 | 1.000 (ref) | 1.000 (ref) | 1.000 (ref) |
| Never * IPF | 63/895 | 6.547 (5.098–8.406)*** | 2.631 (2.047–3.382)*** | 2.589 (2.013–3.331)*** |
| Ever * No IPF | 1102/78760 | 1.046 (0.974–1.122) | 1.089 (1.013–1.170)* | 1.089 (1.013–1.170)* |
| Ever * IPF | 24/436 | 5.162 (3.454–7.717)*** | 2.400 (1.604–3.590)*** | 2.356 (1.573–3.527)*** |
| Passive SHS exposure |  |  |  |  |
| Never * No IPF | 1797/142714 | 1.000 (ref) | 1.000 (ref) | 1.000 (ref) |
| Never * IPF | 45/611 | 7.149 (5.318–9.611)*** | 2.989 (2.221–4.021)*** | 2.940 (2.184–3.958)*** |
| Ever * No IPF | 1846/126599 | 1.156 (1.083–1.233)*** | 1.135 (1.062–1.213)*** | 1.134 (1.062–1.212)*** |
| Ever * IPF | 42/720 | 5.848 (4.306–7.941)*** | 2.385 (1.754–3.244)*** | 2.344 (1.722–3.190)*** |
| **Cancer mortality** |  |  |  |  |
| Smoking status |  |  |  |  |
| Non-current * No IPF | 10430/261684 | 1.000 (ref) | 1.000 (ref) | 1.000 (ref) |
| Non-current * IPF | 146/1276 | 3.451 (2.931–4.064)*** | 1.996 (1.694–2.352)*** | 1.984 (1.683–2.339)*** |
| Current * No IPF | 326/7629 | 1.076 (0.964–1.202) | 1.238 (1.108–1.383)*** | 1.238 (1.109–1.384)*** |
| Current * IPF | 10/55 | 5.319 (2.861–9.888)*** | 3.375 (1.815–6.276)*** | 3.358 (1.806–6.245)*** |
| Adult SHS exposure |  |  |  |  |
| Never * No IPF | 8265/209758 | 1.000 (ref) | 1.000 (ref) | 1.000 (ref) |
| Never * IPF | 109/950 | 3.447 (2.854–4.164)*** | 2.005 (1.659–2.424)*** | 1.993 (1.648–2.410)*** |
| Ever * No IPF | 2491/59555 | 1.063 (1.016–1.111)** | 1.069 (1.021–1.119)** | 1.069 (1.021–1.119)** |
| Ever * IPF | 47/381 | 3.899 (2.927–5.194)*** | 2.246 (1.685–2.993)*** | 2.233 (1.675–2.977)*** |
| Maternal smoking exposure |  |  |  |  |
| Never * No IPF | 7649/190553 | 1.000 (ref) | 1.000 (ref) | 1.000 (ref) |
| Never * IPF | 106/895 | 3.531 (2.915–4.277)*** | 2.003 (1.653–2.428)*** | 1.992 (1.643–2.415)*** |
| Ever * No IPF | 3107/78760 | 0.981 (0.941–1.022) | 1.020 (0.977–1.064) | 1.019 (0.977–1.063) |
| Ever * IPF | 50/436 | 3.446 (2.609–4.551)*** | 2.156 (1.632–2.848)*** | 2.140 (1.619–2.828)*** |
| Passive SHS exposure |  |  |  |  |
| Never * No IPF | 5669/142714 | 1.000 (ref) | 1.000 (ref) | 1.000 (ref) |
| Never * IPF | 69/611 | 3.358 (2.649–4.258)*** | 1.924 (1.517–2.441)*** | 1.914 (1.508–2.429)*** |
| Ever * No IPF | 5087/126599 | 1.011 (0.973–1.050) | 1.054 (1.014–1.095)** | 1.053 (1.014–1.095)** |
| Ever * IPF | 87/720 | 3.701 (2.995–4.574)*** | 2.244 (1.814–2.775)*** | 2.228 (1.801–2.757)*** |
| **Respiratory mortality** |  |  |  |  |
| Smoking status |  |  |  |  |
| Non-current * No IPF | 698/261684 | 1.000 (ref) | 1.000 (ref) | 1.000 (ref) |
| Non-current * IPF | 335/1276 | 124.332 (109.107–141.682)*** | 53.320 (46.464–61.189)*** | 52.358 (45.417–60.361)*** |
| Current * No IPF | 25/7629 | 1.234 (0.828–1.839) | 1.371 (0.918–2.046) | 1.370 (0.918–2.045) |
| Current * IPF | 18/55 | 149.709 (93.759–239.048)*** | 70.106 (43.741–112.361)*** | 67.230 (41.905–107.861)*** |
| Adult SHS exposure |  |  |  |  |
| Never * No IPF | 508/209758 | 1.000 (ref) | 1.000 (ref) | 1.000 (ref) |
| Never * IPF | 240/950 | 129.337 (110.899–150.841)*** | 56.102 (47.829–65.806)*** | 54.962 (46.671–64.726)*** |
| Ever * No IPF | 215/59555 | 1.491 (1.271–1.748)*** | 1.361 (1.158–1.601)*** | 1.364 (1.160–1.604)*** |
| Ever * IPF | 113/381 | 161.363 (131.571–197.902)*** | 63.938 (51.648–79.152)*** | 63.136 (50.842–78.402)*** |
| Maternal smoking exposure |  |  |  |  |
| Never * No IPF | 488/190553 | 1.000 (ref) | 1.000 (ref) | 1.000 (ref) |
| Never * IPF | 230/895 | 126.052 (107.732–147.486)*** | 52.249 (44.376–61.519)*** | 51.139 (43.283–60.420)*** |
| Ever * No IPF | 235/78760 | 1.161 (0.994–1.356) | 1.242 (1.061–1.454)** | 1.239 (1.058–1.450)** |
| Ever * IPF | 123/436 | 139.627 (114.547–170.197)*** | 69.031 (56.320–84.611)*** | 68.346 (55.521–84.133)*** |
| Passive SHS exposure |  |  |  |  |
| Never * No IPF | 320/142714 | 1.000 (ref) | 1.000 (ref) | 1.000 (ref) |
| Never * IPF | 156/611 | 140.772 (116.226–170.502)*** | 59.162 (48.600–72.021)*** | 57.935 (47.456–70.729)*** |
| Ever * No IPF | 403/126599 | 1.417 (1.224–1.641)*** | 1.445 (1.245–1.677)*** | 1.445 (1.245–1.676)*** |
| Ever * IPF | 197/720 | 156.302 (130.847–186.710)*** | 70.156 (58.269–84.467)*** | 69.140 (57.190–83.588)*** |

Abbreviations: HR, hazard ratio; CI, confidence interval; CVD, cardiovascular disease; BMI, body mass index; PA, physical activity; SHS, secondhand smoke; SNP, single nucleotide polymorphism. Cases/N indicates the number of deaths/total number of participants, and “ref” indicates the reference category.

Hazard ratios (HRs) and 95% confidence intervals (CIs) were estimated using Cox proportional hazards regression. Model 1 was unadjusted. Model 2 was adjusted for sex, age at recruitment, body mass index (BMI), household income, educational attainment, ethnicity, Townsend deprivation index quartiles, physical activity (PA), alcohol consumption, and diet. Model 3 was additionally adjusted for candidate SNPs. For smoking status, the exposed category was current smoking and the reference category was non-current smoking, which included never and former smokers. For passive smoking exposures, exposed participants were compared with unexposed participants. Statistical significance is indicated as *P < 0.05, **P < 0.01, and ***P < 0.001.

**Supplementary Table S10. Multiplicative interactions between smoking-related exposures and IPF in relation to all-cause and cause-specific mortality during follow-up**

| **interaction** | **HR (95% CI)** | | | |
| --- | --- | --- | --- | --- |
|  | **All-cause mortality** | **CVD mortality** | **Cancer mortality** | **Respiratory mortality** |
| Smoking status | 0.905 (0.626–1.308) | 0.398 (0.097–1.635) | 1.367 (0.713–2.618) | 0.937 (0.503–1.745) |
| Adult SHS exposure | 0.989 (0.838–1.167) | 0.674 (0.416–1.092) | 1.048 (0.742–1.480) | 0.842 (0.639–1.110) |
| Maternal smoking exposure | 1.075 (0.913–1.266) | 0.836 (0.519–1.345) | 1.054 (0.751–1.479) | 1.079 (0.824–1.414) |
| Passive SHS exposure | 1.014 (0.870–1.182) | 0.703 (0.459–1.076) | 1.105 (0.804–1.519) | 0.826 (0.638–1.069) |

Abbreviations: HR, hazard ratio; CI, confidence interval; CVD, cardiovascular disease; BMI, body mass index; PA, physical activity; SHS, secondhand smoke; SNP, single nucleotide polymorphism.

Multiplicative interactions between smoking-related exposures and IPF in relation to all-cause and cause-specific mortality during follow-up were evaluated using the HR and 95% CI for the interaction term from Model 3. Model 3 was adjusted for sex, age at recruitment, body mass index (BMI), household income, educational attainment, ethnicity, Townsend deprivation index quartiles, physical activity (PA), alcohol consumption, diet, and candidate SNPs. For smoking status, the exposed category was current smoking and the reference category was non-current smoking, which included never and former smokers. For passive smoking exposures, exposed participants were compared with unexposed participants. Statistical significance is indicated as *P < 0.05, **P < 0.01, and ***P < 0.001.

**Supplementary Table S11. Independent associations of smoking-related exposures and IPF with all-cause and cause-specific mortality during follow-up among White participants**

| **Exposure** | **Person-years**  **(PYs)** | **Cases/N** | **Incidence Rate**  **(95% CI)** | **HR (95% CI)** | | |
| --- | --- | --- | --- | --- | --- | --- |
|  |  |  |  | **Model 1** | **Model 2** | **Model 3** |
| **All-cause mortality** |  |  |  |  |  |  |
| **IPF** |  |  |  |  |  |  |
| No | 3474564.86 | 19644/257612 | 5.654 (5.575–5.733) | 1.000 (ref) | 1.000 (ref) | 1.000 (ref) |
| Yes | 15160.84 | 662/1291 | 43.665 (40.339–46.991) | 8.268 (7.652–8.934)*** | 4.213 (3.896–4.555)*** | 4.189 (3.872–4.532)*** |
| **Smoking status** |  |  |  |  |  |  |
| Never | 2066624.91 | 9573/152291 | 4.632 (4.539–4.725) | 1.000 (ref) | 1.000 (ref) | 1.000 (ref) |
| Former | 1326249.48 | 10075/99402 | 7.597 (7.448–7.745) | 1.652 (1.606–1.698)*** | 1.260 (1.224–1.297)*** | 1.260 (1.224–1.297)*** |
| Current | 96851.32 | 658/7210 | 6.794 (6.275–7.313) | 1.471 (1.359–1.592)*** | 1.491 (1.377–1.614)*** | 1.491 (1.377–1.614)*** |
| **Adult SHS exposure** |  |  |  |  |  |  |
| Never | 2734739.20 | 15276/202832 | 5.586 (5.497–5.674) | 1.000 (ref) | 1.000 (ref) | 1.000 (ref) |
| Ever | 754986.50 | 5030/56071 | 6.662 (6.478–6.846) | 1.191 (1.153–1.229)*** | 1.129 (1.093–1.167)*** | 1.129 (1.093–1.167)*** |
| **Maternal smoking exposure** |  |  |  |  |  |  |
| Never | 2434502.90 | 14200/180625 | 5.833 (5.737–5.929) | 1.000 (ref) | 1.000 (ref) | 1.000 (ref) |
| Ever | 1055222.81 | 6106/78278 | 5.786 (5.641–5.932) | 0.991 (0.962–1.022) | 1.046 (1.015–1.078)** | 1.046 (1.014–1.078)** |
| **Passive SHS exposure** |  |  |  |  |  |  |
| Never | 1841425.43 | 10363/136594 | 5.628 (5.519–5.736) | 1.000 (ref) | 1.000 (ref) | 1.000 (ref) |
| Ever | 1648300.27 | 9943/122309 | 6.032 (5.914–6.151) | 1.071 (1.042–1.101)*** | 1.098 (1.068–1.129)*** | 1.098 (1.067–1.129)*** |
| **Pack years** |  |  |  |  |  |  |
| 0 | 2071671.18 | 9640/152680 | 4.653 (4.560–4.746) | 1.000 (ref) | 1.000 (ref) | 1.000 (ref) |
| 1–10 | 262502.31 | 1231/19385 | 4.689 (4.428–4.951) | 1.010 (0.952–1.072) | 0.972 (0.915–1.031) | 0.972 (0.916–1.031) |
| 11–20 | 252961.55 | 1626/18844 | 6.428 (6.115–6.740) | 1.388 (1.317–1.463)*** | 1.138 (1.079–1.200)*** | 1.138 (1.079–1.200)*** |
| 21–30 | 161013.58 | 1306/12076 | 8.111 (7.671–8.551) | 1.754 (1.656–1.858)*** | 1.272 (1.200–1.349)*** | 1.272 (1.200–1.349)*** |
| >30 | 195908.80 | 3111/15314 | 15.880 (15.322–16.438) | 3.487 (3.349–3.630)*** | 1.928 (1.847–2.013)*** | 1.928 (1.847–2.013)*** |
| Unknown | 545668.28 | 3392/40604 | 6.216 (6.007–6.425) | 1.342 (1.291–1.396)*** | 1.146 (1.102–1.193)*** | 1.147 (1.102–1.193)*** |
| **CVD mortality** |  |  |  |  |  |  |
| **IPF** |  |  |  |  |  |  |
| No | 3474564.86 | 3504/257612 | 1.008 (0.975–1.042) | 1.000 (ref) | 1.000 (ref) | 1.000 (ref) |
| Yes | 15160.84 | 85/1291 | 5.607 (4.415–6.798) | 6.007 (4.844–7.449)*** | 2.483 (2.000–3.083)*** | 2.438 (1.961–3.030)*** |
| **Smoking status** |  |  |  |  |  |  |
| Never | 2066624.91 | 1588/152291 | 0.768 (0.731–0.806) | 1.000 (ref) | 1.000 (ref) | 1.000 (ref) |
| Former | 1326249.48 | 1868/99402 | 1.408 (1.345–1.472) | 1.848 (1.728–1.976)*** | 1.251 (1.168–1.341)*** | 1.252 (1.168–1.341)*** |
| Current | 96851.32 | 133/7210 | 1.373 (1.140–1.607) | 1.793 (1.502–2.140)*** | 1.654 (1.384–1.977)*** | 1.655 (1.384–1.978)*** |
| **Adult SHS exposure** |  |  |  |  |  |  |
| Never | 2734739.20 | 2612/202832 | 0.955 (0.918–0.992) | 1.000 (ref) | 1.000 (ref) | 1.000 (ref) |
| Ever | 754986.50 | 977/56071 | 1.294 (1.213–1.375) | 1.351 (1.256–1.454)*** | 1.179 (1.093–1.271)*** | 1.178 (1.093–1.270)*** |
| **Maternal smoking exposure** |  |  |  |  |  |  |
| Never | 2434502.90 | 2473/180625 | 1.016 (0.976–1.056) | 1.000 (ref) | 1.000 (ref) | 1.000 (ref) |
| Ever | 1055222.81 | 1116/78278 | 1.058 (0.996–1.120) | 1.040 (0.969–1.116) | 1.084 (1.009–1.164)* | 1.084 (1.009–1.164)* |
| **Passive SHS exposure** |  |  |  |  |  |  |
| Never | 1841425.43 | 1763/136594 | 0.957 (0.913–1.002) | 1.000 (ref) | 1.000 (ref) | 1.000 (ref) |
| Ever | 1648300.27 | 1826/122309 | 1.108 (1.057–1.159) | 1.155 (1.082–1.233)*** | 1.131 (1.058–1.210)*** | 1.131 (1.058–1.209)*** |
| **Pack years** |  |  |  |  |  |  |
| 0 | 2071671.18 | 1596/152680 | 0.770 (0.733–0.808) | 1.000 (ref) | 1.000 (ref) | 1.000 (ref) |
| 1–10 | 262502.31 | 174/19385 | 0.663 (0.564–0.761) | 0.863 (0.738–1.009) | 0.823 (0.704–0.963)* | 0.824 (0.705–0.964)* |
| 11–20 | 252961.55 | 275/18844 | 1.087 (0.959–1.216) | 1.419 (1.249–1.613)*** | 1.058 (0.930–1.204) | 1.059 (0.931–1.205) |
| 21–30 | 161013.58 | 267/12076 | 1.658 (1.459–1.857) | 2.168 (1.904–2.468)*** | 1.357 (1.190–1.548)*** | 1.356 (1.189–1.547)*** |
| >30 | 195908.80 | 622/15314 | 3.175 (2.925–3.424) | 4.222 (3.848–4.631)*** | 1.812 (1.642–1.999)*** | 1.812 (1.643–1.999)*** |
| Unknown | 545668.28 | 655/40604 | 1.200 (1.108–1.292) | 1.567 (1.431–1.716)*** | 1.233 (1.125–1.352)*** | 1.234 (1.126–1.353)*** |
| **Cancer mortality** |  |  |  |  |  |  |
| **IPF** |  |  |  |  |  |  |
| No | 3474564.86 | 10452/257612 | 3.008 (2.950–3.066) | 1.000 (ref) | 1.000 (ref) | 1.000 (ref) |
| Yes | 15160.84 | 152/1291 | 10.026 (8.432–11.620) | 3.473 (2.959–4.076)*** | 2.012 (1.713–2.363)*** | 1.996 (1.699–2.346)*** |
| **Smoking status** |  |  |  |  |  |  |
| Never | 2066624.91 | 5137/152291 | 2.486 (2.418–2.554) | 1.000 (ref) | 1.000 (ref) | 1.000 (ref) |
| Former | 1326249.48 | 5141/99402 | 3.876 (3.770–3.982) | 1.566 (1.507–1.628)*** | 1.277 (1.227–1.330)*** | 1.277 (1.227–1.330)*** |
| Current | 96851.32 | 326/7210 | 3.366 (3.001–3.731) | 1.357 (1.213–1.518)*** | 1.440 (1.286–1.612)*** | 1.440 (1.286–1.612)*** |
| **Adult SHS exposure** |  |  |  |  |  |  |
| Never | 2734739.20 | 8171/202832 | 2.988 (2.923–3.053) | 1.000 (ref) | 1.000 (ref) | 1.000 (ref) |
| Ever | 754986.50 | 2433/56071 | 3.223 (3.095–3.351) | 1.078 (1.030–1.128)** | 1.072 (1.024–1.123)** | 1.072 (1.024–1.123)** |
| **Maternal smoking exposure** |  |  |  |  |  |  |
| Never | 2434502.90 | 7474/180625 | 3.070 (3.000–3.140) | 1.000 (ref) | 1.000 (ref) | 1.000 (ref) |
| Ever | 1055222.81 | 3130/78278 | 2.966 (2.862–3.070) | 0.966 (0.926–1.007) | 1.020 (0.978–1.064) | 1.020 (0.977–1.063) |
| **Passive SHS exposure** |  |  |  |  |  |  |
| Never | 1841425.43 | 5570/136594 | 3.025 (2.945–3.104) | 1.000 (ref) | 1.000 (ref) | 1.000 (ref) |
| Ever | 1648300.27 | 5034/122309 | 3.054 (2.970–3.138) | 1.009 (0.971–1.048) | 1.055 (1.015–1.097)** | 1.055 (1.015–1.097)** |
| **Pack years** |  |  |  |  |  |  |
| 0 | 2071671.18 | 5169/152680 | 2.495 (2.427–2.563) | 1.000 (ref) | 1.000 (ref) | 1.000 (ref) |
| 1–10 | 262502.31 | 727/19385 | 2.769 (2.568–2.971) | 1.111 (1.028–1.201)** | 1.071 (0.991–1.158) | 1.071 (0.991–1.158) |
| 11–20 | 252961.55 | 871/18844 | 3.443 (3.215–3.672) | 1.384 (1.288–1.487)*** | 1.197 (1.114–1.288)*** | 1.198 (1.114–1.288)*** |
| 21–30 | 161013.58 | 662/12076 | 4.111 (3.798–4.425) | 1.654 (1.526–1.794)*** | 1.313 (1.209–1.425)*** | 1.313 (1.209–1.425)*** |
| >30 | 195908.80 | 1510/15314 | 7.708 (7.319–8.096) | 3.132 (2.957–3.317)*** | 2.037 (1.916–2.165)*** | 2.037 (1.916–2.165)*** |
| Unknown | 545668.28 | 1665/40604 | 3.051 (2.905–3.198) | 1.226 (1.160–1.296)*** | 1.092 (1.033–1.155)** | 1.092 (1.033–1.155)** |
| **Respiratory mortality** |  |  |  |  |  |  |
| **IPF** |  |  |  |  |  |  |
| No | 3474564.86 | 702/257612 | 0.202 (0.187–0.217) | 1.000 (ref) | 1.000 (ref) | 1.000 (ref) |
| Yes | 15160.84 | 339/1291 | 22.360 (19.980–24.741) | 121.313 (106.525–138.154)*** | 52.050 (45.372–59.712)*** | 51.288 (44.500–59.112)*** |
| **Smoking status** |  |  |  |  |  |  |
| Never | 2066624.91 | 334/152291 | 0.162 (0.144–0.179) | 1.000 (ref) | 1.000 (ref) | 1.000 (ref) |
| Former | 1326249.48 | 665/99402 | 0.501 (0.463–0.540) | 3.130 (2.744–3.569)*** | 2.087 (1.823–2.390)*** | 2.085 (1.821–2.388)*** |
| Current | 96851.32 | 42/7210 | 0.434 (0.303–0.565) | 2.694 (1.955–3.713)*** | 2.549 (1.844–3.523)*** | 2.534 (1.833–3.504)*** |
| **Adult SHS exposure** |  |  |  |  |  |  |
| Never | 2734739.20 | 723/202832 | 0.264 (0.245–0.284) | 1.000 (ref) | 1.000 (ref) | 1.000 (ref) |
| Ever | 754986.50 | 318/56071 | 0.421 (0.375–0.467) | 1.591 (1.394–1.815)*** | 1.388 (1.212–1.588)*** | 1.386 (1.211–1.587)*** |
| **Maternal smoking exposure** |  |  |  |  |  |  |
| Never | 2434502.90 | 685/180625 | 0.281 (0.260–0.302) | 1.000 (ref) | 1.000 (ref) | 1.000 (ref) |
| Ever | 1055222.81 | 356/78278 | 0.337 (0.302–0.372) | 1.198 (1.054–1.362)** | 1.278 (1.122–1.455)*** | 1.272 (1.117–1.448)*** |
| **Passive SHS exposure** |  |  |  |  |  |  |
| Never | 1841425.43 | 453/136594 | 0.246 (0.223–0.269) | 1.000 (ref) | 1.000 (ref) | 1.000 (ref) |
| Ever | 1648300.27 | 588/122309 | 0.357 (0.328–0.386) | 1.448 (1.281–1.637)*** | 1.449 (1.279–1.643)*** | 1.444 (1.274–1.636)*** |
| **Pack years** |  |  |  |  |  |  |
| 0 | 2071671.18 | 343/152680 | 0.166 (0.148–0.183) | 1.000 (ref) | 1.000 (ref) | 1.000 (ref) |
| 1–10 | 262502.31 | 48/19385 | 0.183 (0.131–0.235) | 1.107 (0.819–1.498) | 1.065 (0.786–1.441) | 1.063 (0.785–1.439) |
| 11–20 | 252961.55 | 85/18844 | 0.336 (0.265–0.407) | 2.042 (1.610–2.589)*** | 1.552 (1.221–1.973)*** | 1.558 (1.225–1.980)*** |
| 21–30 | 161013.58 | 90/12076 | 0.559 (0.443–0.674) | 3.402 (2.697–4.291)*** | 2.176 (1.719–2.755)*** | 2.167 (1.712–2.744)*** |
| >30 | 195908.80 | 319/15314 | 1.628 (1.450–1.807) | 10.106 (8.677–11.771)*** | 4.355 (3.694–5.133)*** | 4.341 (3.683–5.117)*** |
| Unknown | 545668.28 | 156/40604 | 0.286 (0.241–0.331) | 1.737 (1.437–2.099)*** | 1.401 (1.157–1.696)*** | 1.400 (1.157–1.695)*** |

Abbreviations: PYs, person-years; Cases/N, number of deaths/total number of participants; HR, hazard ratio; CI, confidence interval; CVD, cardiovascular disease; BMI, body mass index; PA, physical activity; SHS, secondhand smoke; SNP, single nucleotide polymorphism. Incidence rates are presented per 1,000 person-years, and “ref” indicates the reference category.

Hazard ratios (HRs) and 95% confidence intervals (CIs) were estimated using Cox proportional hazards regression. Model 1 was unadjusted. Model 2 was adjusted for sex, age at recruitment, body mass index (BMI), household income, educational attainment, Townsend deprivation index quartiles, physical activity (PA), alcohol consumption, and diet. Model 3 was additionally adjusted for candidate SNPs. “Ever” indicates any exposure versus never exposure (reference). Passive SHS exposure was defined as a composite measure of secondhand smoke exposure. Statistical significance is indicated as *P < 0.05, **P < 0.01, and ***P < 0.001.

**Supplementary Table S12. Joint associations of smoking-related exposures and IPF with all-cause and cause-specific mortality during follow-up among White participants**

| **Joint variables** | **Cases/N** | **HR (95% CI)** | | |
| --- | --- | --- | --- | --- |
|  |  | **Model 1** | **Model 2** | **Model 3** |
| **All-cause mortality** |  |  |  |  |
| Smoking status |  |  |  |  |
| Non-current * No IPF | 19015/250453 | 1.000 (ref) | 1.000 (ref) | 1.000 (ref) |
| Non-current * IPF | 633/1240 | 8.283 (7.652–8.966)*** | 4.224 (3.899–4.575)*** | 4.200 (3.875–4.551)*** |
| Current * No IPF | 629/7159 | 1.161 (1.073–1.257)*** | 1.312 (1.211–1.421)*** | 1.312 (1.211–1.421)*** |
| Current * IPF | 29/51 | 8.822 (6.129–12.698)*** | 4.791 (3.328–6.898)*** | 4.774 (3.316–6.875)*** |
| Adult SHS exposure |  |  |  |  |
| Never * No IPF | 14815/201908 | 1.000 (ref) | 1.000 (ref) | 1.000 (ref) |
| Never * IPF | 461/924 | 8.259 (7.528–9.062)*** | 4.251 (3.873–4.667)*** | 4.227 (3.848–4.642)*** |
| Ever * No IPF | 4829/55704 | 1.180 (1.143–1.219)*** | 1.125 (1.088–1.163)*** | 1.125 (1.088–1.163)*** |
| Ever * IPF | 201/367 | 9.462 (8.232–10.875)*** | 4.570 (3.973–5.257)*** | 4.548 (3.953–5.232)*** |
| Maternal smoking exposure |  |  |  |  |
| Never * No IPF | 13757/179767 | 1.000 (ref) | 1.000 (ref) | 1.000 (ref) |
| Never * IPF | 443/858 | 8.260 (7.514–9.080)*** | 4.097 (3.725–4.507)*** | 4.077 (3.705–4.486)*** |
| Ever * No IPF | 5887/77845 | 0.987 (0.958–1.018) | 1.042 (1.010–1.074)** | 1.041 (1.010–1.074)** |
| Ever * IPF | 219/433 | 8.189 (7.165–9.358)*** | 4.645 (4.063–5.311)*** | 4.615 (4.035–5.278)*** |
| Passive SHS exposure |  |  |  |  |
| Never * No IPF | 10065/136005 | 1.000 (ref) | 1.000 (ref) | 1.000 (ref) |
| Never * IPF | 298/589 | 8.262 (7.363–9.271)*** | 4.168 (3.713–4.680)*** | 4.147 (3.693–4.657)*** |
| Ever * No IPF | 9579/121607 | 1.063 (1.034–1.093)*** | 1.093 (1.062–1.125)*** | 1.093 (1.062–1.124)*** |
| Ever * IPF | 364/702 | 8.733 (7.866–9.696)*** | 4.612 (4.151–5.124)*** | 4.585 (4.125–5.097)*** |
| **CVD mortality** |  |  |  |  |
| Smoking status |  |  |  |  |
| Non-current * No IPF | 3373/250453 | 1.000 (ref) | 1.000 (ref) | 1.000 (ref) |
| Non-current * IPF | 83/1240 | 6.180 (4.970–7.684)*** | 2.556 (2.053–3.181)*** | 2.508 (2.013–3.126)*** |
| Current * No IPF | 131/7159 | 1.363 (1.145–1.623)*** | 1.486 (1.247–1.771)*** | 1.486 (1.247–1.770)*** |
| Current * IPF | 2/51 | 3.453 (0.863–13.813) | 1.503 (0.376–6.013) | 1.493 (0.373–5.976) |
| Adult SHS exposure |  |  |  |  |
| Never * No IPF | 2549/201908 | 1.000 (ref) | 1.000 (ref) | 1.000 (ref) |
| Never * IPF | 63/924 | 6.621 (5.156–8.502)*** | 2.807 (2.184–3.608)*** | 2.756 (2.142–3.545)*** |
| Ever * No IPF | 955/55704 | 1.355 (1.258–1.460)*** | 1.187 (1.100–1.281)*** | 1.187 (1.100–1.280)*** |
| Ever * IPF | 22/367 | 6.070 (3.989–9.235)*** | 2.157 (1.416–3.287)*** | 2.119 (1.390–3.230)*** |
| Maternal smoking exposure |  |  |  |  |
| Never * No IPF | 2412/179767 | 1.000 (ref) | 1.000 (ref) | 1.000 (ref) |
| Never * IPF | 61/858 | 6.546 (5.077–8.440)*** | 2.612 (2.023–3.371)*** | 2.570 (1.989–3.320)*** |
| Ever * No IPF | 1092/77845 | 1.044 (0.972–1.122) | 1.088 (1.012–1.169)* | 1.088 (1.012–1.169)* |
| Ever * IPF | 24/433 | 5.167 (3.457–7.725)*** | 2.397 (1.602–3.586)*** | 2.343 (1.565–3.508)*** |
| Passive SHS exposure |  |  |  |  |
| Never * No IPF | 1719/136005 | 1.000 (ref) | 1.000 (ref) | 1.000 (ref) |
| Never * IPF | 44/589 | 7.206 (5.342–9.720)*** | 2.989 (2.214–4.036)*** | 2.941 (2.177–3.973)*** |
| Ever * No IPF | 1785/121607 | 1.159 (1.085–1.239)*** | 1.138 (1.064–1.218)*** | 1.138 (1.064–1.218)*** |
| Ever * IPF | 41/702 | 5.813 (4.264–7.923)*** | 2.365 (1.733–3.228)*** | 2.318 (1.696–3.167)*** |
| **Cancer mortality** |  |  |  |  |
| Smoking status |  |  |  |  |
| Non-current * No IPF | 10135/250453 | 1.000 (ref) | 1.000 (ref) | 1.000 (ref) |
| Non-current * IPF | 143/1240 | 3.416 (2.896–4.030)*** | 1.981 (1.678–2.338)*** | 1.965 (1.664–2.321)*** |
| Current * No IPF | 317/7159 | 1.098 (0.982–1.228) | 1.259 (1.125–1.409)*** | 1.259 (1.125–1.409)*** |
| Current * IPF | 9/51 | 5.023 (2.613–9.657)*** | 3.117 (1.621–5.995)*** | 3.099 (1.611–5.959)*** |
| Adult SHS exposure |  |  |  |  |
| Never * No IPF | 8064/201908 | 1.000 (ref) | 1.000 (ref) | 1.000 (ref) |
| Never * IPF | 107/924 | 3.431 (2.835–4.152)*** | 1.995 (1.648–2.416)*** | 1.979 (1.634–2.398)*** |
| Ever * No IPF | 2388/55704 | 1.074 (1.026–1.124)** | 1.070 (1.022–1.121)** | 1.070 (1.022–1.121)** |
| Ever * IPF | 45/367 | 3.784 (2.823–5.072)*** | 2.178 (1.623–2.921)*** | 2.162 (1.611–2.900)*** |
| Maternal smoking exposure |  |  |  |  |
| Never * No IPF | 7372/179767 | 1.000 (ref) | 1.000 (ref) | 1.000 (ref) |
| Never * IPF | 102/858 | 3.457 (2.843–4.203)*** | 1.963 (1.614–2.388)*** | 1.948 (1.601–2.371)*** |
| Ever * No IPF | 3080/77845 | 0.964 (0.924–1.006) | 1.018 (0.976–1.062) | 1.018 (0.976–1.062) |
| Ever * IPF | 50/433 | 3.392 (2.569–4.480)*** | 2.156 (1.632–2.849)*** | 2.137 (1.617–2.824)*** |
| Passive SHS exposure |  |  |  |  |
| Never * No IPF | 5503/136005 | 1.000 (ref) | 1.000 (ref) | 1.000 (ref) |
| Never * IPF | 67/589 | 3.312 (2.603–4.215)*** | 1.898 (1.491–2.416)*** | 1.885 (1.480–2.400)*** |
| Ever * No IPF | 4949/121607 | 1.005 (0.967–1.045) | 1.052 (1.012–1.094)* | 1.052 (1.012–1.094)* |
| Ever * IPF | 85/702 | 3.628 (2.928–4.494)*** | 2.213 (1.785–2.743)*** | 2.194 (1.769–2.721)*** |
| **Respiratory mortality** |  |  |  |  |
| Smoking status |  |  |  |  |
| Non-current * No IPF | 677/250453 | 1.000 (ref) | 1.000 (ref) | 1.000 (ref) |
| Non-current * IPF | 322/1240 | 121.186 (106.089–138.431)*** | 51.993 (45.191–59.819)*** | 51.258 (44.343–59.251)*** |
| Current * No IPF | 25/7159 | 1.297 (0.870–1.933) | 1.445 (0.968–2.156) | 1.444 (0.967–2.155) |
| Current * IPF | 17/51 | 148.307 (91.645–240.003)*** | 68.626 (42.230–111.522)*** | 66.348 (40.782–107.940)*** |
| Adult SHS exposure |  |  |  |  |
| Never * No IPF | 490/201908 | 1.000 (ref) | 1.000 (ref) | 1.000 (ref) |
| Never * IPF | 233/924 | 129.008 (110.345–150.828)*** | 56.057 (47.660–65.933)*** | 55.141 (46.688–65.124)*** |
| Ever * No IPF | 212/55704 | 1.566 (1.333–1.840)*** | 1.425 (1.210–1.678)*** | 1.425 (1.210–1.678)*** |
| Ever * IPF | 106/367 | 155.079 (125.681–191.353)*** | 61.320 (49.226–76.385)*** | 60.691 (48.570–75.837)*** |
| Maternal smoking exposure |  |  |  |  |
| Never * No IPF | 467/179767 | 1.000 (ref) | 1.000 (ref) | 1.000 (ref) |
| Never * IPF | 218/858 | 122.526 (104.301–143.937)*** | 50.519 (42.732–59.726)*** | 49.683 (41.874–58.948)*** |
| Ever * No IPF | 235/77845 | 1.161 (0.992–1.358) | 1.239 (1.058–1.451)** | 1.237 (1.056–1.449)** |
| Ever * IPF | 121/433 | 136.602 (111.818–166.879)*** | 67.762 (55.191–83.196)*** | 67.358 (54.617–83.072)*** |
| Passive SHS exposure |  |  |  |  |
| Never * No IPF | 304/136005 | 1.000 (ref) | 1.000 (ref) | 1.000 (ref) |
| Never * IPF | 149/589 | 139.736 (114.839–170.030)*** | 58.511 (47.834–71.571)*** | 57.534 (46.900–70.579)*** |
| Ever * No IPF | 398/121607 | 1.462 (1.259–1.697)*** | 1.493 (1.283–1.737)*** | 1.492 (1.282–1.736)*** |
| Ever * IPF | 190/702 | 154.770 (129.077–185.577)*** | 69.803 (57.751–84.370)*** | 69.060 (56.891–83.833)*** |

Abbreviations: HR, hazard ratio; CI, confidence interval; CVD, cardiovascular disease; BMI, body mass index; PA, physical activity; SHS, secondhand smoke; SNP, single nucleotide polymorphism. Cases/N indicates the number of deaths/total number of participants, and “ref” indicates the reference category.

Hazard ratios (HRs) and 95% confidence intervals (CIs) were estimated using Cox proportional hazards regression. Model 1 was unadjusted. Model 2 was adjusted for sex, age at recruitment, body mass index (BMI), household income, educational attainment, Townsend deprivation index quartiles, physical activity (PA), alcohol consumption, and diet. Model 3 was additionally adjusted for candidate SNPs. “Ever” indicates any exposure versus never exposure (reference).For smoking status, the exposed category was current smoking and the reference category was non-current smoking, which included never and former smokers. For passive smoking exposures, exposed participants were compared with unexposed participants. Statistical significance is indicated as *P < 0.05, **P < 0.01, and ***P < 0.001.

**Supplementary Table S13. Additive interactions between smoking-related exposures and IPF in relation to all-cause and cause-specific mortality during follow-up among White participants**

| **Additive interaction** | **Index for additive interaction** | **All-cause mortality** | **CVD mortality** | **Cancer mortality** | **Respiratory mortality** |
| --- | --- | --- | --- | --- | --- |
| Smoking status | RERI | 0.263 (-1.509–2.034) | -1.501 (-3.657–0.655) | 0.874 (-1.181–2.929) | 14.646 (-17.595–46.887) |
|  | AP | 0.055 (-0.297–0.407) | -1.005 (-3.813–1.803) | 0.282 (-0.200–0.765) | 0.221 (-0.161–0.603) |
|  | SI | 1.075 (0.671–1.721) | 0.247 (0.004–16.627) | 1.714 (0.626–4.691) | 1.289 (0.783–2.121) |
| Adult SHS exposure | RERI | 0.196 (-0.543–0.936) | -0.824 (-1.946–0.299) | 0.112 (-0.624–0.848) | 5.125 (-8.667–18.918) |
|  | AP | 0.043 (-0.114–0.201) | -0.389 (-1.054–0.276) | 0.052 (-0.276–0.380) | 0.084 (-0.129–0.298) |
|  | SI | 1.059 (0.857–1.308) | 0.576 (0.242–1.371) | 1.107 (0.576–2.125) | 1.094 (0.863–1.386) |
| Maternal smoking exposure | RERI | 0.496 (-0.224–1.217) | -0.314 (-1.456–0.827) | 0.170 (-0.534–0.874) | **17.438 (3.482–31.393)** |
|  | AP | 0.108 (-0.037–0.252) | -0.134 (-0.666–0.398) | 0.080 (-0.231–0.391) | 0.259 (0.093–0.425) |
|  | SI | 1.159 (0.941–1.428) | 0.810 (0.364–1.805) | 1.176 (0.612–2.261) | 1.356 (1.080–1.703) |
| Passive SHS exposure | RERI | 0.345 (-0.322–1.013) | -0.761 (-1.888–0.366) | 0.257 (-0.393–0.907) | 11.034 (-2.750–24.819) |
|  | AP | 0.075 (-0.065–0.215) | -0.328 (-0.883–0.226) | 0.117 (-0.162–0.396) | 0.160 (-0.022–0.342) |
|  | SI | 1.107 (0.909–1.347) | 0.634 (0.320–1.255) | 1.275 (0.684–2.375) | 1.193 (0.958–1.487) |

Abbreviations: RERI, relative excess risk due to interaction; AP, attributable proportion; SI, synergy index; CI, confidence interval; CVD, cardiovascular disease; BMI, body mass index; PA, physical activity; SHS, secondhand smoke; SNP, single nucleotide polymorphism.

Additive interactions between smoking-related exposures and IPF in relation to all-cause and cause-specific mortality during follow-up among White participants were evaluated using RERI, AP, and SI with 95% CIs from Model 3. Model 3 was adjusted for sex, age at recruitment, body mass index (BMI), household income, educational attainment, Townsend deprivation index quartiles, physical activity (PA), alcohol consumption, diet, and candidate SNPs. For smoking status, the exposed category was current smoking and the reference category was non-current smoking, which included never and former smokers. For passive smoking exposures, exposed participants were compared with unexposed participants. Boldface indicates RERI estimates for which the 95% confidence interval does not include zero.

**Supplementary Table S14. Multiplicative interactions between smoking-related exposures and IPF in relation to all-cause and cause-specific mortality during follow-up among White participants**

| **interaction** | **HR (95% CI)** | | | |
| --- | --- | --- | --- | --- |
|  | **All-cause mortality** | **CVD mortality** | **Cancer mortality** | **Respiratory mortality** |
| Smoking status | 0.867 (0.592–1.268) | 0.401 (0.097–1.647) | 1.252 (0.633–2.479) | 0.896 (0.476–1.687) |
| Adult SHS exposure | 0.957 (0.808–1.133) | 0.648 (0.396–1.059) | 1.020 (0.718–1.450) | 0.773 (0.583–1.024) |
| Maternal smoking exposure | 1.087 (0.922–1.282) | 0.838 (0.520–1.352) | 1.077 (0.766–1.515) | 1.096 (0.834–1.440) |
| Passive SHS exposure | 1.012 (0.866–1.182) | 0.693 (0.450–1.066) | 1.106 (0.801–1.528) | 0.805 (0.618–1.047) |

Abbreviations: HR, hazard ratio; CI, confidence interval; CVD, cardiovascular disease; BMI, body mass index; PA, physical activity; SHS, secondhand smoke; SNP, single nucleotide polymorphism.

Multiplicative interactions between smoking-related exposures and IPF in relation to all-cause and cause-specific mortality during follow-up among White participants were evaluated using the HR and 95% CI for the interaction term from Model 3. Model 3 was adjusted for sex, age at recruitment, body mass index (BMI), household income, educational attainment, Townsend deprivation index quartiles, physical activity (PA), alcohol consumption, diet, and candidate SNPs. For smoking status, the exposed category was current smoking and the reference category was non-current smoking, which included never and former smokers. For passive smoking exposures, exposed participants were compared with unexposed participants. Statistical significance is indicated as *P < 0.05, **P < 0.01, and ***P < 0.001.

**Supplementary Table S15. Independent associations of smoking-related exposures and IPF with all-cause and cause-specific mortality during follow-up among participants with complete covariate data**

| **Exposure** | **Person-years**  **(PYs)** | **Cases/N** | **Incidence Rate**  **(95% CI)** | **HR (95% CI)** | | |
| --- | --- | --- | --- | --- | --- | --- |
|  |  |  |  | **Model 1** | **Model 2** | **Model 3** |
| **All-cause mortality** |  |  |  |  |  |  |
| **IPF** |  |  |  |  |  |  |
| No | 2701242.35 | 12800/199708 | 4.739 (4.656–4.821) | 1.000 (ref) | 1.000 (ref) | 1.000 (ref) |
| Yes | 8651.76 | 379/745 | 43.806 (39.396–48.216) | 9.914 (8.951–10.981)*** | 5.077 (4.580–5.629)*** | 5.077 (4.577–5.631)*** |
| **Smoking status** |  |  |  |  |  |  |
| Never | 1649737.31 | 6609/121448 | 4.006 (3.910–4.103) | 1.000 (ref) | 1.000 (ref) | 1.000 (ref) |
| Former | 980349.17 | 6160/73095 | 6.283 (6.127–6.440) | 1.577 (1.523–1.632)*** | 1.206 (1.164–1.251)*** | 1.206 (1.164–1.250)*** |
| Current | 79807.64 | 410/5910 | 5.137 (4.640–5.635) | 1.285 (1.163–1.420)*** | 1.319 (1.193–1.459)*** | 1.319 (1.193–1.459)*** |
| **Adult SHS exposure** |  |  |  |  |  |  |
| Never | 2128071.16 | 10061/157404 | 4.728 (4.635–4.820) | 1.000 (ref) | 1.000 (ref) | 1.000 (ref) |
| Ever | 581822.95 | 3118/43049 | 5.359 (5.171–5.547) | 1.132 (1.087–1.178)*** | 1.108 (1.064–1.155)*** | 1.108 (1.064–1.154)*** |
| **Maternal smoking exposure** |  |  |  |  |  |  |
| Never | 1927953.36 | 9406/142675 | 4.879 (4.780–4.977) | 1.000 (ref) | 1.000 (ref) | 1.000 (ref) |
| Ever | 781940.76 | 3773/57778 | 4.825 (4.671–4.979) | 0.987 (0.951–1.025) | 1.035 (0.996–1.075) | 1.034 (0.996–1.075) |
| **Passive SHS exposure** |  |  |  |  |  |  |
| Never | 1454470.84 | 6930/107622 | 4.765 (4.652–4.877) | 1.000 (ref) | 1.000 (ref) | 1.000 (ref) |
| Ever | 1255423.27 | 6249/92831 | 4.978 (4.854–5.101) | 1.043 (1.008–1.080)* | 1.081 (1.044–1.119)*** | 1.081 (1.044–1.119)*** |
| **Pack years** |  |  |  |  |  |  |
| 0 | 1652818.74 | 6632/121681 | 4.013 (3.916–4.109) | 1.000 (ref) | 1.000 (ref) | 1.000 (ref) |
| 1–10 | 211106.86 | 866/15556 | 4.102 (3.829–4.375) | 1.024 (0.954–1.099) | 0.957 (0.891–1.027) | 0.956 (0.891–1.027) |
| 11–20 | 189789.30 | 1038/14094 | 5.469 (5.136–5.802) | 1.368 (1.282–1.461)*** | 1.108 (1.037–1.184)** | 1.108 (1.037–1.184)** |
| 21–30 | 113175.98 | 839/8477 | 7.413 (6.912–7.915) | 1.858 (1.730–1.997)*** | 1.326 (1.232–1.426)*** | 1.325 (1.232–1.426)*** |
| >30 | 120055.24 | 1620/9289 | 13.494 (12.837–14.151) | 3.422 (3.241–3.613)*** | 1.864 (1.760–1.974)*** | 1.863 (1.760–1.973)*** |
| Unknown | 422947.99 | 2184/31356 | 5.164 (4.947–5.380) | 1.291 (1.230–1.355)*** | 1.100 (1.047–1.155)*** | 1.100 (1.047–1.155)*** |
| **CVD mortality** |  |  |  |  |  |  |
| **IPF** |  |  |  |  |  |  |
| No | 2701242.35 | 2190/199708 | 0.811 (0.777–0.845) | 1.000 (ref) | 1.000 (ref) | 1.000 (ref) |
| Yes | 8651.76 | 46/745 | 5.317 (3.780–6.853) | 7.120 (5.316–9.535)*** | 2.867 (2.137–3.845)*** | 2.807 (2.090–3.771)*** |
| **Smoking status** |  |  |  |  |  |  |
| Never | 1649737.31 | 1066/121448 | 0.646 (0.607–0.685) | 1.000 (ref) | 1.000 (ref) | 1.000 (ref) |
| Former | 980349.17 | 1077/73095 | 1.099 (1.033–1.164) | 1.711 (1.572–1.862)*** | 1.148 (1.051–1.253)** | 1.148 (1.052–1.253)** |
| Current | 79807.64 | 93/5910 | 1.165 (0.928–1.402) | 1.808 (1.463–2.235)*** | 1.649 (1.331–2.042)*** | 1.647 (1.329–2.039)*** |
| **Adult SHS exposure** |  |  |  |  |  |  |
| Never | 2128071.16 | 1652/157404 | 0.776 (0.739–0.814) | 1.000 (ref) | 1.000 (ref) | 1.000 (ref) |
| Ever | 581822.95 | 584/43049 | 1.004 (0.922–1.085) | 1.290 (1.174–1.418)*** | 1.150 (1.044–1.266)** | 1.149 (1.043–1.265)** |
| **Maternal smoking exposure** |  |  |  |  |  |  |
| Never | 1927953.36 | 1565/142675 | 0.812 (0.772–0.852) | 1.000 (ref) | 1.000 (ref) | 1.000 (ref) |
| Ever | 781940.76 | 671/57778 | 0.858 (0.793–0.923) | 1.055 (0.964–1.155) | 1.104 (1.007–1.210)* | 1.104 (1.008–1.211)* |
| **Passive SHS exposure** |  |  |  |  |  |  |
| Never | 1454470.84 | 1138/107622 | 0.782 (0.737–0.828) | 1.000 (ref) | 1.000 (ref) | 1.000 (ref) |
| Ever | 1255423.27 | 1098/92831 | 0.875 (0.823–0.926) | 1.116 (1.027–1.212)** | 1.104 (1.014–1.201)* | 1.103 (1.014–1.201)* |
| **Pack years** |  |  |  |  |  |  |
| 0 | 1652818.74 | 1073/121681 | 0.649 (0.610–0.688) | 1.000 (ref) | 1.000 (ref) | 1.000 (ref) |
| 1–10 | 211106.86 | 120/15556 | 0.568 (0.467–0.670) | 0.877 (0.726–1.059) | 0.808 (0.668–0.976)* | 0.807 (0.668–0.975)* |
| 11–20 | 189789.30 | 157/14094 | 0.827 (0.698–0.957) | 1.280 (1.083–1.514)** | 0.924 (0.781–1.095) | 0.925 (0.781–1.096) |
| 21–30 | 113175.98 | 177/8477 | 1.564 (1.334–1.794) | 2.425 (2.069–2.843)*** | 1.433 (1.219–1.685)*** | 1.433 (1.219–1.684)*** |
| >30 | 120055.24 | 297/9289 | 2.474 (2.193–2.755) | 3.888 (3.419–4.421)*** | 1.567 (1.369–1.794)*** | 1.568 (1.370–1.795)*** |
| Unknown | 422947.99 | 412/31356 | 0.974 (0.880–1.068) | 1.507 (1.345–1.688)*** | 1.171 (1.043–1.314)** | 1.171 (1.044–1.314)** |
| **Cancer mortality** |  |  |  |  |  |  |
| **IPF** |  |  |  |  |  |  |
| No | 2701242.35 | 7101/199708 | 2.629 (2.568–2.690) | 1.000 (ref) | 1.000 (ref) | 1.000 (ref) |
| Yes | 8651.76 | 95/745 | 10.980 (8.772–13.188) | 4.367 (3.567–5.347)*** | 2.535 (2.068–3.106)*** | 2.529 (2.062–3.101)*** |
| **Smoking status** |  |  |  |  |  |  |
| Never | 1649737.31 | 3709/121448 | 2.248 (2.176–2.321) | 1.000 (ref) | 1.000 (ref) | 1.000 (ref) |
| Former | 980349.17 | 3289/73095 | 3.355 (3.240–3.470) | 1.497 (1.428–1.569)*** | 1.219 (1.161–1.280)*** | 1.219 (1.161–1.280)*** |
| Current | 79807.64 | 198/5910 | 2.481 (2.135–2.827) | 1.105 (0.958–1.275) | 1.196 (1.035–1.381)* | 1.196 (1.036–1.381)* |
| **Adult SHS exposure** |  |  |  |  |  |  |
| Never | 2128071.16 | 5629/157404 | 2.645 (2.576–2.714) | 1.000 (ref) | 1.000 (ref) | 1.000 (ref) |
| Ever | 581822.95 | 1567/43049 | 2.693 (2.560–2.827) | 1.018 (0.962–1.076) | 1.041 (0.983–1.102) | 1.041 (0.983–1.102) |
| **Maternal smoking exposure** |  |  |  |  |  |  |
| Never | 1927953.36 | 5197/142675 | 2.696 (2.622–2.769) | 1.000 (ref) | 1.000 (ref) | 1.000 (ref) |
| Ever | 781940.76 | 1999/57778 | 2.556 (2.444–2.669) | 0.948 (0.900–0.998)* | 0.988 (0.938–1.041) | 0.988 (0.937–1.040) |
| **Passive SHS exposure** |  |  |  |  |  |  |
| Never | 1454470.84 | 3892/107622 | 2.676 (2.592–2.760) | 1.000 (ref) | 1.000 (ref) | 1.000 (ref) |
| Ever | 1255423.27 | 3304/92831 | 2.632 (2.542–2.722) | 0.983 (0.938–1.030) | 1.033 (0.985–1.083) | 1.033 (0.985–1.082) |
| **Pack years** |  |  |  |  |  |  |
| 0 | 1652818.74 | 3720/121681 | 2.251 (2.178–2.323) | 1.000 (ref) | 1.000 (ref) | 1.000 (ref) |
| 1–10 | 211106.86 | 520/15556 | 2.463 (2.251–2.675) | 1.095 (0.999–1.200) | 1.032 (0.941–1.132) | 1.032 (0.941–1.132) |
| 11–20 | 189789.30 | 597/14094 | 3.146 (2.893–3.398) | 1.401 (1.285–1.527)*** | 1.199 (1.099–1.309)*** | 1.199 (1.098–1.308)*** |
| 21–30 | 113175.98 | 423/8477 | 3.738 (3.381–4.094) | 1.667 (1.508–1.844)*** | 1.304 (1.177–1.444)*** | 1.304 (1.177–1.444)*** |
| >30 | 120055.24 | 818/9289 | 6.814 (6.347–7.280) | 3.063 (2.839–3.303)*** | 1.949 (1.800–2.111)*** | 1.949 (1.800–2.111)*** |
| Unknown | 1652818.74 | 3720/121681 | 2.251 (2.178–2.323) | 1.000 (ref) | 1.000 (ref) | 1.000 (ref) |
| **Respiratory mortality** |  |  |  |  |  |  |
| **IPF** |  |  |  |  |  |  |
| No | 2701242.35 | 378/199708 | 0.140 (0.126–0.154) | 1.000 (ref) | 1.000 (ref) | 1.000 (ref) |
| Yes | 8651.76 | 189/745 | 21.845 (18.731–24.960) | 170.215 (142.872–202.791)*** | 75.376 (62.552–90.830)*** | 74.953 (61.833–90.857)*** |
| **Smoking status** |  |  |  |  |  |  |
| Never | 1649737.31 | 198/121448 | 0.120 (0.103–0.137) | 1.000 (ref) | 1.000 (ref) | 1.000 (ref) |
| Former | 980349.17 | 351/73095 | 0.358 (0.321–0.395) | 3.001 (2.522–3.573)*** | 2.020 (1.687–2.419)*** | 2.018 (1.685–2.416)*** |
| Current | 79807.64 | 18/5910 | 0.226 (0.121–0.330) | 1.884 (1.163–3.052)* | 1.849 (1.137–3.008)* | 1.845 (1.134–3.001)* |
| **Adult SHS exposure** |  |  |  |  |  |  |
| Never | 2128071.16 | 389/157404 | 0.183 (0.165–0.201) | 1.000 (ref) | 1.000 (ref) | 1.000 (ref) |
| Ever | 581822.95 | 178/43049 | 0.306 (0.261–0.351) | 1.670 (1.399–1.995)*** | 1.564 (1.305–1.875)*** | 1.559 (1.300–1.869)*** |
| **Maternal smoking exposure** |  |  |  |  |  |  |
| Never | 1927953.36 | 376/142675 | 0.195 (0.175–0.215) | 1.000 (ref) | 1.000 (ref) | 1.000 (ref) |
| Ever | 781940.76 | 191/57778 | 0.244 (0.210–0.279) | 1.251 (1.051–1.488)* | 1.354 (1.134–1.615)*** | 1.349 (1.131–1.610)*** |
| **Passive SHS exposure** |  |  |  |  |  |  |
| Never | 1454470.84 | 251/107622 | 0.173 (0.151–0.194) | 1.000 (ref) | 1.000 (ref) | 1.000 (ref) |
| Ever | 1255423.27 | 316/92831 | 0.252 (0.224–0.279) | 1.456 (1.234–1.719)*** | 1.521 (1.285–1.801)*** | 1.516 (1.281–1.794)*** |
| **Pack years** |  |  |  |  |  |  |
| 0 | 1652818.74 | 199/121681 | 0.120 (0.104–0.137) | 1.000 (ref) | 1.000 (ref) | 1.000 (ref) |
| 1–10 | 211106.86 | 31/15556 | 0.147 (0.095–0.199) | 1.222 (0.837–1.784) | 1.118 (0.765–1.634) | 1.115 (0.763–1.629) |
| 11–20 | 189789.30 | 46/14094 | 0.242 (0.172–0.312) | 2.022 (1.467–2.787)*** | 1.523 (1.101–2.106)* | 1.522 (1.101–2.105)* |
| 21–30 | 113175.98 | 50/8477 | 0.442 (0.319–0.564) | 3.694 (2.709–5.037)*** | 2.347 (1.711–3.219)*** | 2.343 (1.708–3.214)*** |
| >30 | 120055.24 | 155/9289 | 1.291 (1.088–1.494) | 10.947 (8.874–13.505)*** | 4.783 (3.812–6.001)*** | 4.748 (3.784–5.957)*** |
| Unknown | 422947.99 | 86/31356 | 0.203 (0.160–0.246) | 1.696 (1.317–2.184)*** | 1.362 (1.055–1.759)* | 1.364 (1.056–1.761)* |

Abbreviations: PYs, person-years; Cases/N, number of deaths/total number of participants; HR, hazard ratio; CI, confidence interval; CVD, cardiovascular disease; BMI, body mass index; PA, physical activity; SHS, secondhand smoke; SNP, single nucleotide polymorphism. Incidence rates are presented per 1,000 person-years, and “ref” indicates the reference category.

Hazard ratios (HRs) and 95% confidence intervals (CIs) were estimated using Cox proportional hazards regression. Model 1 was unadjusted. Model 2 was adjusted for sex, age at recruitment, body mass index (BMI), household income, educational attainment, ethnicity, Townsend deprivation index quartiles, physical activity (PA), alcohol consumption, and diet. Model 3 was additionally adjusted for candidate SNPs. “Ever” indicates any exposure versus never exposure (reference). Passive SHS exposure was defined as a composite measure of secondhand smoke exposure. Statistical significance is indicated as *P < 0.05, **P < 0.01, and ***P < 0.001.

**Supplementary Table S16. Joint associations of smoking-related exposures and IPF with all-cause and cause-specific mortality during follow-up among participants with complete covariate data**

| **Joint variables** | **Cases/N** | **HR (95% CI)** | | |
| --- | --- | --- | --- | --- |
|  |  | **Model 1** | **Model 2** | **Model 3** |
| **All-cause mortality** |  |  |  |  |
| Smoking status |  |  |  |  |
| Non-current * No IPF | 12406/193829 | 1.000 (ref) | 1.000 (ref) | 1.000 (ref) |
| Non-current * IPF | 363/714 | 9.977 (8.988–11.075)*** | 5.125 (4.613–5.693)*** | 5.125 (4.610–5.697)*** |
| Current * No IPF | 394/5879 | 1.049 (0.949–1.159) | 1.196 (1.081–1.322)*** | 1.196 (1.081–1.322)*** |
| Current * IPF | 16/31 | 8.931 (5.470–14.583)*** | 4.668 (2.857–7.625)*** | 4.659 (2.851–7.611)*** |
| Adult SHS exposure |  |  |  |  |
| Never * No IPF | 9797/156873 | 1.000 (ref) | 1.000 (ref) | 1.000 (ref) |
| Never * IPF | 264/531 | 9.889 (8.751–11.175)*** | 5.152 (4.556–5.826)*** | 5.149 (4.551–5.826)*** |
| Ever * No IPF | 3003/42835 | 1.121 (1.076–1.168)*** | 1.101 (1.056–1.148)*** | 1.101 (1.056–1.148)*** |
| Ever * IPF | 115/214 | 10.886 (9.057–13.083)*** | 5.304 (4.409–6.380)*** | 5.311 (4.413–6.391)*** |
| Maternal smoking exposure |  |  |  |  |
| Never * No IPF | 9153/142183 | 1.000 (ref) | 1.000 (ref) | 1.000 (ref) |
| Never * IPF | 253/492 | 9.932 (8.766–11.254)*** | 4.893 (4.315–5.549)*** | 4.891 (4.312–5.549)*** |
| Ever * No IPF | 3647/57525 | 0.982 (0.945–1.020) | 1.028 (0.989–1.069) | 1.028 (0.989–1.069) |
| Ever * IPF | 126/253 | 9.721 (8.154–11.590)*** | 5.635 (4.724–6.722)*** | 5.640 (4.726–6.732)*** |
| Passive SHS exposure |  |  |  |  |
| Never * No IPF | 6761/107295 | 1.000 (ref) | 1.000 (ref) | 1.000 (ref) |
| Never * IPF | 169/327 | 10.212 (8.766–11.896)*** | 5.236 (4.492–6.103)*** | 5.231 (4.486–6.099)*** |
| Ever * No IPF | 6039/92413 | 1.035 (0.999–1.071) | 1.074 (1.037–1.112)*** | 1.074 (1.036–1.112)*** |
| Ever * IPF | 210/418 | 9.964 (8.685–11.431)*** | 5.263 (4.583–6.043)*** | 5.266 (4.583–6.051)*** |
| **CVD mortality** |  |  |  |  |
| Smoking status |  |  |  |  |
| Non-current * No IPF | 2099/193829 | 1.000 (ref) | 1.000 (ref) | 1.000 (ref) |
| Non-current * IPF | 44/714 | 7.238 (5.370–9.758)*** | 2.932 (2.172–3.959)*** | 2.872 (2.125–3.883)*** |
| Current * No IPF | 91/5879 | 1.432 (1.161–1.766)*** | 1.545 (1.251–1.908)*** | 1.543 (1.249–1.905)*** |
| Current * IPF | 2/31 | 6.635 (1.658–26.547)** | 2.482 (0.620–9.938) | 2.426 (0.606–9.720) |
| Adult SHS exposure |  |  |  |  |
| Never * No IPF | 1615/156873 | 1.000 (ref) | 1.000 (ref) | 1.000 (ref) |
| Never * IPF | 37/531 | 8.514 (6.146–11.795)*** | 3.548 (2.558–4.920)*** | 3.481 (2.506–4.834)*** |
| Ever * No IPF | 575/42835 | 1.301 (1.183–1.431)*** | 1.163 (1.055–1.282)** | 1.163 (1.055–1.282)** |
| Ever * IPF | 9/214 | 5.224 (2.713–10.059)*** | 1.800 (0.934–3.472) | 1.756 (0.910–3.389) |
| Maternal smoking exposure |  |  |  |  |
| Never * No IPF | 1531/142183 | 1.000 (ref) | 1.000 (ref) | 1.000 (ref) |
| Never * IPF | 34/492 | 8.074 (5.748–11.343)*** | 3.073 (2.184–4.324)*** | 3.013 (2.140–4.244)*** |
| Ever * No IPF | 659/57525 | 1.060 (0.967–1.161) | 1.109 (1.011–1.217)* | 1.109 (1.011–1.217)* |
| Ever * IPF | 12/253 | 5.605 (3.176–9.892)*** | 2.661 (1.507–4.700)*** | 2.600 (1.470–4.599)** |
| Passive SHS exposure |  |  |  |  |
| Never * No IPF | 1112/107295 | 1.000 (ref) | 1.000 (ref) | 1.000 (ref) |
| Never * IPF | 26/327 | 9.667 (6.552–14.262)*** | 3.971 (2.689–5.866)*** | 3.896 (2.636–5.759)*** |
| Ever * No IPF | 1078/92413 | 1.122 (1.032–1.220)** | 1.112 (1.021–1.211)* | 1.112 (1.021–1.212)* |
| Ever * IPF | 20/418 | 5.839 (3.752–9.086)*** | 2.300 (1.476–3.586)*** | 2.249 (1.441–3.510)*** |
| **Cancer mortality** |  |  |  |  |
| Smoking status |  |  |  |  |
| Non-current * No IPF | 6907/193829 | 1.000 (ref) | 1.000 (ref) | 1.000 (ref) |
| Non-current * IPF | 91/714 | 4.377 (3.559–5.383)*** | 2.545 (2.068–3.133)*** | 2.540 (2.062–3.128)*** |
| Current * No IPF | 194/5879 | 0.928 (0.804–1.070) | 1.083 (0.939–1.251) | 1.084 (0.939–1.251) |
| Current * IPF | 4/31 | 3.961 (1.486–10.556)** | 2.434 (0.913–6.490) | 2.417 (0.907–6.446) |
| Adult SHS exposure |  |  |  |  |
| Never * No IPF | 5561/156873 | 1.000 (ref) | 1.000 (ref) | 1.000 (ref) |
| Never * IPF | 68/531 | 4.374 (3.443–5.555)*** | 2.571 (2.023–3.268)*** | 2.563 (2.015–3.259)*** |
| Ever * No IPF | 1540/42835 | 1.014 (0.958–1.073) | 1.038 (0.980–1.100) | 1.039 (0.981–1.100) |
| Ever * IPF | 27/214 | 4.395 (3.011–6.415)*** | 2.522 (1.727–3.684)*** | 2.522 (1.726–3.685)*** |
| Maternal smoking exposure |  |  |  |  |
| Never * No IPF | 5131/142183 | 1.000 (ref) | 1.000 (ref) | 1.000 (ref) |
| Never * IPF | 66/492 | 4.509 (3.537–5.748)*** | 2.541 (1.992–3.242)*** | 2.535 (1.986–3.236)*** |
| Ever * No IPF | 1970/57525 | 0.947 (0.899–0.997)* | 0.987 (0.937–1.040) | 0.987 (0.936–1.040) |
| Ever * IPF | 29/253 | 3.889 (2.700–5.602)*** | 2.491 (1.729–3.590)*** | 2.484 (1.723–3.582)*** |
| Passive SHS exposure |  |  |  |  |
| Never * No IPF | 3849/107295 | 1.000 (ref) | 1.000 (ref) | 1.000 (ref) |
| Never * IPF | 43/327 | 4.451 (3.296–6.012)*** | 2.583 (1.911–3.490)*** | 2.575 (1.905–3.481)*** |
| Ever * No IPF | 3252/92413 | 0.979 (0.935–1.026) | 1.030 (0.983–1.080) | 1.030 (0.982–1.080) |
| Ever * IPF | 52/418 | 4.227 (3.215–5.557)*** | 2.560 (1.945–3.368)*** | 2.554 (1.940–3.363)*** |
| **Respiratory mortality** |  |  |  |  |
| Smoking status |  |  |  |  |
| Non-current * No IPF | 369/193829 | 1.000 (ref) | 1.000 (ref) | 1.000 (ref) |
| Non-current * IPF | 180/714 | 169.172 (141.487–202.274)*** | 75.326 (62.310–91.060)*** | 74.888 (61.585–91.065)*** |
| Current * No IPF | 9/5879 | 0.806 (0.416–1.560) | 0.919 (0.473–1.784) | 0.918 (0.473–1.782) |
| Current * IPF | 9/31 | 170.646 (88.085–330.588)*** | 73.039 (37.411–142.599)*** | 72.871 (37.228–142.640)*** |
| Adult SHS exposure |  |  |  |  |
| Never * No IPF | 266/156873 | 1.000 (ref) | 1.000 (ref) | 1.000 (ref) |
| Never * IPF | 123/531 | 172.363 (139.139–213.522)*** | 78.040 (62.464–97.500)*** | 77.572 (61.766–97.423)*** |
| Ever * No IPF | 112/42835 | 1.538 (1.234–1.918)*** | 1.482 (1.184–1.855)*** | 1.481 (1.184–1.854)*** |
| Ever * IPF | 66/214 | 234.263 (178.863–306.823)*** | 98.042 (73.781–130.281)*** | 97.802 (73.171–130.724)*** |
| Maternal smoking exposure |  |  |  |  |
| Never * No IPF | 256/142183 | 1.000 (ref) | 1.000 (ref) | 1.000 (ref) |
| Never * IPF | 120/492 | 171.187 (137.764–212.719)*** | 72.168 (57.493–90.589)*** | 71.400 (56.650–89.990)*** |
| Ever * No IPF | 122/57525 | 1.174 (0.946–1.456) | 1.267 (1.019–1.575)* | 1.264 (1.017–1.572)* |
| Ever * IPF | 69/253 | 193.629 (148.372–252.691)*** | 101.241 (76.968–133.170)*** | 102.663 (77.523–135.955)*** |
| Passive SHS exposure |  |  |  |  |
| Never * No IPF | 172/107295 | 1.000 (ref) | 1.000 (ref) | 1.000 (ref) |
| Never * IPF | 79/327 | 190.792 (146.128–249.107)*** | 85.165 (64.753–112.010)*** | 84.426 (63.982–111.402)*** |
| Ever * No IPF | 206/92413 | 1.386 (1.132–1.697)** | 1.462 (1.191–1.794)*** | 1.460 (1.189–1.793)*** |
| Ever * IPF | 110/418 | 208.451 (164.033–264.899)*** | 96.222 (74.761–123.844)*** | 96.087 (74.203–124.426)*** |

Abbreviations: Cases/N, number of deaths/total number of participants; HR, hazard ratio; CI, confidence interval; CVD, cardiovascular disease; BMI, body mass index; PA, physical activity; SHS, secondhand smoke; SNP, single nucleotide polymorphism. “ref” indicates the reference category.

Hazard ratios (HRs) and 95% confidence intervals (CIs) were estimated using Cox proportional hazards regression. Model 1 was unadjusted. Model 2 was adjusted for sex, age at recruitment, body mass index (BMI), household income, educational attainment, ethnicity, Townsend deprivation index quartiles, physical activity (PA), alcohol consumption, and diet. Model 3 was additionally adjusted for candidate SNPs. “Ever” indicates any exposure versus never exposure (reference). For smoking status, the exposed category was current smoking and the reference category was non-current smoking, which included never and former smokers. For passive smoking exposures, exposed participants were compared with unexposed participants. Statistical significance is indicated as *P < 0.05, **P < 0.01, and ***P < 0.001.

**Supplementary Table S17. Additive interactions between smoking-related exposures and IPF in relation to all-cause and cause-specific mortality during follow-up among participants with complete covariate data**

| **Additive interaction** | **Index for additive interaction** | **All-cause mortality** | **CVD mortality** | **Cancer mortality** | **Respiratory mortality** |
| --- | --- | --- | --- | --- | --- |
| Smoking status | RERI | -0.662 (-3.009–1.685) | -0.989 (-4.473–2.496) | -0.206 (-2.637–2.225) | -1.936 (-51.146–47.275) |
|  | AP | -0.142 (-0.714–0.430) | -0.407 (-2.394–1.579) | -0.085 (-1.173–1.002) | -0.027 (-0.719–0.666) |
|  | SI | 0.847 (0.448–1.601) | 0.591 (0.054–6.434) | 0.873 (0.159–4.802) | 0.974 (0.491–1.929) |
| Adult SHS exposure | RERI | 0.060 (-1.094–1.215) | **−1.888 (−3.504 to −0.272)** | -0.080 (-1.211–1.052) | 19.749 (-8.470–47.968) |
|  | AP | 0.011 (-0.204–0.227) | -1.075 (-2.577–0.427) | -0.032 (-0.490–0.427) | 0.202 (-0.042–0.446) |
|  | SI | 1.014 (0.775–1.327) | 0.286 (0.059–1.387) | 0.950 (0.457–1.978) | 1.256 (0.922–1.711) |
| Maternal smoking exposure | RERI | 0.721 (-0.435–1.877) | -0.522 (-2.314–1.269) | -0.037 (-1.130–1.056) | **30.999 (2.809–59.190)** |
|  | AP | 0.128 (-0.059–0.314) | -0.201 (-0.986–0.584) | -0.015 (-0.460–0.430) | 0.302 (0.092–0.511) |
|  | SI | 1.184 (0.911–1.539) | 0.754 (0.267–2.128) | 0.976 (0.470–2.026) | 1.439 (1.062–1.949) |
| Passive SHS exposure | RERI | -0.039 (-1.103–1.026) | -1.760 (-3.564–0.044) | -0.051 (-1.089–0.987) | 11.201 (-15.432–37.835) |
|  | AP | -0.007 (-0.210–0.196) | -0.783 (-1.812–0.246) | -0.020 (-0.430–0.390) | 0.117 (-0.144–0.377) |
|  | SI | 0.991 (0.773–1.270) | 0.415 (0.163–1.058) | 0.968 (0.502–1.866) | 1.134 (0.841–1.528) |

Abbreviations: RERI, relative excess risk due to interaction; AP, attributable proportion; SI, synergy index; CI, confidence interval; CVD, cardiovascular disease; BMI, body mass index; PA, physical activity; SHS, secondhand smoke; SNP, single nucleotide polymorphism.

Additive interactions between smoking-related exposures and IPF in relation to all-cause and cause-specific mortality during follow-up among participants with complete covariate data were evaluated using RERI, AP, and SI with 95% CIs from Model 3. Model 3 was adjusted for sex, age at recruitment, body mass index (BMI), household income, educational attainment, ethnicity, Townsend deprivation index quartiles, physical activity (PA), alcohol consumption, diet, and candidate SNPs. “Ever” indicates any exposure versus never exposure (reference). For smoking status, the exposed category was current smoking and the reference category was non-current smoking, which included never and former smokers. For passive smoking exposures, exposed participants were compared with unexposed participants. Boldface indicates RERI estimates whose 95% CIs exclude zero.

**Supplementary Table S18. Multiplicative interactions between smoking-related exposures and IPF in relation to all-cause and cause-specific mortality during follow-up among participants with complete covariate data**

| **interaction** | **HR (95% CI)** | | | |
| --- | --- | --- | --- | --- |
|  | **All-cause mortality** | **CVD mortality** | **Cancer mortality** | **Respiratory mortality** |
| Smoking status | 0.760 (0.456–1.267) | 0.548 (0.131–2.295) | 0.878 (0.319–2.415) | 1.060 (0.413–2.723) |
| Adult SHS exposure | 0.937 (0.749–1.171) | 0.434 (0.208–0.905)* | 0.947 (0.604–1.485) | 0.851 (0.584–1.240) |
| Maternal smoking exposure | 1.121 (0.902–1.394) | 0.778 (0.400–1.512) | 0.993 (0.640–1.542) | 1.138 (0.787–1.645) |
| Passive SHS exposure | 0.938 (0.763–1.152) | 0.519 (0.288–0.936)* | 0.963 (0.641–1.446) | 0.779 (0.545–1.114) |

Abbreviations: HR, hazard ratio; CI, confidence interval; CVD, cardiovascular disease; BMI, body mass index; PA, physical activity; SHS, secondhand smoke; SNP, single nucleotide polymorphism.

Multiplicative interactions between smoking-related exposures and IPF in relation to all-cause and cause-specific mortality during follow-up among participants with complete covariate data were evaluated using the HR and 95% CI for the interaction term from Model 3. Model 3 was adjusted for sex, age at recruitment, body mass index (BMI), household income, educational attainment, ethnicity, Townsend deprivation index quartiles, physical activity (PA), alcohol consumption, diet, and candidate SNPs. “Ever” indicates any exposure versus never exposure (reference). For smoking status, the exposed category was current smoking and the reference category was non-current smoking, which included never and former smokers. For passive smoking exposures, exposed participants were compared with unexposed participants. Statistical significance is indicated as *P < 0.05, **P < 0.01, and ***P < 0.001.

**Supplementary Table S19. Independent associations of smoking-related exposures and IPF with all-cause and cause-specific mortality during follow-up after excluding events within the first two years of follow-up**

| **Exposure** | **Person-years**  **(PYs)** | **Cases/N** | **Incidence Rate**  **(95% CI)** | **HR (95% CI)** | | |
| --- | --- | --- | --- | --- | --- | --- |
|  |  |  |  | **Model 1** | **Model 2** | **Model 3** |
| **All-cause mortality** |  |  |  |  |  |  |
| **IPF** |  |  |  |  |  |  |
| No | 3628437.86 | 19143/268179 | 5.276 (5.201–5.351) | 1.000 (ref) | 1.000 (ref) | 1.000 (ref) |
| Yes | 15558.12 | 659/1308 | 42.357 (39.123–45.591) | 8.706 (8.055–9.409)*** | 4.430 (4.096–4.791)*** | 4.420 (4.085–4.783)*** |
| **Smoking status** |  |  |  |  |  |  |
| Never | 2182062.39 | 9506/160528 | 4.356 (4.269–4.444) | 1.000 (ref) | 1.000 (ref) | 1.000 (ref) |
| Former | 1358835.13 | 9658/101318 | 7.108 (6.966–7.249) | 1.643 (1.597–1.690)*** | 1.249 (1.213–1.286)*** | 1.249 (1.213–1.286)*** |
| Current | 103098.46 | 638/7641 | 6.188 (5.708–6.668) | 1.425 (1.315–1.544)*** | 1.453 (1.340–1.575)*** | 1.453 (1.340–1.575)*** |
| **Adult SHS exposure** |  |  |  |  |  |  |
| Never | 2838221.54 | 14852/209858 | 5.233 (5.149–5.317) | 1.000 (ref) | 1.000 (ref) | 1.000 (ref) |
| Ever | 805774.44 | 4950/59629 | 6.143 (5.972–6.314) | 1.173 (1.136–1.211)*** | 1.124 (1.087–1.161)*** | 1.124 (1.087–1.161)*** |
| **Maternal smoking exposure** |  |  |  |  |  |  |
| Never | 2576961.29 | 14018/190666 | 5.440 (5.350–5.530) | 1.000 (ref) | 1.000 (ref) | 1.000 (ref) |
| Ever | 1067034.70 | 5784/78821 | 5.421 (5.281–5.560) | 0.995 (0.965–1.026) | 1.040 (1.009–1.073)* | 1.040 (1.008–1.073)* |
| **Passive SHS exposure** |  |  |  |  |  |  |
| Never | 1930002.98 | 10164/142764 | 5.266 (5.164–5.369) | 1.000 (ref) | 1.000 (ref) | 1.000 (ref) |
| Ever | 1713993.00 | 9638/126723 | 5.623 (5.511–5.735) | 1.066 (1.037–1.096)*** | 1.093 (1.062–1.125)*** | 1.093 (1.062–1.124)*** |
| **Pack years** |  |  |  |  |  |  |
| 0 | 2187401.10 | 9574/160937 | 4.377 (4.289–4.465) | 1.000 (ref) | 1.000 (ref) | 1.000 (ref) |
| 1–10 | 269728.57 | 1193/19867 | 4.423 (4.172–4.674) | 1.012 (0.953–1.075) | 0.973 (0.916–1.034) | 0.973 (0.916–1.034) |
| 11–20 | 258937.81 | 1555/19205 | 6.005 (5.707–6.304) | 1.378 (1.306–1.454)*** | 1.124 (1.064–1.186)*** | 1.124 (1.065–1.186)*** |
| 21–30 | 164437.25 | 1245/12260 | 7.571 (7.151–7.992) | 1.741 (1.641–1.847)*** | 1.253 (1.180–1.330)*** | 1.252 (1.180–1.330)*** |
| >30 | 198417.02 | 2954/15337 | 14.888 (14.351–15.425) | 3.484 (3.344–3.631)*** | 1.911 (1.829–1.996)*** | 1.910 (1.829–1.996)*** |
| Unknown | 565074.25 | 3281/41881 | 5.806 (5.608–6.005) | 1.333 (1.281–1.387)*** | 1.135 (1.091–1.182)*** | 1.136 (1.091–1.182)*** |
| **CVD mortality** |  |  |  |  |  |  |
| **IPF** |  |  |  |  |  |  |
| No | 3628437.86 | 3437/268179 | 0.947 (0.916–0.979) | 1.000 (ref) | 1.000 (ref) | 1.000 (ref) |
| Yes | 15558.12 | 82/1308 | 5.271 (4.130–6.411) | 6.095 (4.896–7.588)*** | 2.546 (2.043–3.173)*** | 2.494 (1.999–3.112)*** |
| **Smoking status** |  |  |  |  |  |  |
| Never | 2182062.39 | 1595/160528 | 0.731 (0.695–0.767) | 1.000 (ref) | 1.000 (ref) | 1.000 (ref) |
| Former | 1358835.13 | 1799/101318 | 1.324 (1.263–1.385) | 1.826 (1.707–1.953)*** | 1.245 (1.161–1.335)*** | 1.245 (1.161–1.335)*** |
| Current | 103098.46 | 125/7641 | 1.212 (1.000–1.425) | 1.665 (1.387–1.997)*** | 1.546 (1.287–1.857)*** | 1.545 (1.286–1.856)*** |
| **Adult SHS exposure** |  |  |  |  |  |  |
| Never | 2838221.54 | 2554/209858 | 0.900 (0.865–0.935) | 1.000 (ref) | 1.000 (ref) | 1.000 (ref) |
| Ever | 805774.44 | 965/59629 | 1.198 (1.122–1.273) | 1.328 (1.234–1.431)*** | 1.173 (1.087–1.265)*** | 1.172 (1.087–1.265)*** |
| **Maternal smoking exposure** |  |  |  |  |  |  |
| Never | 2576961.29 | 2474/190666 | 0.960 (0.922–0.998) | 1.000 (ref) | 1.000 (ref) | 1.000 (ref) |
| Ever | 1067034.70 | 1045/78821 | 0.979 (0.920–1.039) | 1.018 (0.947–1.094) | 1.063 (0.988–1.144) | 1.062 (0.987–1.143) |
| **Passive SHS exposure** |  |  |  |  |  |  |
| Never | 1930002.98 | 1753/142764 | 0.908 (0.866–0.951) | 1.000 (ref) | 1.000 (ref) | 1.000 (ref) |
| Ever | 1713993.00 | 1766/126723 | 1.030 (0.982–1.078) | 1.132 (1.060–1.209)*** | 1.115 (1.042–1.192)** | 1.114 (1.041–1.192)** |
| **Pack years** |  |  |  |  |  |  |
| 0 | 2187401.10 | 1605/160937 | 0.734 (0.698–0.770) | 1.000 (ref) | 1.000 (ref) | 1.000 (ref) |
| 1–10 | 269728.57 | 177/19867 | 0.656 (0.560–0.753) | 0.896 (0.767–1.046) | 0.860 (0.736–1.005) | 0.861 (0.737–1.006) |
| 11–20 | 258937.81 | 265/19205 | 1.023 (0.900–1.147) | 1.402 (1.231–1.597)*** | 1.050 (0.921–1.197) | 1.050 (0.921–1.198) |
| 21–30 | 164437.25 | 255/12260 | 1.551 (1.360–1.741) | 2.128 (1.865–2.429)*** | 1.338 (1.170–1.530)*** | 1.337 (1.170–1.529)*** |
| >30 | 198417.02 | 585/15337 | 2.948 (2.709–3.187) | 4.127 (3.754–4.536)*** | 1.786 (1.616–1.974)*** | 1.787 (1.616–1.975)*** |
| Unknown | 565074.25 | 632/41881 | 1.118 (1.031–1.206) | 1.532 (1.398–1.680)*** | 1.210 (1.102–1.328)*** | 1.211 (1.103–1.329)*** |
| **Cancer mortality** |  |  |  |  |  |  |
| **IPF** |  |  |  |  |  |  |
| No | 3628437.86 | 10024/268179 | 2.763 (2.709–2.817) | 1.000 (ref) | 1.000 (ref) | 1.000 (ref) |
| Yes | 15558.12 | 151/1308 | 9.706 (8.157–11.254) | 3.711 (3.160–4.358)*** | 2.152 (1.831–2.529)*** | 2.150 (1.829–2.528)*** |
| **Smoking status** |  |  |  |  |  |  |
| Never | 2182062.39 | 5018/160528 | 2.300 (2.236–2.363) | 1.000 (ref) | 1.000 (ref) | 1.000 (ref) |
| Former | 1358835.13 | 4845/101318 | 3.566 (3.465–3.666) | 1.558 (1.498–1.621)*** | 1.263 (1.212–1.316)*** | 1.263 (1.212–1.316)*** |
| Current | 103098.46 | 312/7641 | 3.026 (2.690–3.362) | 1.319 (1.177–1.479)*** | 1.409 (1.256–1.582)*** | 1.410 (1.256–1.582)*** |
| **Adult SHS exposure** |  |  |  |  |  |  |
| Never | 2838221.54 | 7826/209858 | 2.757 (2.696–2.818) | 1.000 (ref) | 1.000 (ref) | 1.000 (ref) |
| Ever | 805774.44 | 2349/59629 | 2.915 (2.797–3.033) | 1.057 (1.009–1.107)* | 1.063 (1.014–1.115)* | 1.063 (1.015–1.115)* |
| **Maternal smoking exposure** |  |  |  |  |  |  |
| Never | 2576961.29 | 7240/190666 | 2.810 (2.745–2.874) | 1.000 (ref) | 1.000 (ref) | 1.000 (ref) |
| Ever | 1067034.70 | 2935/78821 | 2.751 (2.651–2.850) | 0.978 (0.937–1.021) | 1.017 (0.974–1.063) | 1.017 (0.974–1.062) |
| **Passive SHS exposure** |  |  |  |  |  |  |
| Never | 1930002.98 | 5358/142764 | 2.776 (2.702–2.850) | 1.000 (ref) | 1.000 (ref) | 1.000 (ref) |
| Ever | 1713993.00 | 4817/126723 | 2.810 (2.731–2.890) | 1.012 (0.973–1.052) | 1.055 (1.014–1.098)** | 1.055 (1.014–1.097)** |
| **Pack years** |  |  |  |  |  |  |
| 0 | 2187401.10 | 5048/160937 | 2.308 (2.244–2.371) | 1.000 (ref) | 1.000 (ref) | 1.000 (ref) |
| 1–10 | 269728.57 | 695/19867 | 2.577 (2.385–2.768) | 1.117 (1.032–1.210)** | 1.073 (0.990–1.162) | 1.073 (0.991–1.162) |
| 11–20 | 258937.81 | 822/19205 | 3.175 (2.957–3.392) | 1.380 (1.282–1.485)*** | 1.184 (1.099–1.276)*** | 1.184 (1.099–1.276)*** |
| 21–30 | 164437.25 | 617/12260 | 3.752 (3.456–4.048) | 1.633 (1.502–1.776)*** | 1.282 (1.178–1.395)*** | 1.282 (1.178–1.395)*** |
| >30 | 198417.02 | 1413/15337 | 7.121 (6.750–7.493) | 3.140 (2.960–3.331)*** | 2.022 (1.899–2.153)*** | 2.022 (1.899–2.152)*** |
| Unknown | 565074.25 | 1580/41881 | 2.796 (2.658–2.934) | 1.215 (1.149–1.286)*** | 1.079 (1.019–1.142)** | 1.079 (1.019–1.142)** |
| **Respiratory mortality** |  |  |  |  |  |  |
| **IPF** |  |  |  |  |  |  |
| No | 3628437.86 | 701/268179 | 0.193 (0.179–0.207) | 1.000 (ref) | 1.000 (ref) | 1.000 (ref) |
| Yes | 15558.12 | 340/1308 | 21.854 (19.531–24.176) | 124.753 (109.557–142.056)*** | 53.418 (46.573–61.270)*** | 52.338 (45.421–60.308)*** |
| **Smoking status** |  |  |  |  |  |  |
| Never | 2182062.39 | 344/160528 | 0.158 (0.141–0.174) | 1.000 (ref) | 1.000 (ref) | 1.000 (ref) |
| Former | 1358835.13 | 654/101318 | 0.481 (0.444–0.518) | 3.078 (2.701–3.507)*** | 2.059 (1.799–2.356)*** | 2.054 (1.794–2.351)*** |
| Current | 103098.46 | 43/7641 | 0.417 (0.292–0.542) | 2.656 (1.935–3.647)*** | 2.528 (1.835–3.481)*** | 2.508 (1.821–3.454)*** |
| **Adult SHS exposure** |  |  |  |  |  |  |
| Never | 2838221.54 | 725/209858 | 0.255 (0.237–0.274) | 1.000 (ref) | 1.000 (ref) | 1.000 (ref) |
| Ever | 805774.44 | 316/59629 | 0.392 (0.349–0.435) | 1.534 (1.344–1.750)*** | 1.349 (1.178–1.544)*** | 1.349 (1.179–1.545)*** |
| **Maternal smoking exposure** |  |  |  |  |  |  |
| Never | 2576961.29 | 694/190666 | 0.269 (0.249–0.289) | 1.000 (ref) | 1.000 (ref) | 1.000 (ref) |
| Ever | 1067034.70 | 347/78821 | 0.325 (0.291–0.359) | 1.205 (1.059–1.371)** | 1.282 (1.124–1.461)*** | 1.275 (1.118–1.453)*** |
| **Passive SHS exposure** |  |  |  |  |  |  |
| Never | 1930002.98 | 463/142764 | 0.240 (0.218–0.262) | 1.000 (ref) | 1.000 (ref) | 1.000 (ref) |
| Ever | 1713993.00 | 578/126723 | 0.337 (0.310–0.365) | 1.404 (1.242–1.586)*** | 1.403 (1.238–1.589)*** | 1.398 (1.234–1.584)*** |
| **Pack years** |  |  |  |  |  |  |
| 0 | 2187401.10 | 353/160937 | 0.161 (0.145–0.178) | 1.000 (ref) | 1.000 (ref) | 1.000 (ref) |
| 1–10 | 269728.57 | 46/19867 | 0.171 (0.121–0.220) | 1.059 (0.779–1.439) | 1.029 (0.756–1.400) | 1.026 (0.754–1.396) |
| 11–20 | 258937.81 | 83/19205 | 0.321 (0.252–0.390) | 1.996 (1.572–2.536)*** | 1.523 (1.196–1.939)*** | 1.525 (1.198–1.942)*** |
| 21–30 | 164437.25 | 90/12260 | 0.547 (0.434–0.660) | 3.415 (2.710–4.305)*** | 2.183 (1.725–2.761)*** | 2.168 (1.714–2.743)*** |
| >30 | 198417.02 | 313/15337 | 1.577 (1.403–1.752) | 10.046 (8.628–11.697)*** | 4.316 (3.661–5.087)*** | 4.290 (3.640–5.057)*** |
| Unknown | 565074.25 | 156/41881 | 0.276 (0.233–0.319) | 1.720 (1.424–2.076)*** | 1.391 (1.150–1.683)*** | 1.390 (1.149–1.682)*** |

Abbreviations: PYs, person-years; Cases/N, number of deaths/total number of participants; HR, hazard ratio; CI, confidence interval; CVD, cardiovascular disease; BMI, body mass index; PA, physical activity; SHS, secondhand smoke; SNP, single nucleotide polymorphism. Incidence rates are presented per 1,000 person-years, and “ref” indicates the reference category.

Hazard ratios (HRs) and 95% confidence intervals (CIs) were estimated using Cox proportional hazards regression. Model 1 was unadjusted. Model 2 was adjusted for sex, age at recruitment, body mass index (BMI), household income, educational attainment, ethnicity, Townsend deprivation index quartiles, physical activity (PA), alcohol consumption, and diet. Model 3 was additionally adjusted for candidate SNPs. “Ever” indicates any exposure versus never exposure (reference). Passive SHS exposure was defined as a composite measure of secondhand smoke exposure. Statistical significance is indicated as *P < 0.05, **P < 0.01, and ***P < 0.001.

**Supplementary Table S20. Joint associations of smoking-related exposures and IPF with all-cause and cause-specific mortality during follow-up after excluding events within the first two years of follow-up**

| **Joint variables** | **Cases/N** | **HR (95% CI)** | | |
| --- | --- | --- | --- | --- |
|  |  | **Model 1** | **Model 2** | **Model 3** |
| **All-cause mortality** |  |  |  |  |
| Smoking status |  |  |  |  |
| Non-current * No IPF | 18536/260593 | 1.000 (ref) | 1.000 (ref) | 1.000 (ref) |
| Non-current * IPF | 628/1253 | 8.698 (8.033–9.418)*** | 4.425 (4.084–4.795)*** | 4.416 (4.073–4.787)*** |
| Current * No IPF | 607/7586 | 1.128 (1.040–1.223)** | 1.281 (1.181–1.390)*** | 1.282 (1.182–1.390)*** |
| Current * IPF | 31/55 | 9.616 (6.761–13.677)*** | 5.335 (3.750–7.590)*** | 5.327 (3.744–7.579)*** |
| Adult SHS exposure |  |  |  |  |
| Never * No IPF | 14398/208925 | 1.000 (ref) | 1.000 (ref) | 1.000 (ref) |
| Never * IPF | 454/933 | 8.562 (7.798–9.400)*** | 4.404 (4.009–4.838)*** | 4.395 (3.998–4.830)*** |
| Ever * No IPF | 4745/59254 | 1.161 (1.124–1.200)*** | 1.117 (1.081–1.155)*** | 1.117 (1.081–1.155)*** |
| Ever * IPF | 205/375 | 10.216 (8.900–11.726)*** | 4.945 (4.305–5.681)*** | 4.936 (4.296–5.672)*** |
| Maternal smoking exposure |  |  |  |  |
| Never * No IPF | 13571/189785 | 1.000 (ref) | 1.000 (ref) | 1.000 (ref) |
| Never * IPF | 447/881 | 8.731 (7.946–9.594)*** | 4.329 (3.937–4.760)*** | 4.322 (3.930–4.754)*** |
| Ever * No IPF | 5572/78394 | 0.990 (0.960–1.022) | 1.036 (1.004–1.070)* | 1.036 (1.004–1.069)* |
| Ever * IPF | 212/427 | 8.578 (7.490–9.824)*** | 4.820 (4.206–5.523)*** | 4.806 (4.192–5.509)*** |
| Passive SHS exposure |  |  |  |  |
| Never * No IPF | 9867/142164 | 1.000 (ref) | 1.000 (ref) | 1.000 (ref) |
| Never * IPF | 297/600 | 8.621 (7.681–9.676)*** | 4.350 (3.874–4.885)*** | 4.344 (3.867–4.879)*** |
| Ever * No IPF | 9276/126015 | 1.058 (1.029–1.089)*** | 1.087 (1.056–1.119)*** | 1.087 (1.056–1.119)*** |
| Ever * IPF | 362/708 | 9.228 (8.309–10.249)*** | 4.857 (4.370–5.398)*** | 4.845 (4.358–5.388)*** |
| **CVD mortality** |  |  |  |  |
| Smoking status |  |  |  |  |
| Non-current * No IPF | 3314/260593 | 1.000 (ref) | 1.000 (ref) | 1.000 (ref) |
| Non-current * IPF | 80/1253 | 6.261 (5.016–7.816)*** | 2.613 (2.091–3.266)*** | 2.559 (2.045–3.202)*** |
| Current * No IPF | 123/7586 | 1.278 (1.067–1.530)** | 1.394 (1.163–1.670)*** | 1.393 (1.162–1.669)*** |
| Current * IPF | 2/55 | 3.499 (0.875–13.995) | 1.577 (0.394–6.310) | 1.557 (0.389–6.233) |
| Adult SHS exposure |  |  |  |  |
| Never * No IPF | 2494/208925 | 1.000 (ref) | 1.000 (ref) | 1.000 (ref) |
| Never * IPF | 60/933 | 6.598 (5.107–8.524)*** | 2.826 (2.186–3.655)*** | 2.770 (2.140–3.585)*** |
| Ever * No IPF | 943/59254 | 1.331 (1.235–1.435)*** | 1.179 (1.092–1.273)*** | 1.179 (1.092–1.273)*** |
| Ever * IPF | 22/375 | 6.391 (4.200–9.724)*** | 2.306 (1.513–3.514)*** | 2.257 (1.480–3.441)*** |
| Maternal smoking exposure |  |  |  |  |
| Never * No IPF | 2414/189785 | 1.000 (ref) | 1.000 (ref) | 1.000 (ref) |
| Never * IPF | 60/881 | 6.655 (5.151–8.598)*** | 2.691 (2.080–3.480)*** | 2.638 (2.038–3.415)*** |
| Ever * No IPF | 1023/78394 | 1.022 (0.950–1.099) | 1.067 (0.991–1.150) | 1.067 (0.991–1.149) |
| Ever * IPF | 22/427 | 5.055 (3.322–7.691)*** | 2.367 (1.555–3.605)*** | 2.314 (1.518–3.527)*** |
| Passive SHS exposure |  |  |  |  |
| Never * No IPF | 1711/142164 | 1.000 (ref) | 1.000 (ref) | 1.000 (ref) |
| Never * IPF | 42/600 | 7.098 (5.226–9.641)*** | 2.988 (2.198–4.062)*** | 2.932 (2.155–3.988)*** |
| Ever * No IPF | 1726/126015 | 1.135 (1.061–1.213)*** | 1.120 (1.046–1.199)** | 1.119 (1.045–1.198)** |
| Ever * IPF | 40/708 | 5.939 (4.341–8.126)*** | 2.443 (1.783–3.347)*** | 2.389 (1.742–3.276)*** |
| **Cancer mortality** |  |  |  |  |
| Smoking status |  |  |  |  |
| Non-current * No IPF | 9722/260593 | 1.000 (ref) | 1.000 (ref) | 1.000 (ref) |
| Non-current * IPF | 141/1253 | 3.626 (3.071–4.282)*** | 2.104 (1.780–2.486)*** | 2.102 (1.778–2.485)*** |
| Current * No IPF | 302/7586 | 1.070 (0.954–1.200) | 1.237 (1.103–1.388)*** | 1.237 (1.103–1.388)*** |
| Current * IPF | 10/55 | 5.776 (3.107–10.739)*** | 3.675 (1.976–6.835)*** | 3.671 (1.974–6.828)*** |
| Adult SHS exposure |  |  |  |  |
| Never * No IPF | 7721/208925 | 1.000 (ref) | 1.000 (ref) | 1.000 (ref) |
| Never * IPF | 105/933 | 3.600 (2.970–4.365)*** | 2.098 (1.730–2.546)*** | 2.097 (1.728–2.545)*** |
| Ever * No IPF | 2303/59254 | 1.052 (1.004–1.102)* | 1.060 (1.011–1.112)* | 1.060 (1.011–1.112)* |
| Ever * IPF | 46/375 | 4.156 (3.110–5.554)*** | 2.407 (1.800–3.218)*** | 2.404 (1.798–3.216)*** |
| Maternal smoking exposure |  |  |  |  |
| Never * No IPF | 7138/189785 | 1.000 (ref) | 1.000 (ref) | 1.000 (ref) |
| Never * IPF | 102/881 | 3.691 (3.036–4.488)*** | 2.100 (1.726–2.554)*** | 2.099 (1.724–2.554)*** |
| Ever * No IPF | 2886/78394 | 0.976 (0.935–1.019) | 1.016 (0.972–1.061) | 1.015 (0.972–1.061) |
| Ever * IPF | 49/427 | 3.671 (2.772–4.862)*** | 2.305 (1.739–3.054)*** | 2.299 (1.735–3.047)*** |
| Passive SHS exposure |  |  |  |  |
| Never * No IPF | 5292/142164 | 1.000 (ref) | 1.000 (ref) | 1.000 (ref) |
| Never * IPF | 66/600 | 3.484 (2.733–4.442)*** | 2.001 (1.569–2.552)*** | 2.001 (1.568–2.552)*** |
| Ever * No IPF | 4732/126015 | 1.007 (0.968–1.048) | 1.051 (1.010–1.094)* | 1.051 (1.010–1.094)* |
| Ever * IPF | 85/708 | 3.933 (3.174–4.873)*** | 2.395 (1.931–2.969)*** | 2.390 (1.927–2.965)*** |
| **Respiratory mortality** |  |  |  |  |
| Smoking status |  |  |  |  |
| Non-current * No IPF | 676/260593 | 1.000 (ref) | 1.000 (ref) | 1.000 (ref) |
| Non-current * IPF | 322/1253 | 124.378 (108.881–142.080)*** | 53.207 (46.248–61.212)*** | 52.153 (45.121–60.280)*** |
| Current * No IPF | 25/7586 | 1.274 (0.855–1.899) | 1.417 (0.950–2.116) | 1.416 (0.949–2.114) |
| Current * IPF | 18/55 | 155.654 (97.463–248.588)*** | 72.952 (45.501–116.964)*** | 70.027 (43.632–112.389)*** |
| Adult SHS exposure |  |  |  |  |
| Never * No IPF | 494/208925 | 1.000 (ref) | 1.000 (ref) | 1.000 (ref) |
| Never * IPF | 231/933 | 128.940 (110.260–150.785)*** | 55.813 (47.452–65.648)*** | 54.608 (46.240–64.491)*** |
| Ever * No IPF | 207/59254 | 1.476 (1.255–1.736)*** | 1.344 (1.140–1.586)*** | 1.347 (1.142–1.589)*** |
| Ever * IPF | 109/375 | 161.613 (131.309–198.912)*** | 63.653 (51.224–79.098)*** | 62.719 (50.310–78.189)*** |
| Maternal smoking exposure |  |  |  |  |
| Never * No IPF | 471/189785 | 1.000 (ref) | 1.000 (ref) | 1.000 (ref) |
| Never * IPF | 223/881 | 127.606 (108.783–149.687)*** | 52.763 (44.694–62.289)*** | 51.548 (43.510–61.071)*** |
| Ever * No IPF | 230/78394 | 1.177 (1.005–1.378)* | 1.263 (1.077–1.482)** | 1.260 (1.074–1.478)** |
| Ever * IPF | 117/427 | 138.730 (113.277–169.902)*** | 68.690 (55.776–84.594)*** | 67.921 (54.911–84.013)*** |
| Passive SHS exposure |  |  |  |  |
| Never * No IPF | 312/142164 | 1.000 (ref) | 1.000 (ref) | 1.000 (ref) |
| Never * IPF | 151/600 | 140.748 (115.868–170.969)*** | 59.071 (48.380–72.125)*** | 57.779 (47.184–70.753)*** |
| Ever * No IPF | 389/126015 | 1.403 (1.209–1.628)*** | 1.431 (1.230–1.664)*** | 1.430 (1.230–1.664)*** |
| Ever * IPF | 189/708 | 155.114 (129.443–185.877)*** | 69.393 (57.446–83.825)*** | 68.248 (56.261–82.788)*** |

Abbreviations: PYs, person-years; Cases/N, number of deaths/total number of participants; HR, hazard ratio; CI, confidence interval; CVD, cardiovascular disease; BMI, body mass index; PA, physical activity; SHS, secondhand smoke; SNP, single nucleotide polymorphism. Incidence rates are presented per 1,000 person-years, and “ref” indicates the reference category.

Hazard ratios (HRs) and 95% confidence intervals (CIs) were estimated using Cox proportional hazards regression. Model 1 was unadjusted. Model 2 was adjusted for sex, age at recruitment, body mass index (BMI), household income, educational attainment, ethnicity, Townsend deprivation index quartiles, physical activity (PA), alcohol consumption, and diet. Model 3 was additionally adjusted for candidate SNPs. “Ever” indicates any exposure versus never exposure (reference). For smoking status, the exposed category was current smoking and the reference category was non-current smoking, which included never and former smokers. For passive smoking exposures, exposed participants were compared with unexposed participants. Pack-years were categorized as 0, 1–10, 11–20, 21–30, and >30; “Unknown” indicates missing pack-years information. Statistical significance is indicated as *P < 0.05, **P < 0.01, and ***P < 0.001.

**Supplementary Table S21. Additive interactions between smoking-related exposures and IPF in relation to all-cause and cause-specific mortality during follow-up after excluding events within the first two years of follow-up**

| **Additive interaction** | **Index for additive interaction** | **All-cause mortality** | **CVD mortality** | **Cancer mortality** | **Respiratory mortality** |
| --- | --- | --- | --- | --- | --- |
| Smoking status | RERI | 0.629 (-1.281–2.539) | -1.394 (-3.640–0.851) | 1.332 (-0.974–3.638) | 17.458 (-15.603–50.519) |
|  | AP | 0.118 (-0.200–0.436) | -0.895 (-3.552–1.761) | 0.363 (-0.045–0.771) | 0.249 (-0.109–0.608) |
|  | SI | 1.170 (0.750–1.825) | 0.286 (0.006–13.910) | 1.995 (0.813–4.891) | 1.339 (0.825–2.173) |
| Adult SHS exposure | RERI | 0.424 (-0.365–1.213) | -0.692 (-1.872–0.488) | 0.248 (-0.556–1.051) | 7.764 (-6.236–21.764) |
|  | AP | 0.086 (-0.064–0.236) | -0.307 (-0.936–0.323) | 0.103 (-0.206–0.412) | 0.124 (-0.079–0.326) |
|  | SI | 1.121 (0.911–1.379) | 0.645 (0.280–1.485) | 1.214 (0.662–2.225) | 1.144 (0.904–1.447) |
| Maternal smoking exposure | RERI | 0.447 (-0.315–1.209) | -0.390 (-1.569–0.788) | 0.185 (-0.578–0.948) | **16.113 (1.811–30.415)** |
|  | AP | 0.093 (-0.055–0.241) | -0.169 (-0.737–0.400) | 0.081 (-0.233–0.394) | 0.237 (0.065–0.409) |
|  | SI | 1.133 (0.920–1.395) | 0.771 (0.335–1.777) | 1.166 (0.629–2.162) | 1.317 (1.047–1.657) |
| Passive SHS exposure | RERI | 0.415 (-0.289–1.118) | -0.662 (-1.822–0.498) | 0.339 (-0.363–1.041) | 10.038 (-3.650–23.727) |
|  | AP | 0.086 (-0.053–0.225) | -0.277 (-0.821–0.267) | 0.142 (-0.131–0.414) | 0.147 (-0.037–0.332) |
|  | SI | 1.121 (0.923–1.361) | 0.677 (0.340–1.347) | 1.322 (0.734–2.381) | 1.175 (0.944–1.464) |

Abbreviations: RERI, relative excess risk due to interaction; AP, attributable proportion; SI, synergy index; CI, confidence interval; CVD, cardiovascular disease; BMI, body mass index; PA, physical activity; SHS, secondhand smoke; SNP, single nucleotide polymorphism.

Additive interactions between smoking-related exposures and IPF in relation to all-cause and cause-specific mortality during follow-up were evaluated using RERI, AP, and SI with 95% CIs from Model 3. Model 3 was adjusted for sex, age at recruitment, body mass index (BMI), household income, educational attainment, ethnicity, Townsend deprivation index quartiles, physical activity (PA), alcohol consumption, diet, and candidate SNPs. “Ever” indicates any exposure versus never exposure (reference). For smoking status, the exposed category was current smoking and the reference category was non-current smoking, which included never and former smokers. For passive smoking exposures, exposed participants were compared with unexposed participants. Boldface indicates RERI estimates for which the 95% confidence interval does not include zero.

**Supplementary Table S22. Multiplicative interactions between smoking-related exposures and IPF in relation to all-cause and cause-specific mortality during follow-up after excluding events within the first two years of follow-up**

| **interaction** | **HR (95% CI)** | | | |
| --- | --- | --- | --- | --- |
|  | **All-cause mortality** | **CVD mortality** | **Cancer mortality** | **Respiratory mortality** |
| Smoking status | 0.941 (0.650–1.362) | 0.437 (0.106–1.799) | 1.412 (0.736–2.709) | 0.948 (0.509–1.766) |
| Adult SHS exposure | 1.005 (0.850–1.189) | 0.691 (0.421–1.133) | 1.082 (0.762–1.535) | 0.853 (0.644–1.129) |
| Maternal smoking exposure | 1.073 (0.908–1.267) | 0.822 (0.502–1.348) | 1.079 (0.765–1.521) | 1.046 (0.794–1.377) |
| Passive SHS exposure | 1.026 (0.878–1.200) | 0.728 (0.470–1.129) | 1.137 (0.822–1.572) | 0.826 (0.635–1.074) |

Abbreviations: HR, hazard ratio; CI, confidence interval; CVD, cardiovascular disease; BMI, body mass index; PA, physical activity; SHS, secondhand smoke; SNP, single nucleotide polymorphism.

Multiplicative interactions between smoking-related exposures and IPF in relation to all-cause and cause-specific mortality during follow-up were evaluated using the HR and 95% CI for the interaction term from Model 3. Model 3 was adjusted for sex, age at recruitment, body mass index (BMI), household income, educational attainment, ethnicity, Townsend deprivation index quartiles, physical activity (PA), alcohol consumption, diet, and candidate SNPs. For smoking status, the exposed category was current smoking and the reference category was non-current smoking, which included never and former smokers. For passive smoking exposures, exposed participants were compared with unexposed participants. Statistical significance is indicated as *P < 0.05, **P < 0.01, and ***P < 0.001.

**Supplementary Table S23. Associations of prevalent IPF at baseline and incident IPF during follow-up with all-cause and cause-specific mortality**

| **Exposure** | **Person-years**  **(PYs)** | **Cases/N** | **Incidence Rate**  **(95% CI)** | **HR (95% CI)** | | |
| --- | --- | --- | --- | --- | --- | --- |
|  |  |  |  | **Model 1** | **Model 2** | **Model 3** |
| **All-cause mortality** |  |  |  |  |  |  |
| No IPF | 3629780.01 | 20277/269313 | 5.586 (5.510–5.664) | 1.000 (ref) | 1.000 (ref) | 1.000 (ref) |
| Prevalent IPF at baseline | 488.11 | 30/50 | 61.462 (41.468–87.741) | 12.392 (8.662–17.729) *** | 7.386 (5.161–10.569) *** | 7.373 (5.153–10.551) *** |
| Incident IPF during follow-up | 14572.10 | 600/1229 | 41.175 (37.945–44.605) | 7.844 (7.232–8.507) *** | 3.986 (3.673–4.326) *** | 3.963 (3.650–4.303) *** |
| **CVD mortality** |  |  |  |  |  |  |
| No IPF | 3629780.01 | 3643/269313 | 1.004 (0.971–1.037) | 1.000 (ref) | 1.000 (ref) | 1.000 (ref) |
| Prevalent IPF at baseline | 488.11 | 5/50 | 10.244 (3.326–23.905) | 11.673 (4.855–28.063) *** | 5.869 (2.440–14.119) *** | 5.848 (2.431–14.069) *** |
| Incident IPF during follow-up | 14572.10 | 76/1229 | 5.215 (4.109–6.528) | 5.582 (4.448–7.006) *** | 2.323 (1.849–2.918) *** | 2.280 (1.813–2.868) *** |
| **Cancer mortality** |  |  |  |  |  |  |
| No IPF | 3629780.01 | 10756/269313 | 2.963 (2.908–3.020) | 1.000 (ref) | 1.000 (ref) | 1.000 (ref) |
| Prevalent IPF at baseline | 488.11 | 4/50 | 8.195 (2.233–20.982) | 2.988 (1.121–7.962) *** | 1.969 (0.739–5.248) | 1.968 (0.738–5.245) |
| Incident IPF during follow-up | 14572.10 | 145/1229 | 9.951 (8.397–11.708) | 3.491 (2.963–4.112) *** | 2.017 (1.711–2.378) *** | 2.005 (1.700–2.364) *** |
| **Respiratory mortality** |  |  |  |  |  |  |
| No IPF | 3629780.01 | 723/269313 | 0.199 (0.185–0.214) | 1.000 (ref) | 1.000 (ref) | 1.000 (ref) |
| Prevalent IPF at baseline | 488.11 | 16/50 | 32.780 (18.736–53.232) | 185.168 (112.829–303.886) *** | 97.970 (59.501–161.311) *** | 97.106 (58.959–159.933) *** |
| Incident IPF during follow-up | 14572.10 | 304/1229 | 20.862 (18.582–23.344) | 109.019 (95.403–124.578) *** | 45.809 (39.818–52.701) *** | 44.334 (38.351–51.250)*** |

Abbreviations: PYs, person-years; Cases/N, number of deaths/total number of participants; CI, confidence interval; HR, hazard ratio; IPF, idiopathic pulmonary fibrosis; CVD, cardiovascular disease; BMI, body mass index; PA, physical activity; SNP, single nucleotide polymorphism. Incidence rates are presented as events per 1,000 person-years, and “ref” indicates the reference category.

Hazard ratios (HRs) and 95% confidence intervals (CIs) were estimated using Cox proportional hazards regression. Model 1 was unadjusted. Model 2 was adjusted for sex, age at recruitment, body mass index (BMI), household income, educational attainment, ethnicity, Townsend deprivation index quartiles, physical activity (PA), alcohol consumption, and diet. Model 3 was additionally adjusted for candidate SNPs. Prevalent IPF at baseline was defined as IPF diagnosed before recruitment, and incident IPF during follow-up was defined as IPF newly diagnosed after baseline during follow-up. Participants without IPF served as the reference group. Participants whose IPF status was identified only from death-registry records, without a reliably classifiable pre-baseline or post-baseline diagnosis date, were excluded from the prevalent-versus-incident IPF analysis and were not included in the no-IPF reference group. Statistical significance is indicated as *P < 0.05, **P < 0.01, and ***P < 0.001.

**Supplementary Table S24. Joint associations of smoking-related exposures and time-varying IPF with all-cause and cause-specific mortality during follow-up**

| **Joint variables** | **Cases/N** | **HR (95% CI)** | | |
| --- | --- | --- | --- | --- |
|  |  | **Model 1** | **Model 2** | **Model 3** |
| **All-cause mortality** |  |  |  |  |
| Smoking status |  |  |  |  |
| Non-current * No IPF | 19675/262912 | 1.000 (ref) | 1.000 (ref) | 1.000 (ref) |
| Non-current * IPF | 603/1228 | 24.131 (21.849–26.652)*** | 12.469 (11.206–13.874)*** | 12.430 (11.167–13.835)*** |
| Current * No IPF | 654/7682 | 1.143 (1.057–1.236)*** | 1.289 (1.192–1.394)*** | 1.289 (1.192–1.394)*** |
| Current * IPF | 27/51 | 22.016 (14.048–34.502)*** | 12.525 (7.343–21.363)*** | 12.498 (7.328–21.316)*** |
| Adult SHS exposure |  |  |  |  |
| Never * No IPF | 15269/210673 | 1.000 (ref) | 1.000 (ref) | 1.000 (ref) |
| Never * IPF | 433/912 | 23.221 (20.696–26.054)*** | 12.155 (10.759–13.733)*** | 12.111 (10.716–13.687)*** |
| Ever * No IPF | 5060/59921 | 1.167 (1.130–1.204)*** | 1.119 (1.083–1.155)*** | 1.119 (1.083–1.155)*** |
| Ever * IPF | 197/367 | 29.250 (24.470–34.964)*** | 14.279 (11.679–17.458)*** | 14.257 (11.669–17.418)*** |
| Maternal smoking exposure |  |  |  |  |
| Never * No IPF | 14385/191417 | 1.000 (ref) | 1.000 (ref) | 1.000 (ref) |
| Never * IPF | 415/849 | 23.862 (21.181–26.881)*** | 11.985 (10.531–13.640)*** | 11.952 (10.501–13.603)*** |
| Ever * No IPF | 5944/79177 | 0.997 (0.967–1.027) | 1.040 (1.009–1.072)* | 1.040 (1.008–1.072)* |
| Ever * IPF | 215/430 | 24.007 (20.351–28.320)*** | 13.709 (11.506–16.334)*** | 13.656 (11.458–16.276)*** |
| **CVD mortality** |  |  |  |  |
| Smoking status |  |  |  |  |
| Non-current * No IPF | 3513/262912 | 1.000 (ref) | 1.000 (ref) | 1.000 (ref) |
| Non-current * IPF | 79/1228 | 17.118 (13.592–21.558)*** | 7.289 (5.745–9.247)*** | 7.174 (5.649–9.112)*** |
| Current * No IPF | 136/7682 | 1.331 (1.121–1.580)** | 1.440 (1.212–1.712)*** | 1.440 (1.211–1.711)*** |
| Current * IPF | 2/51 | 8.794 (2.186–35.377)** | 3.852 (0.906–16.375) | 3.819 (0.899–16.231) |
| Adult SHS exposure |  |  |  |  |
| Never * No IPF | 2645/210673 | 1.000 (ref) | 1.000 (ref) | 1.000 (ref) |
| Never * IPF | 58/912 | 17.370 (13.311–22.668)*** | 7.602 (5.794–9.973)*** | 7.482 (5.698–9.825)*** |
| Ever * No IPF | 1004/59921 | 1.335 (1.242–1.436)*** | 1.175 (1.091–1.265)*** | 1.174 (1.090–1.265)*** |
| Ever * IPF | 23/367 | 19.010 (12.353–29.254)*** | 6.857 (4.363–10.778)*** | 6.752 (4.292–10.620)*** |
| Maternal smoking exposure |  |  |  |  |
| Never * No IPF | 2545/191417 | 1.000 (ref) | 1.000 (ref) | 1.000 (ref) |
| Never * IPF | 59/849 | 18.520 (14.201–24.152)*** | 7.533 (5.722–9.917)*** | 7.427 (5.637–9.786)*** |
| Ever * No IPF | 1104/79177 | 1.046 (0.974–1.122) | 1.089 (1.014–1.170)* | 1.089 (1.013–1.170)* |
| Ever * IPF | 22/430 | 13.439 (8.706–20.745)*** | 6.538 (4.186–10.211)*** | 6.414 (4.101–10.031)*** |
| **Cancer mortality** |  |  |  |  |
| Smoking status |  |  |  |  |
| Non-current * No IPF | 10436/262912 | 1.000 (ref) | 1.000 (ref) | 1.000 (ref) |
| Non-current * IPF | 140/1228 | 11.572 (9.681–13.831)*** | 6.779 (5.651–8.131)*** | 6.753 (5.627–8.105)*** |
| Current * No IPF | 327/7682 | 1.078 (0.965–1.203) | 1.238 (1.108–1.382)*** | 1.238 (1.109–1.382)*** |
| Current * IPF | 9/51 | 15.150 (7.419–30.936)*** | 9.982 (4.694–21.226)*** | 9.970 (4.691–21.192)*** |
| Adult SHS exposure |  |  |  |  |
| Never * No IPF | 8270/210673 | 1.000 (ref) | 1.000 (ref) | 1.000 (ref) |
| Never * IPF | 104/912 | 11.289 (9.191–13.865)*** | 6.681 (5.419–8.236)*** | 6.658 (5.398–8.210)*** |
| Ever * No IPF | 2493/59921 | 1.062 (1.015–1.110)** | 1.068 (1.020–1.118)** | 1.068 (1.020–1.118)** |
| Ever * IPF | 45/367 | 13.490 (9.813–18.546)*** | 7.804 (5.633–10.811)*** | 7.775 (5.610–10.775)*** |
| Maternal smoking exposure |  |  |  |  |
| Never * No IPF | 7656/191417 | 1.000 (ref) | 1.000 (ref) | 1.000 (ref) |
| Never * IPF | 99/849 | 11.754 (9.507–14.531)*** | 6.760 (5.442–8.396)*** | 6.735 (5.421–8.369)*** |
| Ever * No IPF | 3107/79177 | 0.979 (0.939–1.021) | 1.018 (0.976–1.062) | 1.018 (0.976–1.062) |
| Ever * IPF | 50/430 | 11.429 (8.492–15.383)*** | 7.222 (5.345–9.758)*** | 7.193 (5.323–9.722)*** |
| **Respiratory mortality** |  |  |  |  |
| Smoking status |  |  |  |  |
| Non-current * No IPF | 728/262912 | 1.000 (ref) | 1.000 (ref) | 1.000 (ref) |
| Non-current * IPF | 305/1228 | 337.716 (288.853–394.845)*** | 144.796 (121.146–173.063)*** | 141.688 (118.216–169.821)*** |
| Current * No IPF | 28/7682 | 1.323 (0.908–1.930) | 1.457 (0.997–2.128) | 1.455 (0.996–2.125) |
| Current * IPF | 15/51 | 341.931 (196.535–594.890)*** | 171.269 (91.529–320.478)*** | 160.584 (85.987–299.895)*** |
| Adult SHS exposure |  |  |  |  |
| Never * No IPF | 531/210673 | 1.000 (ref) | 1.000 (ref) | 1.000 (ref) |
| Never * IPF | 217/912 | 342.164 (285.728–409.747)*** | 146.609 (119.863–179.323)*** | 142.839 (116.647–174.912)*** |
| Ever * No IPF | 225/59921 | 1.493 (1.278–1.745)*** | 1.349 (1.150–1.582)*** | 1.350 (1.152–1.583)*** |
| Ever * IPF | 103/367 | 452.831 (356.258–575.584)*** | 184.917 (139.491–245.134)*** | 182.438 (137.376–242.281)*** |
| Maternal smoking exposure |  |  |  |  |
| Never * No IPF | 520/191417 | 1.000 (ref) | 1.000 (ref) | 1.000 (ref) |
| Never * IPF | 198/849 | 323.152 (267.781–389.971)*** | 132.958 (107.390–164.613)*** | 129.873 (104.681–161.128)*** |
| Ever * No IPF | 236/79177 | 1.095 (0.939–1.277) | 1.163 (0.995–1.359) | 1.160 (0.993–1.355) |
| Ever * IPF | 122/430 | 384.439 (307.800–480.159)*** | 190.584 (148.796–244.107)*** | 186.608 (145.215–239.800)*** |

Abbreviations: Cases/N, number of deaths/unique participant counts; HR, hazard ratio; CI, confidence interval; CVD, cardiovascular disease; SHS, secondhand smoke; “ref” indicates the reference category.

Hazard ratios (HRs) and 95% confidence intervals (CIs) were estimated using Cox proportional hazards regression. Model 1 was unadjusted. Model 2 was adjusted for sex, age at recruitment, body mass index (BMI), household income, educational attainment, ethnicity, Townsend deprivation index quartiles, physical activity (PA), alcohol consumption, and diet. Model 3 was additionally adjusted for candidate SNPs. “Ever” indicates any exposure versus never exposure (reference). For smoking status, the exposed category was current smoking and the reference category was non-current smoking, which included never and former smokers. For passive smoking exposures, exposed participants were compared with unexposed participants. Pack-years were categorized as 0, 1–10, 11–20, 21–30, and >30; “Unknown” indicates missing pack-years information. Statistical significance is indicated as *P < 0.05, **P < 0.01, and ***P < 0.001.

**Supplementary Table S25. Additive interactions between smoking-related exposures and time-varying IPF in relation to all-cause and cause-specific mortality during follow-up**

| **Additive interaction** | **Index for additive interaction** | **All-cause mortality** | **CVD mortality** | **Cancer mortality** | **Respiratory mortality** |
| --- | --- | --- | --- | --- | --- |
| Smoking status | RERI | -0.221 (-7.015**–**6.573) | -3.795 (-9.571**–**1.981) | 2.979 (-4.628**–**10.586) | 18.441 (-82.479**–**119.362) |
|  | AP | -0.018 (-0.571**–**0.535) | -0.994 (-3.909**–**1.922) | 0.299 (-0.243**–**0.841) | 0.115 (-0.445**–**0.674) |
|  | SI | 0.981 (0.402**–**1.561) | 0.426 (0.415**–**1.268) | 1.497 (0.208**–**2.787) | 1.131 (0.412**–**1.850) |
| Adult SHS exposure | RERI | 2.027 (-1.157**–**5.212) | -0.905 (-4.532**–**2.722) | 1.049 (-1.821**–**3.920) | 39.248 (-12.664**–**91.160) |
|  | AP | 0.142 (-0.056**–**0.341) | -0.134 (-0.722**–**0.454) | 0.135 (-0.196**–**0.466) | 0.215 (-0.020**–**0.450) |
|  | SI | 1.181 (0.886**–**1.475) | 0.864 (0.340**–**1.388) | 1.183 (0.660**–**1.707) | 1.276 (0.891**–**1.661) |
| Maternal smoking exposure | RERI | 1.665 (-1.155**–**4.484) | -1.102 (-4.582**–**2.378) | 0.440 (-2.149**–**3.030) | **56.575 (9.341–103.809)** |
|  | AP | 0.122 (-0.067**–**0.311) | -0.172 (-0.778**–**0.434) | 0.061 (-0.284**–**0.406) | 0.303 (0.108**–**0.499) |
|  | SI | 1.151 (0.883**–**1.420) | 0.831 (0.325**–**1.337) | 1.077 (0.615**–**1.538) | 1.438 (1.032**–**1.845) |

Abbreviations: RERI, relative excess risk due to interaction; AP, attributable proportion; SI, synergy index; CI, confidence interval; CVD, cardiovascular disease; BMI, body mass index; PA, physical activity; SHS, secondhand smoke; SNP, single nucleotide polymorphism.

Additive interactions between smoking-related exposures and time-varying IPF in relation to all-cause and cause-specific mortality during follow-up were evaluated using RERI, AP, and SI with 95% CIs from Model 3. Model 3 was adjusted for sex, age at recruitment, body mass index (BMI), household income, educational attainment, ethnicity, Townsend deprivation index quartiles, physical activity (PA), alcohol consumption, diet, and candidate SNPs. For smoking status, the exposed category was current smoking and the reference category was non-current smoking, which included never and former smokers. For passive smoking exposures, exposed participants were compared with unexposed participants. Boldface indicates RERI estimates for which the 95% confidence interval does not include zero.

**Supplementary Table S26. Multiplicative interactions between smoking-related exposures and time-varying IPF in relation to all-cause and cause-specific mortality during follow-up**

| **interaction** | **HR (95% CI)** | | | |
| --- | --- | --- | --- | --- |
|  | **All-cause mortality** | **CVD mortality** | **Cancer mortality** | **Respiratory mortality** |
| Smoking status | 0.780 (0.450–1.351) | 0.370 (0.085–1.615) | 1.193 (0.546–2.606) | 0.779 (0.373–1.627) |
| Adult SHS exposure | 1.052 (0.833–1.330) | 0.768 (0.454–1.301) | 1.094 (0.743–1.610) | 0.946 (0.675–1.325) |
| Maternal smoking exposure | 0.662 (0.447–0.982) | 0.891 (0.397–1.996) | 0.814 (0.432–1.536) | 0.478 (0.264–0.866) |

Abbreviations: HR, hazard ratio; CI, confidence interval; CVD, cardiovascular disease; BMI, body mass index; PA, physical activity; SHS, secondhand smoke; SNP, single nucleotide polymorphism.

Multiplicative interactions between smoking-related exposures and IPF in relation to all-cause and cause-specific mortality during follow-up were evaluated using the HR and 95% CI for the interaction term from Model 3. Model 3 was adjusted for sex, age at recruitment, body mass index (BMI), household income, educational attainment, ethnicity, Townsend deprivation index quartiles, physical activity (PA), alcohol consumption, diet, and candidate SNPs. “Ever” indicates any exposure versus never exposure (reference). For smoking status, the exposed category was current smoking and the reference category was non-current smoking, which included never and former smokers. For passive smoking exposures, exposed participants were compared with unexposed participants. Statistical significance is indicated as *P < 0.05, **P < 0.01, and ***P < 0.001.
